# Identification of CHMP7 as a promising immunobiomarker for immunotherapy and chemotherapy and impact on prognosis of colorectal cancer patients

**DOI:** 10.3389/fcell.2023.1211843

**Published:** 2023-08-30

**Authors:** Yu Guo, Shu Wang, Feng Liang, Min Wang

**Affiliations:** ^1^ Department of the General Surgery, The Second Hospital of Jilin University, Changchun, China; ^2^ Department of the Ridiotherapy, The Second Hospital of Jilin University, Changchun, China

**Keywords:** pan-cancer, CHMP7, endosomal sorting complex required for transport, immunosuppression, CTL dysfunction, M2 macrophages infiltration

## Abstract

**Introduction:** ESCRT is a molecular machine involved in various important physiological processes, such as the formation of multivesicular bodies, cellular autophagy, and cellular membrane repair. *CHMP7* is a regulatory subunit of ESCRT-III and is necessary for the proper functioning of ESCRT. In this study, public databases were exploited to explore the role of *CHMP7* in tumors.

**Methods:** The research on *CHMP7* in oncology is rather limited. In this study, the differential expression of *CHMP7* in multiple tumor tissues was analyzed with information from public databases and clinically collected colorectal cancer tissue samples. Subsequently, the mutational landscape of *CHMP7*, methylation levels, and the relationship between its expression levels and genomic instability were resolved. The immune microenvironment is a compelling emerging star in tumor research. The correlation of *CHMP7* with various infiltrating immune cell types in TME was analyzed by online datasets and single-cell sequencing. In terms of clinical treatment, the impact of *CHMP7* expression levels on chemotherapy and immunotherapy and the evaluation of small molecule drugs related to *CHMP7* were assessed.

**Results:** *CHMP7* has a predictive value for the prognosis of patients with tumors and is highly involved in tumor immunity. The downregulation of *CHMP7* may lead to genomic instability. A strong correlation between *CHMP7* and TME immune cell infiltration has been observed, participating in the formation of suppressive TME and promoting tumor progression. The expression level of *CHMP7* is significantly lower in the non-responder group of multiple chemotherapeutic agents. *CHMP7* can potentially serve as a new biomarker for predicting the efficacy of tumor chemotherapy and immunotherapy.

**Conclusion:** As a gene of interest, *CHMP7* is expected to provide novel and promising targets for further treatment of patients with tumor.

## Graphical Abstract

**Figure:**
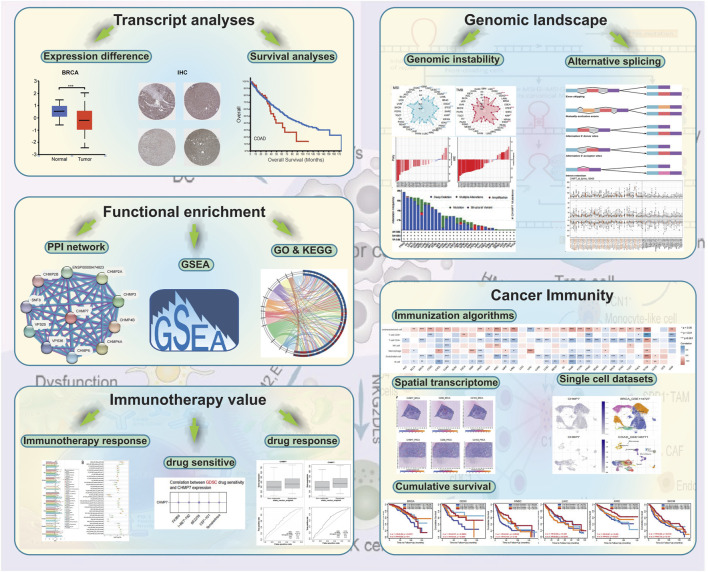
The main points of this study. The role of CHMP7 in COAD was analyzed in terms of differential expression, mutational analysis, functional enrichment analysis, and immune infiltration.

## Introduction

The formation of malignant tumors is an extremely complicated process that typically takes decades. Tissue cells in normal individuals evolve and progressively develop into tumor cells with a malignant phenotype, a process called tumor progression, usually accompanied by multiple genetic alterations (Hausman, 2019). Tumor progression is attributed to the accumulation of random mutations and epigenetic alterations in DNA sequences that affect the proliferation of malignant cells associated with gene regulatory networks and other traits associated with the malignant phenotype (Vogelstein et al., 2013; Iranzo et al., 2018). Tumorigenesis is usually the result of the synergistic effect of multiple risk factors, including environmental chemical factors (e.g., atmospheric pollutants), physical factors (e.g., ionizing radiation), viral infections, adverse dietary habits, and pharmaceutical effects (Lewandowska et al., 2019; Sun et al., 2020). Current treatment modalities for malignancies include surgery, chemotherapy, targeted drug therapy, radiation therapy, hormone therapy, stem cell transplantation, and tumor immunotherapy. However, various genomic mutations and epigenetic modifications occur in tumor cell DNA during progression, leading to the emergence of malignant phenotypes, including abnormal metabolism, treatment resistance, unrestricted division, and weakened intercellular adhesion, subsequently limiting the effectiveness of various therapeutic modalities and affecting the prognosis of patient survival. Tumor heterogeneity affects the sensitivity of different patients with the same tumor to chemotherapeutic agents, radiation therapy, and targeted drugs (Lim and Ma, 2019). In addition to tumor cells, there are several kinds of infiltrating cells in tumor tissue, such as cancer-associated fibroblasts (CAFs), B cells, T cells, macrophages, and other immune cells, adipocytes, and endothelial cells of blood vessels, which together with the tumor extracellular matrix (ECM) constitute the tumor microenvironment (TME). Different immune cells may perform different roles in tumorigenesis by inhibiting or promoting tumorigenesis (Lee and Cheah, 2019; Bilotta et al., 2022). The tumor progression is frequently characterized by mutations in multiple genes, and the development of high-throughput sequencing technology has become a practical approach to unraveling the mystery of cancer genes.

*CHMP7* (Charged multivesicular body protein 7) is essential in properly regulating the ESCRT (endosomal sorting complex required for transport). The ESCRT system is an integral molecular mechanism responsible for membrane protein sorting and membrane repair in eukaryotic cells, and it participates in several physiological processes, such as cell division and autophagy. Ubiquitin-tagged membrane proteins are primarily transported to the endosomal membrane through cytokinesis and then invaginated by the ESCRT system, which releases membrane components containing these proteins into the endosomal lumen to form intraluminal vesicles (ILVs). Subsequently, the ILVs and their membrane proteins are degraded through fusion with lysosomes, and the protein-bound ESCRT complex proteins are degraded and recycled (Korbei, 2022). For example, the epidermal growth factor receptor (EGFR) is degraded by this pathway. In addition to its involvement in the degradation of ubiquitinated proteins, the ESCRT system is also involved in the sorting and delivery of extracellular vehicles (EVs) (Wei et al., 2021) and in the repair of cell membranes to maintain cell integrity and normal function. It is also a vital component of membrane proteins in cellular life activities such as cell division and autophagy (Vietri et al., 2020; Ritter et al., 2022). All these physiological processes are involved in topological membrane remodeling. ESCRT-III is the key player in completing the critical step of the shearing of the budding body (Gatta and Carlton, 2019; Isono, 2021). The dysregulation of ESCRT function is highly related to tumor development, and CHMP7, an important regulatory subunit of ESCRT-III, has attracted our attention.

Aberrant expression of *CHMP7* may lead to nuclear pore complex damage and TDP-43 dysfunction in amyotrophic lateral sclerosis/frontotemporal dementia (Coyne et al., 2021). Neurodegenerative diseases, such as Huntington’s disease and Parkinson’s disease, are often associated with the accumulation of intracellular ubiquitinated protein aggregates, with lesions that may be associated with the loss of ESCRT function (Coyne and Rothstein, 2022). Regarding oncology research, Ritter *et al.* discovered that ESCRT-mediated cell membrane repair mechanisms contribute to the immune escape of cancer cells from lethal attacks by cytotoxic T lymphocytes (CTL). Furthermore, inhibiting the ESCRT pathway significantly improves the killing efficiency of CTL (Ritter et al., 2022). Abnormal expression of several genes in the ESCRT system is also assumed to be associated with tumors. *VPS4A* is significantly overexpressed in liver cancer tissues and can promote tumor growth and invasion by affecting the sorting and delivery of exosomes (Han et al., 2019). However, conclusive studies on the role of *CHMP7* in tumors are limited.

**FIGURE 1:**
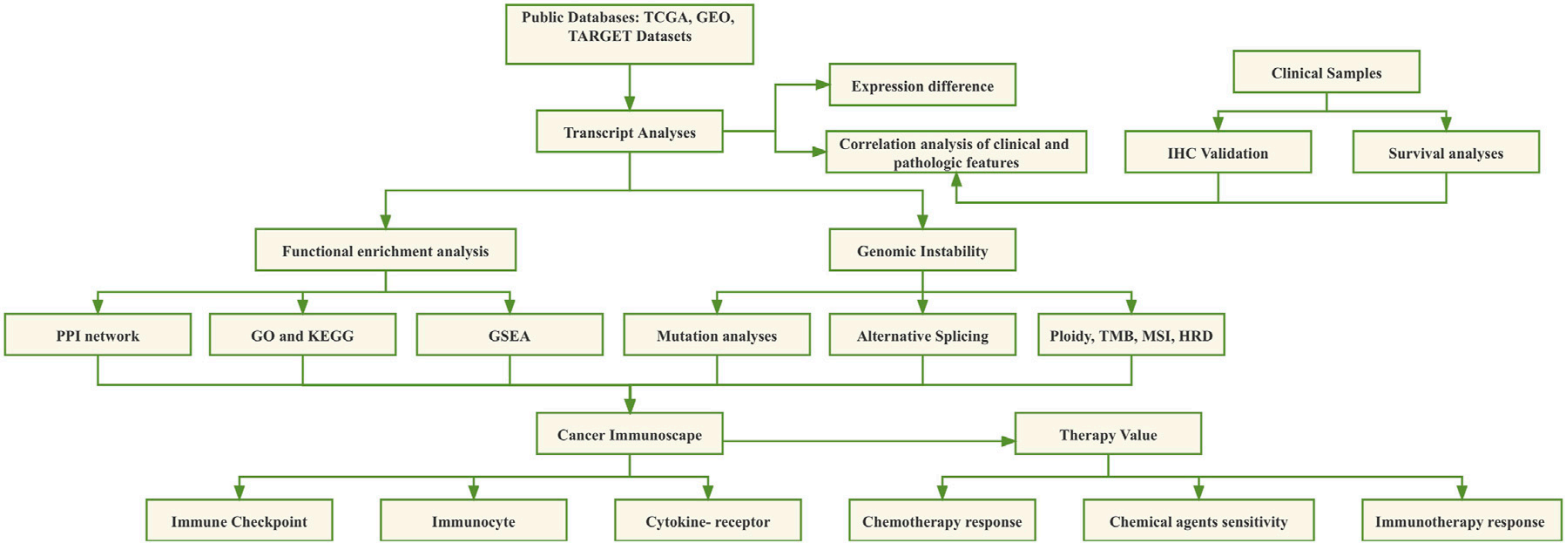
The flow chart of this study.

In this study, the differential expression of *CHMP7* in tumor tissues and its prognostic impact were systematically analyzed with information from public databases such as TCGA, CCLE, and GTEX. Immunohistochemical (IHC) validation was performed on clinically collected colorectal cancer tissue samples. Subsequently, the mutational landscape of *CHMP7*, methylation levels, and the relationship between its expression levels and genomic instability were resolved. The co-expression of *CHMP7* in various infiltrating immune cell types in TME was verified by an online dataset and single-cell sequencing analysis. The influence of *CHMP7* on chemotherapy and immunotherapy was evaluated to predict the effect of immunotherapy and sensitive drugs against *CHMP7* in these cancers (Figure 1).

## Methods

### Data source

The unified and standardized pan-cancer dataset was downloaded from the UCSC (https://xenabrowser.net/) database, Therapeutically Applicable Research to Generate Effective Treatments (TARGET; includes 7 pediatric cancers), and Genotype-Tissue Expression (GTEx; covers 54 normal tissues). Those samples with fewer than three tumor types were excluded from the analysis. Single-cell sequencing datasets were obtained from the Gene Expression Omnibus (GEO) database. They included breast invasive carcinoma (BRCA, GSE114727), colon adenocarcinoma (COAD, GSE146771), skin cutaneous melanoma (SKCM, GSE48190), liver hepatocellular carcinoma (LIHC, GSE98638), nasopharyngeal carcinoma (NPC, GSE150430), and kidney renal clear cell carcinoma (KIRC, GSE 111360).

### Differential expression and prognostic analysis

Diseases associated with *CHMP7* were analyzed with the Open Target website (https://www.opentargets.org/) and represented in bubble charts. The sample information obtained from UCSC was analyzed with R software (version 3.6.4) to calculate the difference in *CHMP7* expression between normal and tumor samples in each tumor, and the significance of differences was performed using the unpaired Wilcoxon rank sum and signed rank tests. Protein level data were obtained from the UALCAN database (http://ualcan.path.uab.edu/) and further utilized to reveal the differential expression of *CHMP7* in normal and tumors (Patra et al., 2021). The UALCAN database also facilitated further analysis of the correlation between *CHMP7* expression levels and tumor stage (Patra et al., 2023). The SURVIVAL package from the R software was applied to analyze the association between *CHMP7* expression and pan-cancer survival with risk tables.

### IHC validation

IHC staining analysis was performed on fifty specimens of postoperative paraffin slides that underwent surgical treatment at the Department of Colorectal Surgery, Second Hospital of Jilin University, from January 2017 to December 2017, and whose postoperative pathology was confirmed as colorectal cancer. The experiment was approved by the ethics committee. The antibody was obtained from Proteintech under item number 16424-1-AP for IHC staining. Paraffin slides were stained by the Servicebio company. The slides were dried and observed with a light microscope, and two professional pathologists were invited to determine the intensity of the staining. Relevant clinical information of patients was collected and followed up to determine whether there was any significant difference in the effect of different *CHMP7* expression intensities on the prognosis of patients.

### Mutational analysis of *CHMP7* and correlation with genomic instability

cBioPortal (https://www.cbioportal.org/) is a website that enables researchers to explore, visualize, and analyze multidimensional cancer genomic data. Using this website, we can easily explore genetic alterations in different tumor types, genes, and pathways. We queried the mutation characteristics of *CHMP7* in different tumor types and further explored the mutation types of the genes. The prognostic impact of *CHMP7* mutations on patients with tumors was evaluated through the “Comparison” module of the website.

To further analyze the correlation between *CHMP7* and genomic stability, the R software was used to compute the correlation between tumor mutation burden (TMB), microsatellite instability (MSI), homologous recombination deficiency (HRD), and neoantigen (NEO) data for each tumor and *CHMP7* expression levels. A heat map demonstrated the correlation between *CHMP7* expression levels and five mismatch repair genes (*MLH1*, *MSH2*, *MSH6*, *PMS2*, and *EPCAM*) in each tumor type (Levy et al., 2017).

### Analyses of tumor stemness and epigenetic modifications

The Stemness index is an indicator to assess the similarity of tumor cells to stem cells, which is associated with active biological processes in stem cells and a more advanced degree of tumor dedifferentiation. Wiznerowicz et al. constructed predictive models for multipotential stem cell samples from the PCBC dataset with the one-class logistic regression (OCLR) machine learning algorithm. The predictive model was subsequently applied to a training set such as TCGA to calculate the stemness score for each sample (Malta et al., 2018). The tumor stemness index calculated by mRNA expression and methylation signature was obtained from previous studies, and the stemness index and gene expression data of the samples and their correlation performed were further integrated (Kocaturk et al., 2019).

Homologous recombination repair (HRR), as one of the core DNA damage repair methods, is a DNA repair mechanism that maintains genome integrity to ensure high-fidelity transmission of genetic information. Mutation of related HRR genes or methylation of gene promoters can trigger HRR dysfunction, leading to genomic instability. Tumor cells tend to exploit HRR to save cells from apoptosis. HRR is a complex signaling pathway involving multiple steps, in which the most critical genes are *BRCA1* and *BRCA2*, and other related genes include *MLH1*, *MSH2*, *ATM*, and *TP53*. The HRR-related genes analyzed in the present study were cited in the research of Liang et al. (Liang et al., 2022). The correlation between *CHMP7* and HRR signature can be analyzed by applying the GEPIA2.0 online website (Ledermann et al., 2016). Heat maps were applied to visualize the correlation between *CHMP7* and 44 RNA-modified genes.

### Alternative splicing (AS) analysis

AS refers to the process of mRNA precursor to mature mRNA, in which various splicing methods can allow the same gene to produce multiple different mature mRNAs, resulting in the translation of different proteins (Ule and Blencowe, 2019). AS is a major mechanism for maintaining protein diversity (Li et al., 2017). It produces specific shear isoforms in certain tissues or conditions at different stages of development. The OncoSplicing website (http://www.oncosplicing.com/) was employed to explore SplAdder and SliceSeq projects that contain AS events for *CHMP7*. Differences in percent spliced-in (PSI) and AS events were further compared between TCGA tumor tissues and GTEx normal tissues. We also explored the impact of AS events of *CHMP7* on patient prognosis in diverse tumors and confirmed shear isoforms of *CHMP7* in pan-cancer.

### Functional enrichment analysis

The protein-protein interaction (PPI) network of *CHMP7* was explored using the STRING website (https://www.string-db.org/). Tumor development involves the aberrant activation of multiple critical pathways. Genes in the corresponding pathways were collected from TCGA and analyzed using the GSVA package of R software. The correlation between *CHMP7* and pathway scores was analyzed using Spearman correlation analysis. The similar gene detection function in the GEPIA2.0 website helped us obtain genes that were the top 100 co-expressed with *CHMP7* in tumor tissues and performed gene ontology (GO) functional analysis of the top 100 genes with the clusterProfiler package. Gene set enrichment analysis (GSEA) further explored the functional enrichment-related pathways *CHMP7* may affect.

### Immune infiltration analysis

The role of immune cells in TME has attracted much attention in recent years (Padma et al., 2023). Different immune cells perform various roles in tumorigenesis. The immune cells of tumor types are frequently quantified in studies of tumorigenesis, treatment, and other mechanisms. The correlation scores between *CHMP7* and tumor tissue immune infiltration levels were calculated with the ESTIMATE package of R software, and the bar graphs present the stromal, immune, and ESTIMATE scores for each tumor (Yoshihara et al., 2013). The correlation between *CHMP7* and immune checkpoints is also shown in a heat map.

The TISDB online website (http://cis.hku.hk/TISIDB/) was utilized to analyze the differential expression of *CHMP7* in distinct immune subtypes of pan-cancerous tissues (Thorsson et al., 2018). The site was also exploited to construct heat maps exhibiting the correlation between *CHMP7* and chemokines, chemokine receptors, immunostimulatory factors, and immunoinhibitory factors.

M2 macrophages are regarded to serve in pathogen clearance, anti-inflammatory response, and tumor progression (Biswas and Mantovani, 2010). The TIMER2.0 website was applied to analyze the relationship between *CHMP7* and M2 macrophages according to different algorithms. Additionally, spatial transcripts in SpatialDB (https://www.spatialomics.org/SpatialDB/) were subjected to analysis of the overlapping levels of *CHMP7* expression and spatial expression of M2 macrophage markers (CD68 and CD163). As for the TISCH website (http://tisch.comp-genomics.org/), single-cell data from multiple tumors were presented to compare the expression of *CHMP7* in various cell subtypes of tumor tissues.

CTLs are one of the critical immune surveillance cells. A high abundance of CTL with a killing function in tumor tissues is a promising prognostic indicator, and increasing the proportion of CTL in patients’ tumor tissues can help inhibit tumor progression and eventual elimination (Mami-Chouaib et al., 2018). Multiple algorithms are available on the TIMER2.0 website to analyze the correlation between *CHMP7* and CTL and the impact of *CHMP7* expression levels and CTL infiltration levels on patient prognosis. Tumor immune dysfunction and exclusion (TIDE, a web tool) also enabled us to investigate the role of *CHMP7* in T cell dysfunction and CTL-related prognosis in tumor tissues.

### Drug sensitivity analysis

To elucidate the predictive value of *CHMP7* in tumor immunotherapy, we explored it from public databases. The predictive role of *CHMP7* as a new biomarker was compared with classical markers such as TMB at the TIDE website to calculate the predictive role of *CHMP7*. The TIDE website further enabled us to predict the therapeutic response of *CHMP7* in the core dataset, immunotherapy dataset, CRISPR screening dataset, and mechanistic follow-up experiments of immunosuppressive cell types. And the differential expression of *CHMP7* was compared between the responding and non-responding groups in 30 immunotherapy cohorts such as IMvigor210.

The ROC Plotter dataset (http://www.rocplot.org/site/index) was adopted to analyze the gene expression levels in the response and non-response groups of multiple chemotherapeutic agents. The RNAactDrug database (hrbmu.edu.cn) can facilitate our queries on the association between *CHMP7* and drug sensitivity (Xie et al., 2022).

### Statistical analysis

The overall survival (OS) differences between the high and low gene expression groups were explored using the log-rank test. The correlation coefficients were quantified using Spearman or Pearson. All data analyses included in this article, such as differential expression, gene interaction, immune infiltration, and drug sensitivity analyses, were considered significant only at *p* < 0.05.

## Results

### *CHMP7* is aberrantly expressed in multiple tumor tissues and correlates with patient prognosis

**FIGURE 2:**
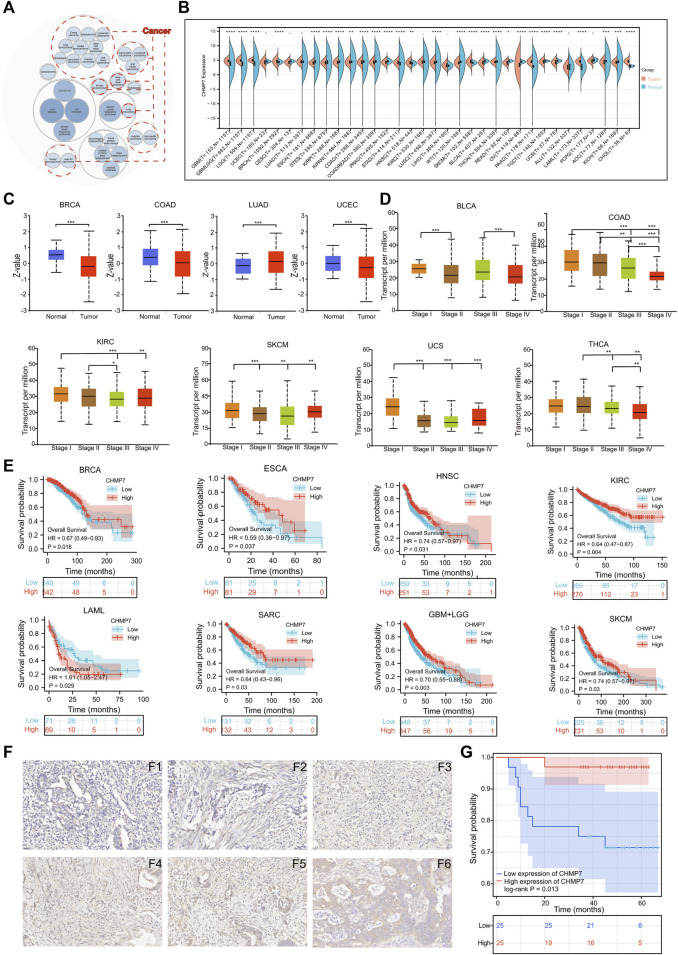
Differential expression and prognostic analysis. **(A)** Diseases associated with *CHMP7* were analyzed with the openTarget website. And tumors that may be associated with *CHMP7* are marked with red dotted lines. **(B)** Analysis of the TCGA, TARGET and GTEx databases for differential expression of *CHMP7* in tumor tissues and corresponding normal tissues. **(C)** The UALCAN website facilitates the analysis of differential expression of *CHMP7* in different tumor stages. **(D)** *CHMP7* expression levels of multiple tumors at different stages were further analyzed on UALCAN. **(E)** Kaplan-Meier curves were plotted for the analysis of *CHMP7* expression in relation to patient prognosis. **(F)** IHC staining maps of COAD tissues. F_1_-F_3_ is the low *CHMP7* expression group and F_4_-F_6_ is the high group with a magnification of ×40. **(G)** The OS of COAD patients that performed IHC staining. (*: *p* < 0.05, **: *p* < 0.01 and ***: *p* < 0.001).

The Open Target website presents diseases associated with *CHMP7*, and the bubble chart illustrates that *CHMP7* may be associated with multiple tumors (Figure 2A). The differential expression of *CHMP7* in tumor tissues and corresponding normal tissues were analyzed with TCGA, TARGET, and GTEx databases. The results indicated that *CHMP7* expression levels were significantly upregulated in GBM, LGG, BRCA, KIPAN, STAD, HNSC, SKCM, PAAD, LAML, and CHOL tumor tissues, while markedly downregulated in ESCA, STES, KIRP, COAD, PRAD, LUSC, BLCA, THCA, READ, OV, TGCT, UCS, ACC, and KICH tumor tissues (Figure 2B). The UALCAN website analyzed the differences in *CHMP7* protein levels, revealing that *CHMP7* protein was downregulated in BRCA, COAD, and UCEC tumor tissues and upregulated in LUAD tumor tissues (Figure 2C). The expression level of *CHMP7* is related to the stage of many tumors. For example, the more advanced the stage in COAD, the lower the *CHMP7* expression level (Figure 2D). The TCGA data were employed to analyze the predictive value of *CHMP7* on prognosis and demonstrated that low *CHMP7* expression was associated with poorer OS in BRCA, ESCA, HNSC, KIRC, SARC, and SKCM, while in LAML, patients in the high *CHMP7* expression group had a worse prognosis (Figure 2E).

The IHC staining was performed on the collected 50 colorectal cancer paraffin slides. According to the staining intensity, specimens were divided into *CHMP7* low expression group (Figures 2F1–3) and *CHMP7* high expression group (Figures 2F4–6). The relevant information was collected into a baseline clinical information table (Table 1), and the collected data were processed and analyzed using R software. The results indicated that *CHMP7* expression level was not significantly correlated with patients’ age, weight, gender, concomitant disease status (hypertension and diabetes), and T-stage, and the group with low *CHMP7* expression had advanced N-stage and TNM-stage. The prognostic impact of *CHMP7* on patients was analyzed by Kaplan-Meier survival analysis, which revealed that patients with low *CHMP7* expression had a relatively poor prognosis (Figure 2G).

**TABLE 1:** Baseline table of clinical information of COAD patients who performed IHC staining.

Characteristics	High expression of CHMP7	Low expression of CHMP7	Total (*N* = 50)	*p* value	FDR
	(*N* = 25)	(*N* = 25)			
Age					
Mean ± SD	62.36 ± 11.85	61.32 ± 10.97	61.84 ± 11.31		
Median [min-max]	62.00 [37.00,85.00]	62.00 [42.00,84.00]	62.00 [37.00,85.00]		
Weight					
Mean ± SD	68.88 ± 13.16	66.82 ± 13.06	67.85 ± 13.02		
Median [min-max]	70.00 [40.00,94.00]	67.00 [43.00,100.00]	69.50 [40.00,100.00]		
Sex				1	1
Female	6 (12.00%)	5 (10.00%)	11 (22.00%)		
Male	19 (38.00%)	20 (40.00%)	39 (78.00%)		
T.stage				0.11	0.76
T1	3 (6.00%)	0 (0.0e+0%)	3 (6.00%)		
T2	6 (12.00%)	3 (6.00%)	9 (18.00%)		
T3	16 (32.00%)	17 (34.00%)	33 (66.00%)		
T4	0 (0.0e+0%)	1 (2.00%)	1 (2.00%)		
T4a	0 (0.0e+0%)	3 (6.00%)	3 (6.00%)		
T4b	0 (0.0e+0%)	1 (2.00%)	1 (2.00%)		
N.stage				0.07	0.52
N0	19 (38.00%)	9 (18.00%)	28 (56.00%)		
N1	1 (2.00%)	1 (2.00%)	2 (4.00%)		
N1a	0 (0.0e+0%)	2 (4.00%)	2 (4.00%)		
N1b	1 (2.00%)	5 (10.00%)	6 (12.00%)		
N1c	0 (0.0e+0%)	2 (4.00%)	2 (4.00%)		
N2a	2 (4.00%)	5 (10.00%)	7 (14.00%)		
N2b	2 (4.00%)	1 (2.00%)	3 (6.00%)		
M.stage				0.23	1
M0	25 (50.00%)	22 (44.00%)	47 (94.00%)		
M1	0 (0.0e+0%)	3 (6.00%)	3 (6.00%)		
TNM.stage				4.40E-03	0.04
1	9 (18.00%)	1 (2.00%)	10 (20.00%)		
2	10 (20.00%)	7 (14.00%)	17 (34.00%)		
3	6 (12.00%)	14 (28.00%)	20 (40.00%)		
4	0 (0.0e+0%)	3 (6.00%)	3 (6.00%)		
Hypertension				1	1
No	20 (40.00%)	21 (42.00%)	41 (82.00%)		
Yes	5 (10.00%)	4 (8.00%)	9 (18.00%)		
Diabetes mellitus				1	1
No	21 (42.00%)	20 (40.00%)	41 (82.00%)		
Yes	4 (8.00%)	5 (10.00%)	9 (18.00%)		
Vascular invasion				0.12	0.76
No	21 (42.00%)	15 (30.00%)	36 (72.00%)		
Yes	4 (8.00%)	10 (20.00%)	14 (28.00%)		
CEA					
Mean ± SD	9.71 ± 16.19	9.43 ± 13.14	9.57 ± 14.59		
Median [min-max]	3.52 [0.35,63.79]	4.14 [0.71,46.33]	3.89 [0.35,63.79]		
EGFR status				0.77	1
negative	8 (16.33%)	6 (12.24%)	14 (28.57%)		
positive	12 (24.49%)	14 (28.57%)	26 (53.06%)		
weakly positive	5 (10.20%)	4 (8.16%)	9 (18.37%)		

### *CHMP7* is associated with various pathways, such as angiogenesis and apoptosis

**FIGURE 3:**
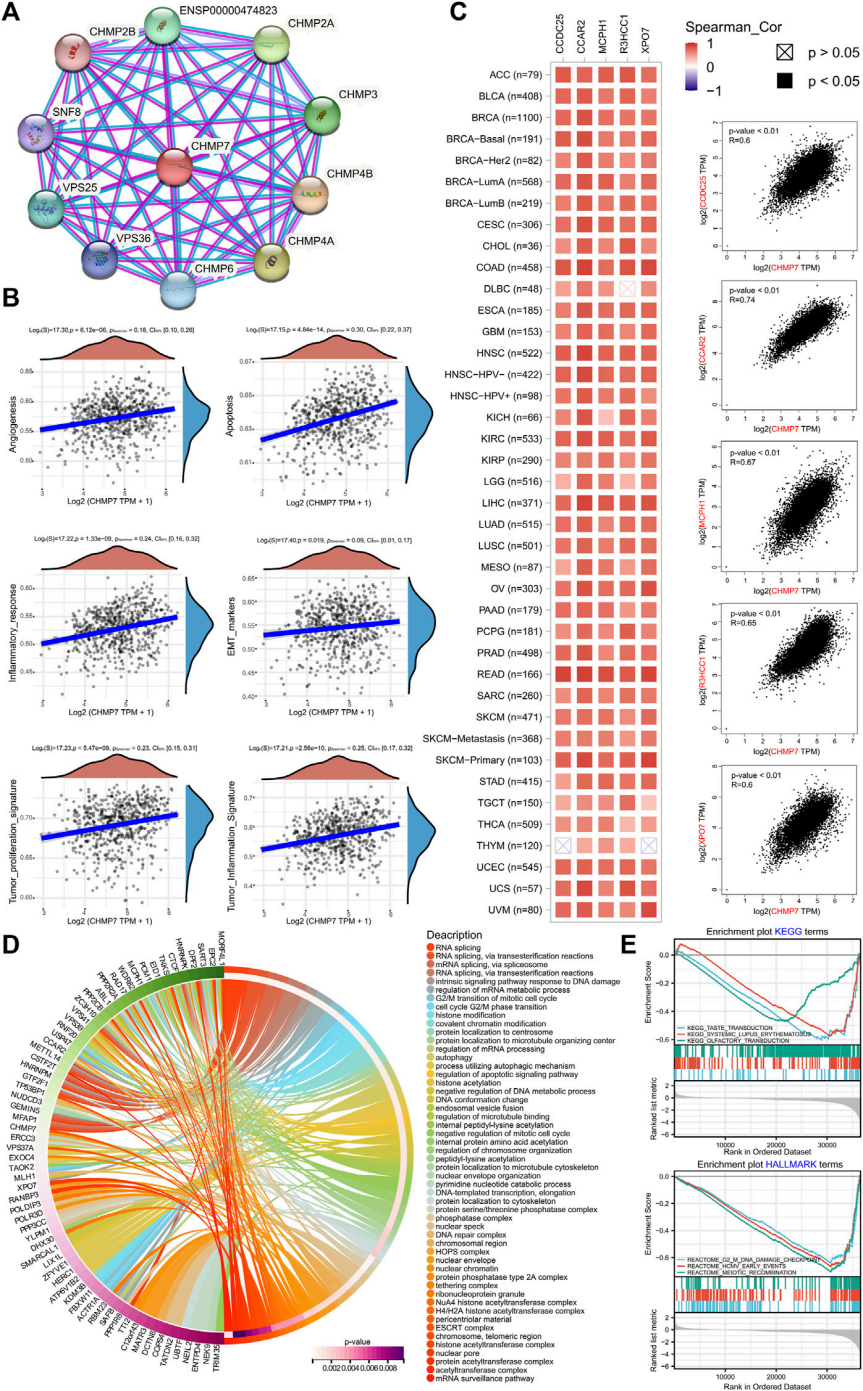
*CHMP7* functional enrichment analysis. **(A)** The String site reveals proteins interacting with *CHMP7*. **(B)** *CHMP7* expression levels correlate with the angiogenesis, apoptosis, inflammatory response, EMT markers, tumor proliferation signature, and tumor inflammation signature pathway. **(C)** The correlation between *CHMP7* and the top 5 co-expressed genes identified on GEPIA2.0. Each cancer type is shown on the left, and all cancer samples are on the right. **(D)** GO enrichment analysis circle plot of the top 100 *CHMP7* co-expressed genes identified on GEPIA2.0. **(E)** Enrichment plot of GSEA analysis of *CHMP7* and its associated genes KEGG and HALLMARK terms.

To explore the possible role of *CHMP7* in tumor development in detail, the enrichment pathways of *CHMP7* and its related genes were analyzed. The PPI network for *CHMP7* was constructed with the STRING website (Figure 3A). The tumor data from the TCGA database were exploited to analyze the correlations between *CHMP7* and multiple pathways, and the results were represented as scatter plots. *CHMP7* and angiogenesis, apoptosis, inflammatory response, EMT markers, tumor proliferation signature, and tumor inflammation signature all demonstrated a significant positive correlation (Figure 3B).

The top 100 genes tightly associated with *CHMP7* were explored via the GEPIA website, and heat maps were drawn to present the top 5 genes of relevance (Figure 3C; Supplementary Material S1). The genes of *CHMP7* and its related top hundred were subjected to GO and KEGG enrichment analysis, revealing a strong association with RNA splicing, ESCRT complex, and DNA damage checkpoint (Figures 3D, E).

### *CHMP7* is altered in multiple tumor tissues and associated with genomic instability

**FIGURE 4:**
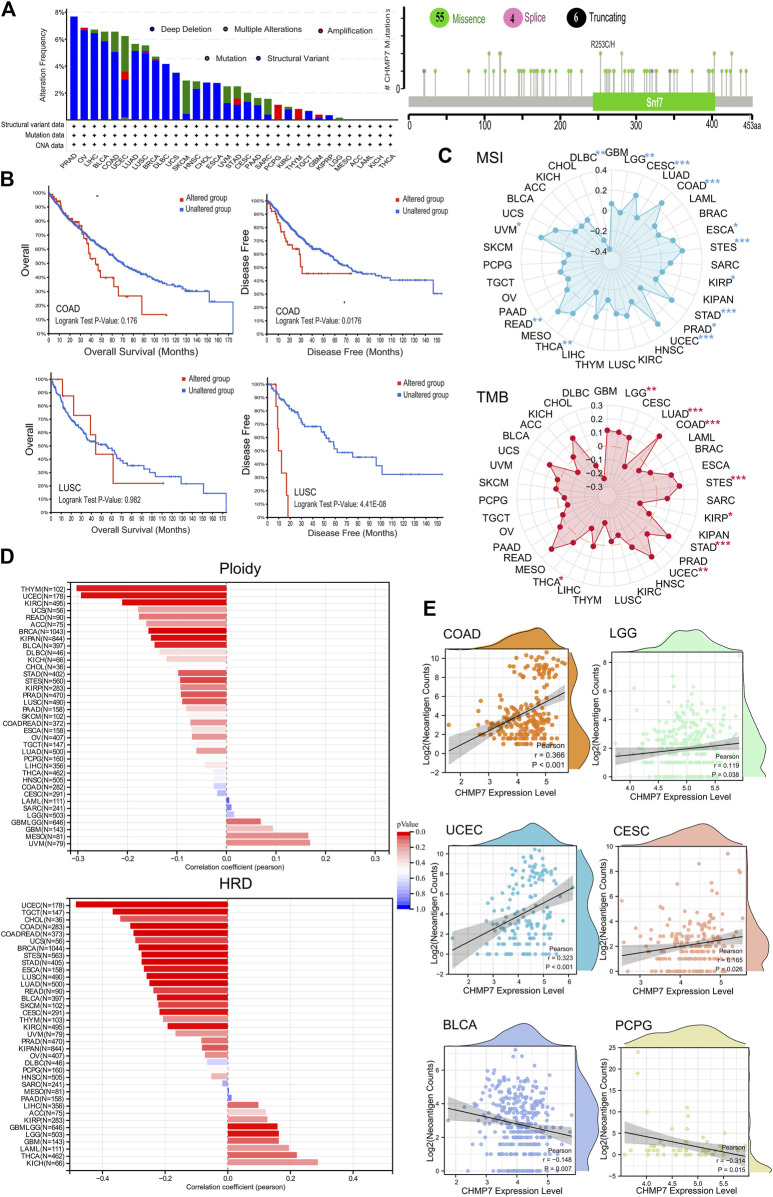
*CHMP7* is correlated with genomic instability in tumor tissues. **(A)** Genomic alterations of *CHMP7* in TCGA pan-cancer and the loci at which missense mutations, frameshift deletions and splicing occur. **(B)** Impact of *CHMP7* mutations on patient prognosis, with poorer prognosis in the mutated group of COAD and LUSC patients. **(C)** Radar plot showing the association between *CHMP7* expression levels and TMB (top) and MSI (bottom) in pan-cancer; correlation coefficients of 0 are indicated by dashed circles, points inside the dashed circles indicate negative correlation coefficients, those outside the circles indicate positive correlation coefficients, and * indicates the significance of the difference. **(D)** Correlation coefficients between *CHMP7* expression levels and Ploidy or HRD. **(E)** Association between *CHMP7* expression and neoantigen counts. The waves at the top and right side represent the distribution density of *CHMP7* and neoantigen counts. (*: *p* < 0.05, **: *p* < 0.01 and ***: *p* < 0.001).

Mutation analysis of *CHMP7* in the TCGA database was performed on the cBioPortal website. The results reveal that the top five tumors with alterations are PRAD, OV, LIHC, BLCA, and COAD, with the main mutation type being deletion. The main loci of mutations are shown in Figure 4A. To further analyze the effect of *CHMP7* mutations on patient prognosis, COAD and LUSC patients in the *CHMP7*-altered group had poorer disease-free survival (DFS; Figure 4B).

TMB and MSI are important clinical biomarkers that can be effectively predicted for tumor treatment (Baretti and Le, 2018; Zhang et al., 2022). Radar plots present the correlation between *CHMP7* and TMB, and MSI in different tumor tissues (Figure 4C). *CHMP7* is positively correlated with TMB in five tumors (LGG, COAD, STES, STAD, and UCEC) and significantly negatively correlated with TMB in three tumors (LUAD, KIRP, and THCA). As for MSI, *CHMP7* is significantly positively correlated in CESC, COAD, ESCA, STES, STAD, UCEC, READ, and UVM and negatively correlated in PRAD, THCA, and DLBC.

Ploidy is tightly associated with chromosomal instability in tumor development (Müller et al., 2021). The bar chart demonstrates the correlation of *CHMP7* with ploidy, which is significantly negatively correlated in nine types of tumors (BRCA, STES, KIPAN, PRAD, UCEC, KIRC, LUSC, THYM, and BLCA). *CHMP7* is significantly negatively correlated with HRD in CESC, LUAD, COAD, BRCA, ESCA, STES, KIPAN, STAD, UCEC, KIRC, LUSC, READ, TGCT, SKCM, UCS, BLCA, and CHOL. It presents a positive correlation in LGG (Figure 4D). NEO is abundantly expressed in tumor cells with strong immunogenicity and tumor heterogeneity, making it an attractive target for tumor immunotherapy (Blass and Ott, 2021). Scatter plots indicate a significant positive correlation between *CHMP7* and NEO in COAD, LGG, UCEC, and CESC, while a negative correlation in BLCA and PCPG (Figure 4E). All analyses suggest that *CHMP7* is altered in various tumor tissues and associated with genomic instability.

### *CHMP7* is associated with gene repair in pan-cancerous tissues

**FIGURE 5:**
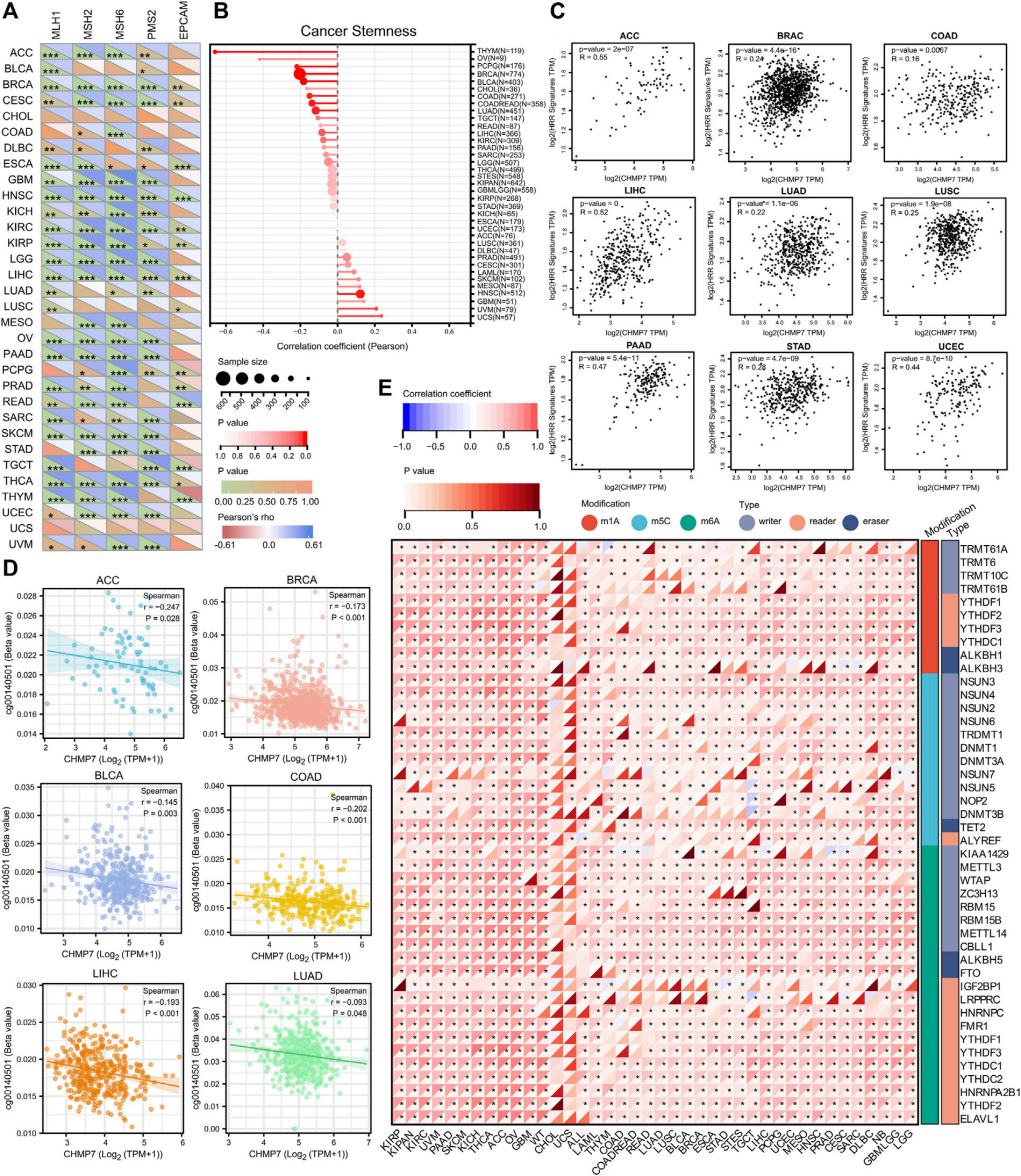
*CHMP7* is involved in DNA mismatch repair, cancer stemness and epigenetic regulation. **(A)** Heat map demonstrating the correlation between *CHMP7* and 5 MMR genes (*MLH1, MSH2, MSH6, PMS2, EPCAM*) in pan-cancer. **(B)** Lollipop plot reveals the association between *CHMP7* expression levels and cancer stemness. The dot size indicates the number of samples and the color indicates the *p*-value. **(C)** Scatter plot depicting the correlation between *CHMP7* expression levels and HRR characteristics. **(D)** *CHMP7* DNA methylation was significantly negatively correlated with probe cg00140501 in ACC, BRCA, BLCA, COAD, LIHC and LUAD. **(E)** The correlation between *CHMP7* expression levels and RNA regulation. (*: *p* < 0.05, **: *p* < 0.01 and ***: *p* < 0.001).

The stability of the genome relies on the combined action of multiple repair mechanisms, such as MMR and HRR. The diagonal heat map demonstrates the correlation between *CHMP7* and MMR-related genes (*MLH1, MSH2, MSH6, PMS2*, and *EPCAM*), exhibiting positive correlation in various tumor tissues, including ACC, BRCA, and KIRC (Figure 5A). The correlation between *CHMP7* and cancer stemness was analyzed based on six tumor stemness indices calculated by mRNA expression and methylation signature, which were significantly positively correlated in HNSC and negatively correlated in LUAD, COAD, COADREAD, BRCA, THYM, PCPG, and BLCA (Figure 5B; Supplementary Material S2). A positive correlation between *CHMP7* and HRR was found in multiple tumor tissues, indicating that *CHMP7* may be associated with DNA repair (Figure 5C).

DNAss is a cancer stemness score based on the methylation profile calculated for each tumor and further analyzed for *CHMP7* DNA methylation in the TCGA database (Malta et al., 2018). A significant negative correlation was observed between *CHMP7* and DNA methylation probe cg00140501 in several tumor tissues of TCGA, including ACC, BRCA, BLCA, COAD, LIHC, and LUAD (Figure 5D). In addition to methylation analysis, the correlation between *CHMP7* and RNA-modified genes was analyzed, revealing a heat map showing a positive correlation between *CHMP7* and major RNA-modified genes (Figure 5E).

### AS events in *CHMP7* can contribute to predicting patient prognosis

**FIGURE 6:**
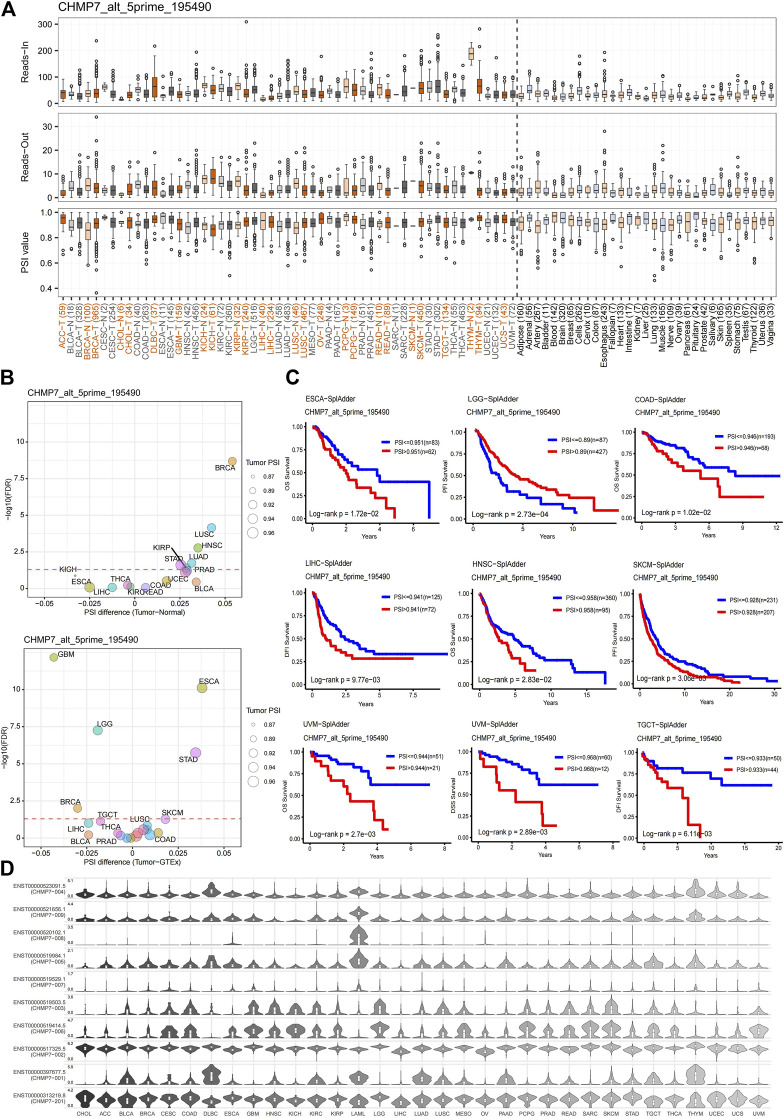
Alternative splicing analysis of *CHMP7*. **(A)** The read-in, read-out, and PSI values of *CHMP7* _alt_5prime in tumor and normal tissues. **(B)** Differences in PSI value between tumor and adjacent normal tissue (top), and tumor and GTEx normal tissue (bottom); red dashed line refers to FDR of 0.05. **(C)** Kaplan-Meier curves present the effect of PSI values of *CHMP7* _alt_5prime on patient OS, DSS, DFI, and PFI. **(D)** Isoform switch events of the *CHMP7* gene in pan-cancer.

AS is an essential mechanism for regulating gene expression and generating proteomic diversity, which has the potential to serve as a new biomarker in oncology and provide many new targets for drug development (Blencowe, 2006). The AS events for *CHMP7* were evaluated at the OncoSplicing website, and alt_5prime_195490 is presented in Figure 6A and the rest in Supplementary Material S3. A higher PSI was observed in BRCA, CHOL, KIRP, and READ tumors. The difference in PSI between tumor tissues and adjacent/normal tissues was compared, and *CHMP7*_alt_5prime_195490 exhibited higher PSI in various tumor tissues, such as BRCA and STAD (Figure 6B). The predictive value of PSI values on the prognosis of patients with tumors was analyzed. The results suggested that high PSI values were associated with poorer OS in ESCA, COAD, HNSC, and UVM and poorer DFI in LIHC, SKCM, and TGCT (Figure 6C). Shear isoforms of *CHMP7* in pan-cancer were also demonstrated (Figure 6D). All these results indicate that AS events of *CHMP7* are critical in tumor research.

### *CHMP7* is engaged in tumor immune infiltration and regulation

**FIGURE 7:**
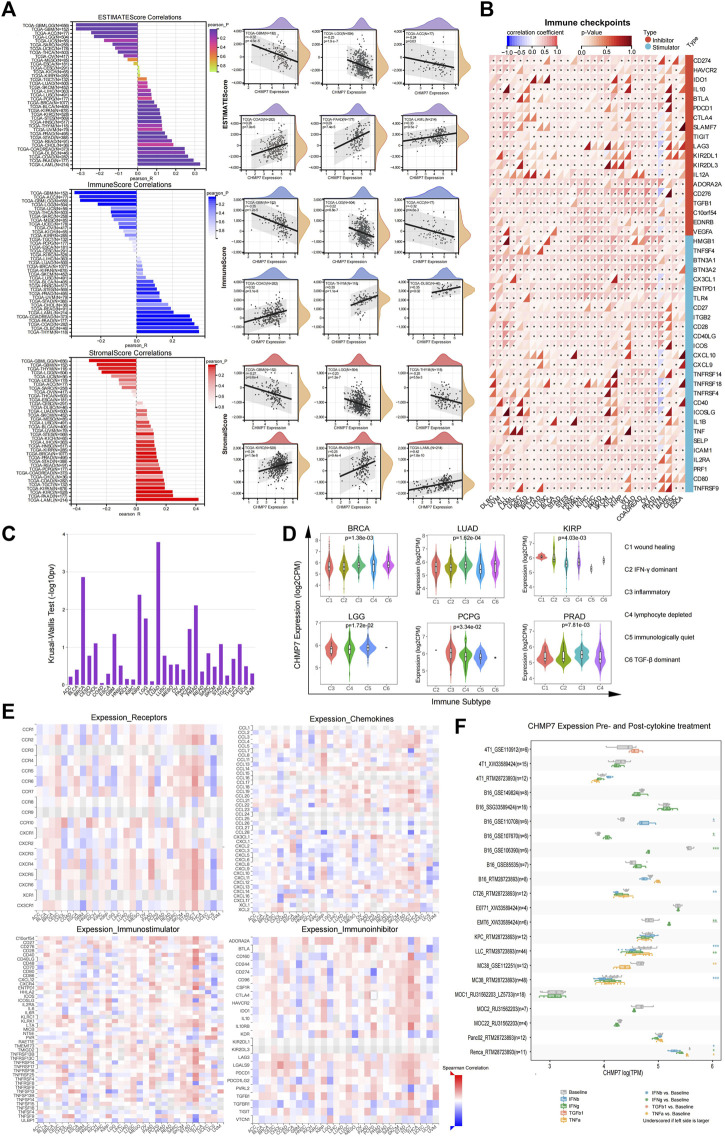
*CHMP7* is correlated with immune infiltrates and immune checkpoints. **(A)** Bar graph displaying the correlation between *CHMP7* and ESTIMATEScore, ImmuneScore and StromalScore immune infiltration scores, and scatter plot presenting the top 6 correlated cancers for each immune infiltration score. **(B)** Heat map of the association between immune checkpoint and *CHMP7* expression levels. **(C)** The association between *CHMP7* and immune subtypes was obtained by TSIDB online tool. **(D)** *CHMP7* expression levels were correlated with immune subtypes. **(E)** Heat map of correlation between *CHMP7* expression and receptors (top left), chemokines (bottom left), immunostimulatory factors (top right) immunosuppressive factors (bottom right). **(F)** Box line plot of changes in *CHMP7* expression levels in tumor cell lines before and after cytokine treatment. (*: *p* < 0.05, **: *p* < 0.01 and ***: *p* < 0.001).

To investigate the relationship between *CHMP7* and immune infiltration in the tumor TME, we calculated the stromal and immune scores of tumor samples based on *CHMP7* expression data using the ESTIMATE package of R software. The two scores were summed to obtain the ESTIMATE score, which can be used to estimate tumor purity. Significant correlation between *CHMP7* expression and immune infiltration was observed in 20 cancer species, 12 of which were significantly positively correlated, including TCGA-BRCA, TCGA-STES, TCGA-KIPAN, TCGA-COAD, TCGA-COADREAD, TCGA-PRAD, TCGA-STAD, TCGA-HNSC, TCGA -KIRC, TCGA-BLCA, TCGA-PAAD, and TCGA-LAML. There were eight significant negative correlations, such as TCGA-GBM, TCGA-GBMLGG, TCGA-LGG, TCGA-SARC, TARGET-WT, TCGA-THCA, TARGET-NB, and TCGA-ACC (Figure 7A). *CHMP7* was significantly positively correlated with most immune checkpoints and immunoregulatory genes in pan-cancerous tissues. Notably, *CHMP7* was negatively correlated with most immune checkpoints and immunoregulatory genes in THYM (Figure 7B; Supplementary Material S4).

The TISIDB enabled us to investigate the differential expression of *CHMP7* in different immune subtypes. The results revealed that *CHMP7* expression was significantly elevated in the C4 subtype in BRCA, KIRP, and LGG, indicating that *CHMP7* may be associated with lymphocyte function (Figures 7C, D). Heat maps demonstrate the correlation of *CHMP7* with cytokines, cytokine receptors, immunostimulatory factors, and immunosuppressive factors (Figure 7E). The TISMO website was utilized to compare the changes in *CHMP7* expression levels in tumor cell lines in both pre- and post-cytokine treatment (Figure 7F). *CHMP7* expression levels increased in several cell lines after IFN-β treatment, and the same was observed in several cell lines treated with IFN-γ. The results suggest that the downregulation of our *CHMP7* expression levels may lead to the suppression of immune checkpoint function, which is associated with suppressive TME.

### *CHMP7* is associated with M2 macrophage infiltration

**FIGURE 8:**
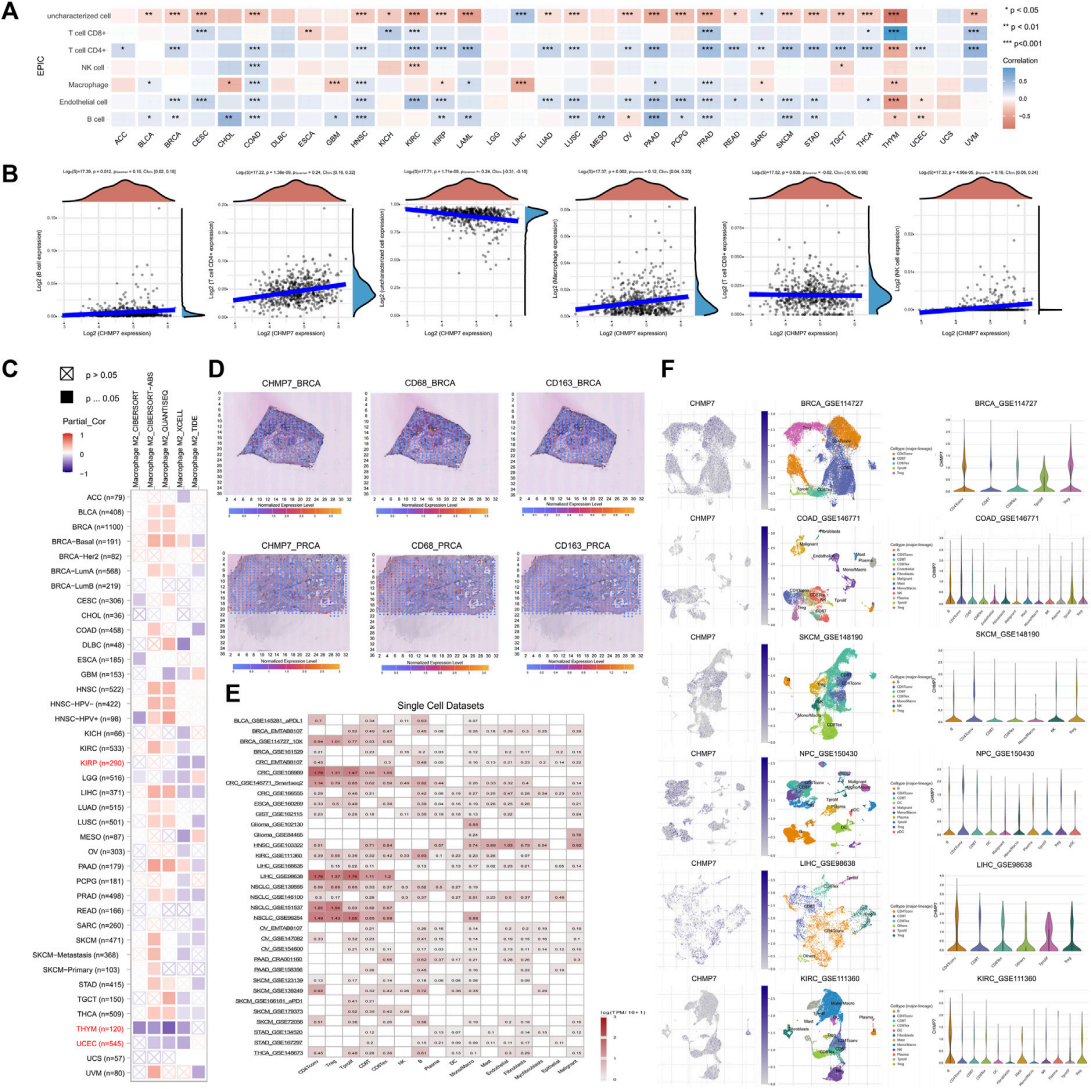
*CHMP7* is associated with tumor tissue infiltration of immune cells. **(A)** EPIC algorithm for calculating *CHMP7* correlation with immune cell infiltration. **(B)** Scatter plots representing *CHMP7* correlation with B cell, CD4^+^ T cell, CD8^+^ T cell, uncharacterized cell, macrophage and NK cell infiltration. **(C)** Correlation of *CHMP7* with M2 macrophage infiltration was calculated by TIMER2.0 with several algorithms. **(D)** Spatial transcriptional sections illustrated the spatial expression of *CHMP7* in BRCA and PRCA tissues and CD68 and CD163 markers. **(E)** Expression of *CHMP7* in cancer monocyte clusters correlates with immune cell infiltration. **(F)** Expression of *CHMP7* in different immune cells in single cell clusters of multiple tumor cells. (*: *p* < 0.05, **: *p* < 0.01 and ***: *p* < 0.001).

The relationship between *CHMP7* and immune cell infiltration in TME was analyzed using the EPIC algorithm (Sturm et al., 2019), and seven immune cells associated with *CHMP7* expression were obtained (Figure 8A). The results indicate that *CHMP7* negatively correlates with CD8^+^ T cells in CESC, KICH, KIRC, PRAD, THCA, THYM, and UVM. The negative correlation of *CHMP7* with tumor-associated macrophages has been observed in BLCA, COAD, HNSC, LAML, PAAD, and PRAD, while the opposite has been observed in CHOL, GBM, KIRP, LIHC, and THYM. The relationship between *CHMP7* and immune cells in COAD is shown in scatter plots (Figure 8B).

M2 macrophages can secrete suppressive cytokines such as TGF-β and IL-10, producing an immunosuppressive TME that promotes tumor progression (Genin et al., 2015). The TIMER2.0 website facilitated us to analyze the correlation between *CHMP7* and M2 macrophage infiltration levels with multiple algorithms, and consistent correlations were observed in KIRP, THYM, and UCEC, suggesting that downregulation of *CHMP7* expression may be associated with M2 macrophage infiltration (Figure 8C). The correlation between *CHMP7* and M2 macrophage markers (CD68, CD163) was analyzed at the spatial transcriptional level utilizing the SpatialDB, and *CHMP7* and M2 macrophage biomarkers presented a similar spatial distribution (Figure 8D).

Subsequently, we investigated the correlation between *CHMP7* and immune cell infiltration from the single-cell level. The relationship between *CHMP7* and immune cell infiltration was investigated with the help of the TISCH (Figure 8E). The correlation between *CHMP7* expression and immune cells in BRCA, COAD, SKCM, NPC, LIHC, and KIRC was analyzed, and there were differences in the expression levels of *CHMP7* in various infiltrating immune cells (Figure 8F).

### *CHMP7* is associated with CTL dysfunction

**FIGURE 9:**
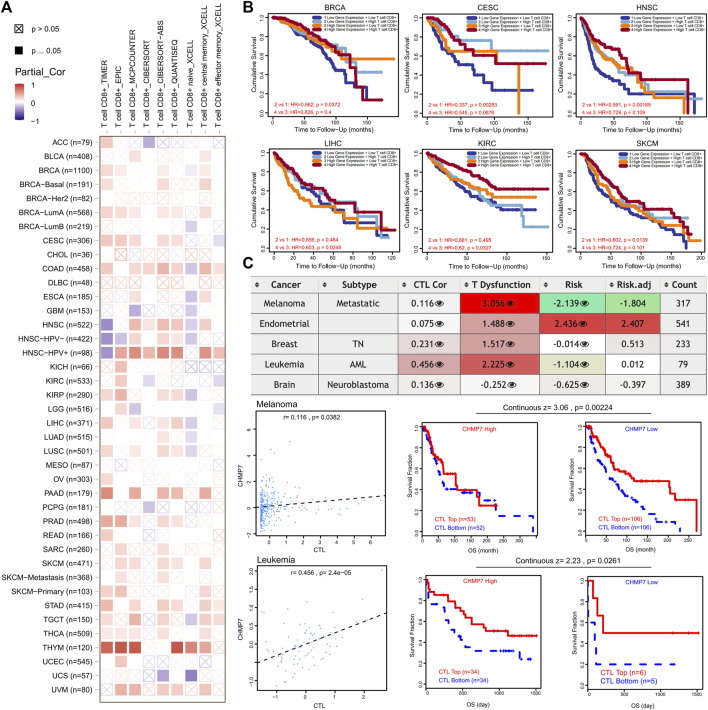
*CHMP7* has been positively correlated with CD8^+^ T-cell infiltration. **(A)** Heat map of association between *CHMP7* levels and CD8^+^ T-cell infiltration calculated by multiple algorithms. **(B)** Impact of *CHMP7* expression levels and CD8^+^ T-cell infiltration levels on prognosis of tumor patients. **(C)** Table depicting the correlation between *CHMP7* expression and CTL, CTL dysfunction and risk.

In addition to M2 macrophages, the relevance of *CHMP7* to CTL has also attracted our attention. Similar to M2 macrophages, we analyzed the correlation between *CHMP7* and CTL with multiple immune infiltration algorithms in TIMER2.0 and expressed consistent significant positive correlations in BLCA, BRCA-Basal, BRCA-LumA, PAAD, PRAD, SARC, SKCM, STAD, THCA, and UVM (Figure 9A). Combined *CHMP7* expression and CTL infiltration levels were analyzed for their predictive value for patient prognosis. The results reveal a poorer prognosis in the *CHMP7* low expression and CTL low infiltration groups in BRCA, CSC, HNSC, LIHC, KIRC, and SKCM (Figure 9B).

The correlation between *CHMP7* and CTL dysfunction was observed in SKCM, UCEC, BRCA, and LAML through the TIDE website. A significant positive correlation between *CHMP7* and CTL was demonstrated in SKCM and LAML (Figure 9C). The results suggest that *CHMP7* is associated with many immune cells, with the possible involvement of killing tumor cells by targeting CTLs.

### *CHMP7* may assist in predicting the efficacy of chemotherapy and immunotherapy

**FIGURE 10:**
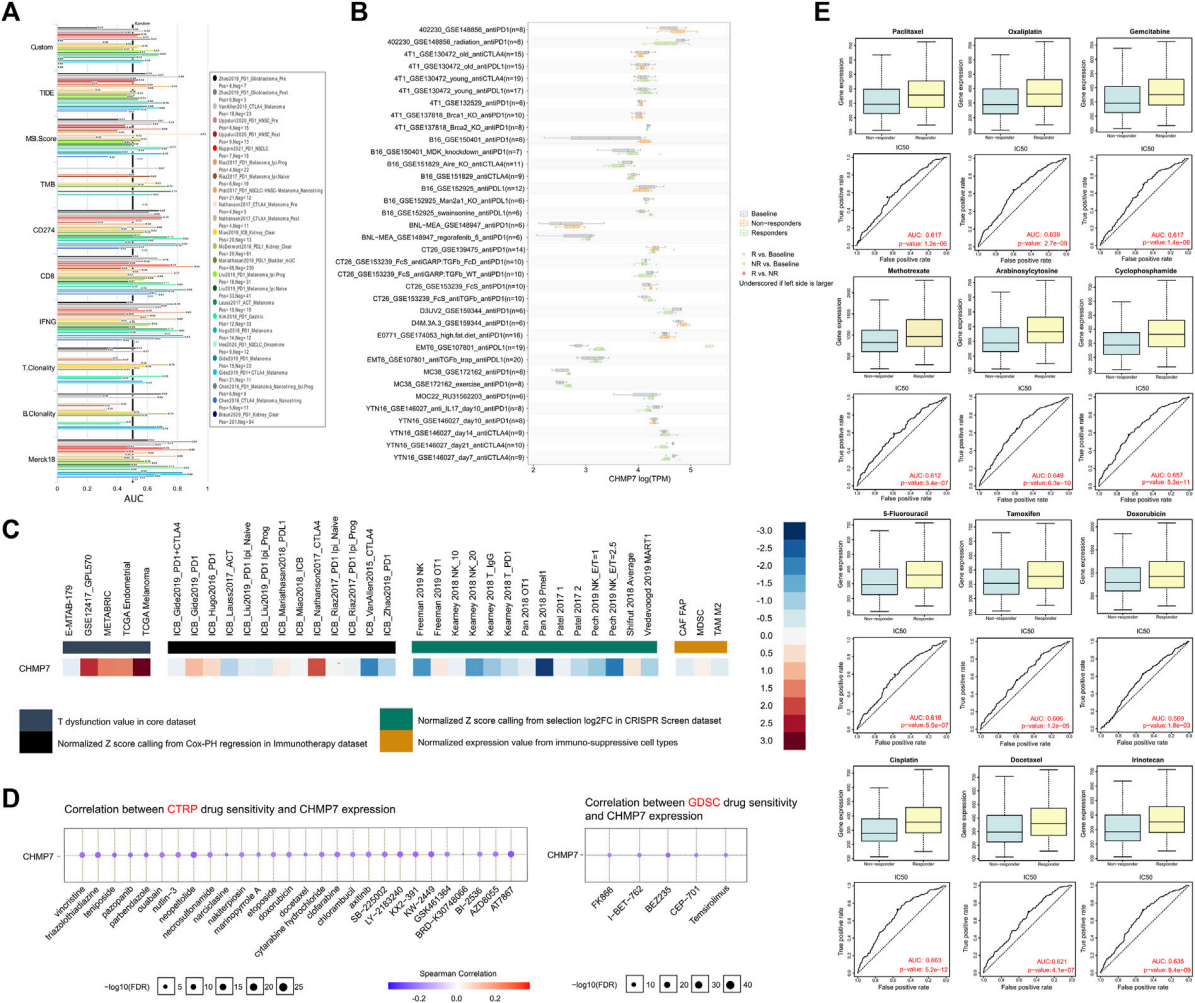
*CHMP7* may predict immunotherapy and chemotherapy drug sensitivity. **(A)** *CHMP7* correlates with immunotherapy response and **(B)** biomarkers in the immunotherapy cohort. **(C)** Therapeutic response of *CHMP7* in mechanistic follow-up experiments. **(D)** Prediction of *CHMP7* level-sensitive small compounds by CTRP and GDSC sites. **(E)** GDSC data-based analysis of differential *CHMP7* expression in multiple chemotherapy drug response and non-response groups. (*: *p* < 0.05, **: *p* < 0.01 and ***: *p* < 0.001).

To investigate the possible role of *CHMP7* in tumor therapy, we investigated the predictive value of *CHMP7* compared to classical biomarkers for immunotherapeutic response through the TIDE website (Figure 10A). The bar chart indicates that *CHMP7* presents an AUC above 0.5 in 11 of the 25 immunotherapy cohorts, suggesting a predictive significance. In 11 cohorts, the predictive value of *CHMP7* was superior to the MSI score of *CHMP7*. Higher predictive value of *CHMP7* was observed in 14, seven, 18, 18, 16, nine, and six immunotherapy cohorts compared with TIDE, TMB, CD274, CD8, IFN, T. Clonality, T. Clonality, and Merck18, respectively. It was observed in the antiPD-L1 treatment group of the EMT6 mouse breast cancer cell line that *CHMP7* expression was higher in the response group (Figure 10B). The TIDE website also assisted us in predicting the therapeutic response of *CHMP7* in the core dataset, immunotherapy dataset, CRISPR screening dataset, and mechanistic follow-up experiments with immunosuppressive cell types (Figure 10C).

In addition to the predictive value of *CHMP7* for immunotherapy efficacy, *CHMP7*-sensitive small molecule drugs were also analyzed using the CTRP and GDSC datasets (Figure 10D). It was further compared that *CHMP7* was differentially expressed in the responding and non-responding groups of multiple immunotherapy cohorts. The results revealed that *CHMP7* was differentially expressed in response and non-response groups in multiple immunotherapy cohorts including SRP094781, GSE67501, SRP230414, and IMvigor210 (Supplementary Material S5). The RNAact Drug website also facilitated our predictions (Supplementary Material S6). The results suggest that the top 5 sensitive drugs associated with CHMP7 expression based on the GDSC dataset are GSK429286A, KU-55933, BX-912, CCT007093, and Tretinoin (*p* < 0.05). Performing further exploration of the differential expression of *CHMP7* in the response and non-response groups of multiple chemotherapeutic agents, we discovered that high *CHMP7* expression may be associated with drug sensitivity to chemotherapeutic agents such as paclitaxel, oxaliplatin, gemcitabine, and methotrexate, which are now commonly recommended (Figure 10E).

## Discussion

Tumor progression is attributed to the accumulation of random mutations and epigenetic alterations in DNA sequences that affect the proliferation of malignant cells associated with gene regulatory networks and other traits associated with the malignant phenotype (Nakagawa and Fujita, 2018). ESCRT is a molecular machine that participates in various essential physiological processes, such as the formation of multivesicular bodies, involvement in cellular autophagy, and repair of cellular membranes. The dysregulation of ESCRT function is highly related to tumor development, and *CHMP7,* an important regulatory subunit of ESCRT-III, has attracted our attention. The dysregulated function of ESCRT may affect the proliferation and migration capacity of tumor cells through the sorting and delivery of exosomes (Wei et al., 2015). In this study, *CHMP7* has been comprehensively described with the help of public databases of tumor tissues. The differential expression of *CHMP7* in tumor and normal tissues was first compared, and the predictive value of *CHMP7* for patient OS was assessed further. The role of *CHMP7* in tumor immunity is a central focus of our study and excels as a biomarker for predicting the efficacy of tumor immunotherapy and chemotherapy. The low *CHMP7* expression group was associated with poor prognosis in BRCA, COAD, HNSC, and KIRC, and the level of *CHMP7* expression decreased with a more advanced tumor stage. The results suggest that normal *CHMP7* expression may be crucial for maintaining normal cellular function.

DNA integrity affected the accuracy of genetic information transmission in organisms, and the ESCRT system is thought to be related to cell division, where we further explored the correlation between *CHMP7* and the DNA damage repair response. To maintain genome integrity, complex DNA repair systems are employed to counteract various forms of DNA damage, and these mechanisms are known as the DNA damage response (Jackson and Bartek, 2009). Cellular DNA damage can be classified into single-strand break (SSB) and double-strand break (DSB). SSBs mainly rely on poly ADP-ribose polymerase (PARP) for nucleotide excision repair (NER), base excision repair (BER), and MMR; while DSBs are repaired by HRR, non-homologous end joining (NHEJ), and microhomology-mediated end joining (MMEJ) pathways (Li et al., 2016). The inhibitor targeting PARP can promote the recruitment of DNA repair effector molecules and structural remodeling of chromatin around DNA damage sites, which can selectively kill tumor cells with HRD. Therefore, it is a promising therapeutic strategy for BRCA-mutated tumors (Slade, 2020). In this study, *CHMP7* was significantly and positively correlated with MMR and HRR-related gene signatures in various tumor tissues. The results indicate that normal expression of *CHMP7* is essential for cells to complete DNA repair through MMR and HRR pathways. Furthermore, *CHMP7* was significantly and negatively correlated with Ploidy and HRD. The results show that the downregulation of *CHMP7* may lead to chromosomal instability from another aspect. All these findings prompt us that *CHMP7* may be intimately involved in the DNA repair process and deserves to be explored in depth.

The immune microenvironment and immunotherapy represent emerging trends in oncology research. In addition to tumor cells, TME includes infiltrating immune and inflammatory cells, CAFs, ECM, microvasculature, and various cytokines and chemokines (Bilotta et al., 2022). The heterogeneity of TME is inextricably linked to the different response rates of patients with tumors to immunotherapy. Low *CHMP7* expression was significantly correlated with immunosuppressive TME, as evidenced by high infiltration of M2 macrophages and reduced CTL and cytokines. The analysis of the correlation between *CHMP7* and immune cell infiltration levels shows that *CHMP7* and M2 macrophages are significantly negatively correlated, which has been demonstrated with multiple algorithms and spatial transcriptional data. Significant elevations in *CHMP7* expression were detected following multiple cytokine immunotherapy cohorts. As the immune cell that primarily kills tumor cells in TME, lower *CHMP7* expression significantly inhibits the function of CTL, further leading to tumor progression and poor patient prognosis. Furthermore, *CHMP7* was identified to perform superiorly as a biomarker for predicting the efficacy of immunotherapy and chemotherapy (Zheng et al., 2023). *CHMP7* was superior to traditional biomarkers in several immunotherapy cohorts, such as TMB and MSI scores. *CHMP7* levels were significantly lower in the non-responder group for several common chemotherapeutic agents. The results suggest that downregulated *CHMP7* levels may lead to the occurrence of tumor drug resistance.

## Conclusion

In this article, we utilized public databases such as TCGA, GTEx, TARGET, and single-cell sequencing data to provide a comprehensive and intensive analysis of the role of *CHMP7* in tumor development and therapy. *CHMP7* shows a predictive value for the prognosis of patients with tumors and is highly involved in tumor immunity. A strong correlation between *CHMP7* and TME immune cell infiltration has been observed, which is involved in the formation of suppressive TME and promotes tumor progression. *CHMP7* can potentially serve as a new biomarker for predicting the efficacy of chemotherapy and immunotherapy for tumors. As a gene of interest, *CHMP7* is expected to provide novel and promising targets for further treatment of patients with tumors.

## Data Availability

The original contributions presented in the study are included in the article/Supplementary Material, further inquiries can be directed to the corresponding author.

## Supplementary material

SUPPLEMENTARY MATERIAL S1 Correlation of *CHMP7* expression levels with cancer stemness scores. **(A)** Cancer stemness index based on epigenetic regulation of DNA methylation signatures. **(B)** Cancer stemness index based on differential methylation probes. **(C)** Cancer stemness index based on epigenetically regulated RNA expression. **(D)** Cancer stemness index based on enhancer elements/DNA methylation. **(E)** Cancer stemness index based on RNA expression.

SUPPLEMENTARY MATERIAL S2 AS events occurring with *CHMP7* in pan-cancerous tissues. **(A)** The read-in, read-out, and PSI values of *CHMP7* _alt_5prime_195490 in tumor and normal tissues. Differences in PSI value between tumor and adjacent normal tissue (left), and tumor and GTEx normal tissue (right); red dashed line refers to FDR of 0.05. **(B)** The read-in, read-out, and PSI values of *CHMP7*_exon_skip_482248 in tumor and normal tissues. Differences in PSI value between tumor and adjacent normal tissue (left), and tumor and GTEx normal tissue (right); red dashed line refers to FDR of 0.05. **(C)** PSI values of *CHMP7*_AD_83076 in tumor and normal tissues. **(D)** PSI values of *CHMP7*_ES_83072 in tumor and normal tissues. **(E)** PSI values of *CHMP7*_ES_83073 in tumor and normal tissues. **(F)** Differences in PSI value of *CHMP7*_AD_83076, *CHMP7*_ES_83072 and *CHMP7*_ES_83073 between tumor and adjacent normal tissue respectively. (AD, alternate donor site; ES, exon skipping).

SUPPLEMENTARY MATERIAL S3 Top 100 genes associated with *CHMP7* obtained from the GEPIA website.

SUPPLEMENTARY MATERIAL S4 Correlation of *CHMP7* expression levels and immune regulatory genes.

SUPPLEMENTARY MATERIAL S5
*CHMP7* expression for Response and Non-response based on Pre-treatment Samples in each dataset.

SUPPLEMENTARY MATERIAL S6 Small molecule drugs related to *CHMP7* as predicted by the RNAact Drug website.

## Abbreviations

AS, alternative splicing; CAFs, cancer associated fibroblasts; CHMP7, charged multivesicular body protein 7; CTLs, cytotoxic T lymphocytes; ECM, extracellular matrix; TME, tumor microenvironment; DFS, disease-free survival; EGFR, epidermal growth factor receptor; ESCRT, endosomal sorting complex required for transport; EVs, extracellular vehicles; GEO, Gene Expression Omnibus database; GSEA, gene set enrichment analysis; HRD, homologous recombination deficiency; HRR, homologous recombination repair; ILVs, intraluminal vesicles; MSI, microsatellite instability; NEO, neoantigen; OS, overall survival; PPI, protein-protein interaction; PSI, percent spliced-in; TARGET, Therapeutically Applicable Research to Generate Effective Treatments; TCGA, the Cancer Genome Atlas; TMB, tumor mutation burden.
