# Supplementary material for: Identification of CHMP7 as a promising immunobiomarker for immunotherapy and chemotherapy and impact on prognosis of colorectal cancer patients
Source: Front Cell Dev Biol. 2023 Aug 30;11:1211843. doi: 10.3389/fcell.2023.1211843 (PMC10499328; doi:10.3389/fcell.2023.1211843)
Supplement: Supplementary file 2 [file DataSheet1.ZIP › Fig2G.R]

library(survival)library(survminer)library(ggplot2)head(data)#   event time                    group# 1     0   56 High expression of CHMP7# 2     0   63 High expression of CHMP7# 3     0   45 High expression of CHMP7# 4     0   53 High expression of CHMP7# 5     0   52 High expression of CHMP7# 6     0   55 High expression of CHMP7fit <- survfit(Surv(time, event) ~ group, data = data)print(fit)# Call: survfit(formula = survival::Surv(time, event) ~ group, data = dat)# #                                 n events median 0.95LCL 0.95UCL# group=High expression of CHMP7 25      1     NA      NA      NA# group=Low expression of CHMP7  25      8     60      58      NA# coxphfit_cox <- coxph(Surv(time, event) ~ group, data = data)print(fit_cox)# Call:# survival::coxph(formula = survival::Surv(time, event) ~ group, #     data = dat)# #   n= 50, number of events= 9 # #                               coef exp(coef) se(coef)     z Pr(>|z|)  # groupLow expression of CHMP7 2.182     8.863    1.061 2.056   0.0398 *# ---# Signif. codes:  0 ‘***’ 0.001 ‘**’ 0.01 ‘*’ 0.05 ‘.’ 0.1 ‘ ’ 1# #                              exp(coef) exp(-coef) lower .95 upper .95# groupLow expression of CHMP7     8.863     0.1128     1.107     70.96# # Concordance= 0.708  (se = 0.066 )# Likelihood ratio test= 6.92  on 1 df,   p=0.009# Wald test            = 4.23  on 1 df,   p=0.04# Score (logrank) test = 6.18  on 1 df,   p=0.01# cox.zph(fit_cox)#        chisq df    p# group  0.124  1 0.72# GLOBAL 0.124  1 0.72## plotggsurvplot(fit = fit, data = data, fun = "pct",           palette = c("#0073C2", "#EFC000", "#868686", "#CD534C", "#7AA6DC"),           linetype = 1, pval = TRUE,            censor = TRUE, censor.size = 7,           risk.table = TRUE, conf.int = FALSE)
