# Supplementary material for: Identification of CHMP7 as a promising immunobiomarker for immunotherapy and chemotherapy and impact on prognosis of colorectal cancer patients
Source: Front Cell Dev Biol. 2023 Aug 30;11:1211843. doi: 10.3389/fcell.2023.1211843 (PMC10499328; doi:10.3389/fcell.2023.1211843)
Supplement: Supplementary file 2 [file DataSheet1.ZIP › Fig2E-BRCA-OS.R]

library(survival)library(survminer)library(ggplot2)head(data)#   event time    value group# 1     0 4047 6.683451  High# 2     0 4005 6.436815  High# 3     0 1474 4.606484   Low# 4     0 1448 5.353436  High# 5     0  348 5.190453   Low# 6     0 1477 5.632917  Highfit <- survfit(Surv(time, event) ~ group, data = data)print(fit)# Call: survfit(formula = survival::Surv(time, event) ~ group, data = dat)# #              n events median 0.95LCL 0.95UCL# group=Low  542     92   3873    3462      NA# group=High 544     60   3941    3492      NA# coxphfit_cox <- coxph(Surv(time, event) ~ group, data = data)print(fit_cox)# Call:# survival::coxph(formula = survival::Surv(time, event) ~ group, #     data = dat)# #   n= 1086, number of events= 152 # #              coef exp(coef) se(coef)      z Pr(>|z|)  # groupHigh -0.3705    0.6904   0.1664 -2.227    0.026 *# ---# Signif. codes:  0 ‘***’ 0.001 ‘**’ 0.01 ‘*’ 0.05 ‘.’ 0.1 ‘ ’ 1# #           exp(coef) exp(-coef) lower .95 upper .95# groupHigh    0.6904      1.448    0.4983    0.9566# # Concordance= 0.568  (se = 0.024 )# Likelihood ratio test= 5.06  on 1 df,   p=0.02# Wald test            = 4.96  on 1 df,   p=0.03# Score (logrank) test = 5.01  on 1 df,   p=0.03# cox.zph(fit_cox)#        chisq df    p# group   2.08  1 0.15# GLOBAL  2.08  1 0.15## plotggsurvplot(fit = fit, data = data, fun = "pct",           palette = c("#0073C2", "#EFC000", "#868686", "#CD534C", "#7AA6DC"),           linetype = 1, pval = TRUE,            censor = TRUE, censor.size = 7,           risk.table = FALSE, conf.int = FALSE)
