# Supplementary material for: Identification of CHMP7 as a promising immunobiomarker for immunotherapy and chemotherapy and impact on prognosis of colorectal cancer patients
Source: Front Cell Dev Biol. 2023 Aug 30;11:1211843. doi: 10.3389/fcell.2023.1211843 (PMC10499328; doi:10.3389/fcell.2023.1211843)
Supplement: Supplementary file 2 [file DataSheet1.ZIP › Fig2E-ESCA-OS.R]

library(survival)library(survminer)library(ggplot2)head(data)#   event time    value group# 1     1  784 4.789922  High# 2     1  610 3.441643   Low# 3     1  951 4.429087   Low# 4     1  435 4.332636   Low# 5     1 1781 4.974534  High# 6     1  232 4.729950  Highfit <- survfit(Surv(time, event) ~ group, data = data)print(fit)# Call: survfit(formula = survival::Surv(time, event) ~ group, data = dat)# #             n events median 0.95LCL 0.95UCL# group=Low  81     39    730     557    1263# group=High 82     27   1361     784      NA# coxphfit_cox <- coxph(Surv(time, event) ~ group, data = data)print(fit_cox)# Call:# survival::coxph(formula = survival::Surv(time, event) ~ group, #     data = dat)# #   n= 163, number of events= 66 # #              coef exp(coef) se(coef)      z Pr(>|z|)  # groupHigh -0.5261    0.5909   0.2533 -2.077   0.0378 *# ---# Signif. codes:  0 ‘***’ 0.001 ‘**’ 0.01 ‘*’ 0.05 ‘.’ 0.1 ‘ ’ 1# #           exp(coef) exp(-coef) lower .95 upper .95# groupHigh    0.5909      1.692    0.3597    0.9707# # Concordance= 0.554  (se = 0.035 )# Likelihood ratio test= 4.4  on 1 df,   p=0.04# Wald test            = 4.32  on 1 df,   p=0.04# Score (logrank) test = 4.41  on 1 df,   p=0.04# cox.zph(fit_cox)#        chisq df    p# group  0.295  1 0.59# GLOBAL 0.295  1 0.59## plotggsurvplot(fit = fit, data = data, fun = "pct",           palette = c("#0073C2", "#EFC000", "#868686", "#CD534C", "#7AA6DC"),           linetype = 1, pval = TRUE,            censor = TRUE, censor.size = 7,           risk.table = FALSE, conf.int = FALSE)
