# Supplementary material for: Identification of CHMP7 as a promising immunobiomarker for immunotherapy and chemotherapy and impact on prognosis of colorectal cancer patients
Source: Front Cell Dev Biol. 2023 Aug 30;11:1211843. doi: 10.3389/fcell.2023.1211843 (PMC10499328; doi:10.3389/fcell.2023.1211843)
Supplement: Supplementary file 2 [file DataSheet1.ZIP › Fig2E-GBMLGG-OS.R]

library(survival)library(survminer)library(ggplot2)head(data)#   event time    value group# 1     1  448 5.317601   Low# 2     1   76 5.503854  High# 3     0  466 5.888958  High# 4     0  470 5.573487  High# 5     1  618 4.622286   Low# 6     1 1448 5.544708  Highfit <- survfit(Surv(time, event) ~ group, data = data)print(fit)# Call: survfit(formula = survival::Surv(time, event) ~ group, data = dat)# #              n events median 0.95LCL 0.95UCL# group=Low  349    139   1426    1062    1891# group=High 349    133   1525    1335    2379# coxphfit_cox <- coxph(Surv(time, event) ~ group, data = data)print(fit_cox)# Call:# survival::coxph(formula = survival::Surv(time, event) ~ group, #     data = dat)# #   n= 698, number of events= 272 # #              coef exp(coef) se(coef)      z Pr(>|z|)  # groupHigh -0.2225    0.8005   0.1218 -1.827   0.0677 .# ---# Signif. codes:  0 ‘***’ 0.001 ‘**’ 0.01 ‘*’ 0.05 ‘.’ 0.1 ‘ ’ 1# #           exp(coef) exp(-coef) lower .95 upper .95# groupHigh    0.8005      1.249    0.6306     1.016# # Concordance= 0.535  (se = 0.017 )# Likelihood ratio test= 3.34  on 1 df,   p=0.07# Wald test            = 3.34  on 1 df,   p=0.07# Score (logrank) test = 3.35  on 1 df,   p=0.07# cox.zph(fit_cox)#        chisq df    p# group   0.34  1 0.56# GLOBAL  0.34  1 0.56## plotggsurvplot(fit = fit, data = data, fun = "pct",           palette = c("#0073C2", "#EFC000", "#868686", "#CD534C", "#7AA6DC"),           linetype = 1, pval = TRUE,            censor = TRUE, censor.size = 7,           risk.table = FALSE, conf.int = FALSE)
