# Supplementary material for: Identification of CHMP7 as a promising immunobiomarker for immunotherapy and chemotherapy and impact on prognosis of colorectal cancer patients
Source: Front Cell Dev Biol. 2023 Aug 30;11:1211843. doi: 10.3389/fcell.2023.1211843 (PMC10499328; doi:10.3389/fcell.2023.1211843)
Supplement: Supplementary file 2 [file DataSheet1.ZIP › Fig2E-HNSC-OS.R]

library(survival)library(survminer)library(ggplot2)head(data)#   event time    value group# 1     0  102 4.455807   Low# 2     1  462 4.160936   Low# 3     1  283 4.605583   Low# 4     1  415 3.899756   Low# 5     1 1134 5.091721  High# 6     1  276 5.624063  Highfit <- survfit(Surv(time, event) ~ group, data = data)print(fit)# Call: survfit(formula = survival::Surv(time, event) ~ group, data = dat)# #              n events median 0.95LCL 0.95UCL# group=Low  251    119   1289     882    1718# group=High 252     99   2064    1732    2703# coxphfit_cox <- coxph(Surv(time, event) ~ group, data = data)print(fit_cox)# Call:# survival::coxph(formula = survival::Surv(time, event) ~ group, #     data = dat)# #   n= 503, number of events= 218 # #              coef exp(coef) se(coef)      z Pr(>|z|)  # groupHigh -0.2959    0.7439   0.1371 -2.158    0.031 *# ---# Signif. codes:  0 ‘***’ 0.001 ‘**’ 0.01 ‘*’ 0.05 ‘.’ 0.1 ‘ ’ 1# #           exp(coef) exp(-coef) lower .95 upper .95# groupHigh    0.7439      1.344    0.5686    0.9732# # Concordance= 0.548  (se = 0.019 )# Likelihood ratio test= 4.68  on 1 df,   p=0.03# Wald test            = 4.66  on 1 df,   p=0.03# Score (logrank) test = 4.69  on 1 df,   p=0.03# cox.zph(fit_cox)#        chisq df    p# group   2.35  1 0.12# GLOBAL  2.35  1 0.12## plotggsurvplot(fit = fit, data = data, fun = "pct",           palette = c("#0073C2", "#EFC000", "#868686", "#CD534C", "#7AA6DC"),           linetype = 1, pval = TRUE,            censor = TRUE, censor.size = 7,           risk.table = FALSE, conf.int = FALSE)
