# Supplementary material for: Identification of CHMP7 as a promising immunobiomarker for immunotherapy and chemotherapy and impact on prognosis of colorectal cancer patients
Source: Front Cell Dev Biol. 2023 Aug 30;11:1211843. doi: 10.3389/fcell.2023.1211843 (PMC10499328; doi:10.3389/fcell.2023.1211843)
Supplement: Supplementary file 2 [file DataSheet1.ZIP › Fig2E-KIRC-OS.R]

library(survival)library(survminer)library(ggplot2)head(data)#   event time    value group# 1     0  385 5.411962  High# 2     0  362 5.228111  High# 3     0 1120 5.050058  High# 4     0 1436 5.511971  High# 5     0   16 5.568823  High# 6     1 1191 5.127216  Highfit <- survfit(Surv(time, event) ~ group, data = data)print(fit)# Call: survfit(formula = survival::Surv(time, event) ~ group, data = dat)# #              n events median 0.95LCL 0.95UCL# group=Low  270    106   2190    1912      NA# group=High 271     69     NA    2830      NA# coxphfit_cox <- coxph(Surv(time, event) ~ group, data = data)print(fit_cox)# Call:# survival::coxph(formula = survival::Surv(time, event) ~ group, #     data = dat)# #   n= 541, number of events= 175 # #              coef exp(coef) se(coef)      z Pr(>|z|)   # groupHigh -0.4715    0.6241   0.1550 -3.043  0.00235 **# ---# Signif. codes:  0 ‘***’ 0.001 ‘**’ 0.01 ‘*’ 0.05 ‘.’ 0.1 ‘ ’ 1# #           exp(coef) exp(-coef) lower .95 upper .95# groupHigh    0.6241      1.602    0.4606    0.8455# # Concordance= 0.546  (se = 0.021 )# Likelihood ratio test= 9.49  on 1 df,   p=0.002# Wald test            = 9.26  on 1 df,   p=0.002# Score (logrank) test = 9.43  on 1 df,   p=0.002# cox.zph(fit_cox)#        chisq df    p# group   2.49  1 0.11# GLOBAL  2.49  1 0.11## plotggsurvplot(fit = fit, data = data, fun = "pct",           palette = c("#0073C2", "#EFC000", "#868686", "#CD534C", "#7AA6DC"),           linetype = 1, pval = TRUE,            censor = TRUE, censor.size = 7,           risk.table = FALSE, conf.int = FALSE)
