# Supplementary material for: Identification of CHMP7 as a promising immunobiomarker for immunotherapy and chemotherapy and impact on prognosis of colorectal cancer patients
Source: Front Cell Dev Biol. 2023 Aug 30;11:1211843. doi: 10.3389/fcell.2023.1211843 (PMC10499328; doi:10.3389/fcell.2023.1211843)
Supplement: Supplementary file 2 [file DataSheet1.ZIP › Fig2E-LAML-OS.R]

library(survival)library(survminer)library(ggplot2)head(data)#   event time    value group# 1     1  577 5.340285  High# 2     1  945 5.015997  High# 3     0 2861 4.719441   Low# 4     1   31 3.456622   Low# 5     1  243 4.069212   Low# 6     1  366 3.968266   Lowfit <- survfit(Surv(time, event) ~ group, data = data)print(fit)# Call: survfit(formula = survival::Surv(time, event) ~ group, data = dat)# #             n events median 0.95LCL 0.95UCL# group=Low  71     42    822     489    1642# group=High 68     45    304     245     580# coxphfit_cox <- coxph(Surv(time, event) ~ group, data = data)print(fit_cox)# Call:# survival::coxph(formula = survival::Surv(time, event) ~ group, #     data = dat)# #   n= 139, number of events= 87 # #             coef exp(coef) se(coef)     z Pr(>|z|)   # groupHigh 0.5675    1.7639   0.2188 2.593  0.00951 **# ---# Signif. codes:  0 ‘***’ 0.001 ‘**’ 0.01 ‘*’ 0.05 ‘.’ 0.1 ‘ ’ 1# #           exp(coef) exp(-coef) lower .95 upper .95# groupHigh     1.764     0.5669     1.149     2.709# # Concordance= 0.57  (se = 0.029 )# Likelihood ratio test= 6.7  on 1 df,   p=0.01# Wald test            = 6.72  on 1 df,   p=0.01# Score (logrank) test = 6.89  on 1 df,   p=0.009# cox.zph(fit_cox)#         chisq df    p# group  0.0131  1 0.91# GLOBAL 0.0131  1 0.91## plotggsurvplot(fit = fit, data = data, fun = "pct",           palette = c("#0073C2", "#EFC000", "#868686", "#CD534C", "#7AA6DC"),           linetype = 1, pval = TRUE,            censor = TRUE, censor.size = 7,           risk.table = FALSE, conf.int = FALSE)
