# Supplementary material for: Identification of CHMP7 as a promising immunobiomarker for immunotherapy and chemotherapy and impact on prognosis of colorectal cancer patients
Source: Front Cell Dev Biol. 2023 Aug 30;11:1211843. doi: 10.3389/fcell.2023.1211843 (PMC10499328; doi:10.3389/fcell.2023.1211843)
Supplement: Supplementary file 2 [file DataSheet1.ZIP › Fig2E-SARC-OS.R]

library(survival)library(survminer)library(ggplot2)head(data)#   event time    value group# 1     0 1521 6.091746  High# 2     0 1104 6.323136  High# 3     1  599 5.214160   Low# 4     0  959 5.095287   Low# 5     1 1627 5.792965  High# 6     0 2085 5.963869  Highfit <- survfit(Surv(time, event) ~ group, data = data)print(fit)# Call: survfit(formula = survival::Surv(time, event) ~ group, data = dat)# #              n events median 0.95LCL 0.95UCL# group=Low  131     50   2034    1235      NA# group=High 132     49   2324    1722      NA# coxphfit_cox <- coxph(Surv(time, event) ~ group, data = data)print(fit_cox)# Call:# survival::coxph(formula = survival::Surv(time, event) ~ group, #     data = dat)# #   n= 263, number of events= 99 # #              coef exp(coef) se(coef)      z Pr(>|z|)# groupHigh -0.1643    0.8485   0.2017 -0.814    0.415# #           exp(coef) exp(-coef) lower .95 upper .95# groupHigh    0.8485      1.179    0.5714      1.26# # Concordance= 0.527  (se = 0.028 )# Likelihood ratio test= 0.66  on 1 df,   p=0.4# Wald test            = 0.66  on 1 df,   p=0.4# Score (logrank) test = 0.66  on 1 df,   p=0.4# cox.zph(fit_cox)#        chisq df    p# group  0.372  1 0.54# GLOBAL 0.372  1 0.54## plotggsurvplot(fit = fit, data = data, fun = "pct",           palette = c("#0073C2", "#EFC000", "#868686", "#CD534C", "#7AA6DC"),           linetype = 1, pval = TRUE,            censor = TRUE, censor.size = 7,           risk.table = FALSE, conf.int = FALSE)
