# Supplementary material for: Identification of CHMP7 as a promising immunobiomarker for immunotherapy and chemotherapy and impact on prognosis of colorectal cancer patients
Source: Front Cell Dev Biol. 2023 Aug 30;11:1211843. doi: 10.3389/fcell.2023.1211843 (PMC10499328; doi:10.3389/fcell.2023.1211843)
Supplement: Supplementary file 2 [file DataSheet1.ZIP › Fig2E-SKCM-OS.R]

library(survival)library(survminer)library(ggplot2)head(data)#   event time    value group# 1     1  518 4.074052  High# 2     0 2022 3.395831   Low# 3     1  395 3.775788   Low# 4     0  387 3.970053  High# 5     0   14 4.885213  High# 6     1  282 3.818309   Lowfit <- survfit(Surv(time, event) ~ group, data = data)print(fit)# Call: survfit(formula = survival::Surv(time, event) ~ group, data = dat)# #              n events median 0.95LCL 0.95UCL# group=Low  226    114   2028    1524    2711# group=High 231    101   3139    2004    4634# coxphfit_cox <- coxph(Surv(time, event) ~ group, data = data)print(fit_cox)# Call:# survival::coxph(formula = survival::Surv(time, event) ~ group, #     data = dat)# #   n= 457, number of events= 215 # #              coef exp(coef) se(coef)     z Pr(>|z|)  # groupHigh -0.2938    0.7455   0.1379 -2.13   0.0332 *# ---# Signif. codes:  0 ‘***’ 0.001 ‘**’ 0.01 ‘*’ 0.05 ‘.’ 0.1 ‘ ’ 1# #           exp(coef) exp(-coef) lower .95 upper .95# groupHigh    0.7455      1.341    0.5689    0.9769# # Concordance= 0.551  (se = 0.019 )# Likelihood ratio test= 4.54  on 1 df,   p=0.03# Wald test            = 4.54  on 1 df,   p=0.03# Score (logrank) test = 4.57  on 1 df,   p=0.03# cox.zph(fit_cox)#        chisq df    p# group    2.4  1 0.12# GLOBAL   2.4  1 0.12## plotggsurvplot(fit = fit, data = data, fun = "pct",           palette = c("#0073C2", "#EFC000", "#868686", "#CD534C", "#7AA6DC"),           linetype = 1, pval = TRUE,            censor = TRUE, censor.size = 7,           risk.table = FALSE, conf.int = FALSE)
