# Supplementary material for: Identification of CHMP7 as a promising immunobiomarker for immunotherapy and chemotherapy and impact on prognosis of colorectal cancer patients
Source: Front Cell Dev Biol. 2023 Aug 30;11:1211843. doi: 10.3389/fcell.2023.1211843 (PMC10499328; doi:10.3389/fcell.2023.1211843)

## 575646-16 CHMP7


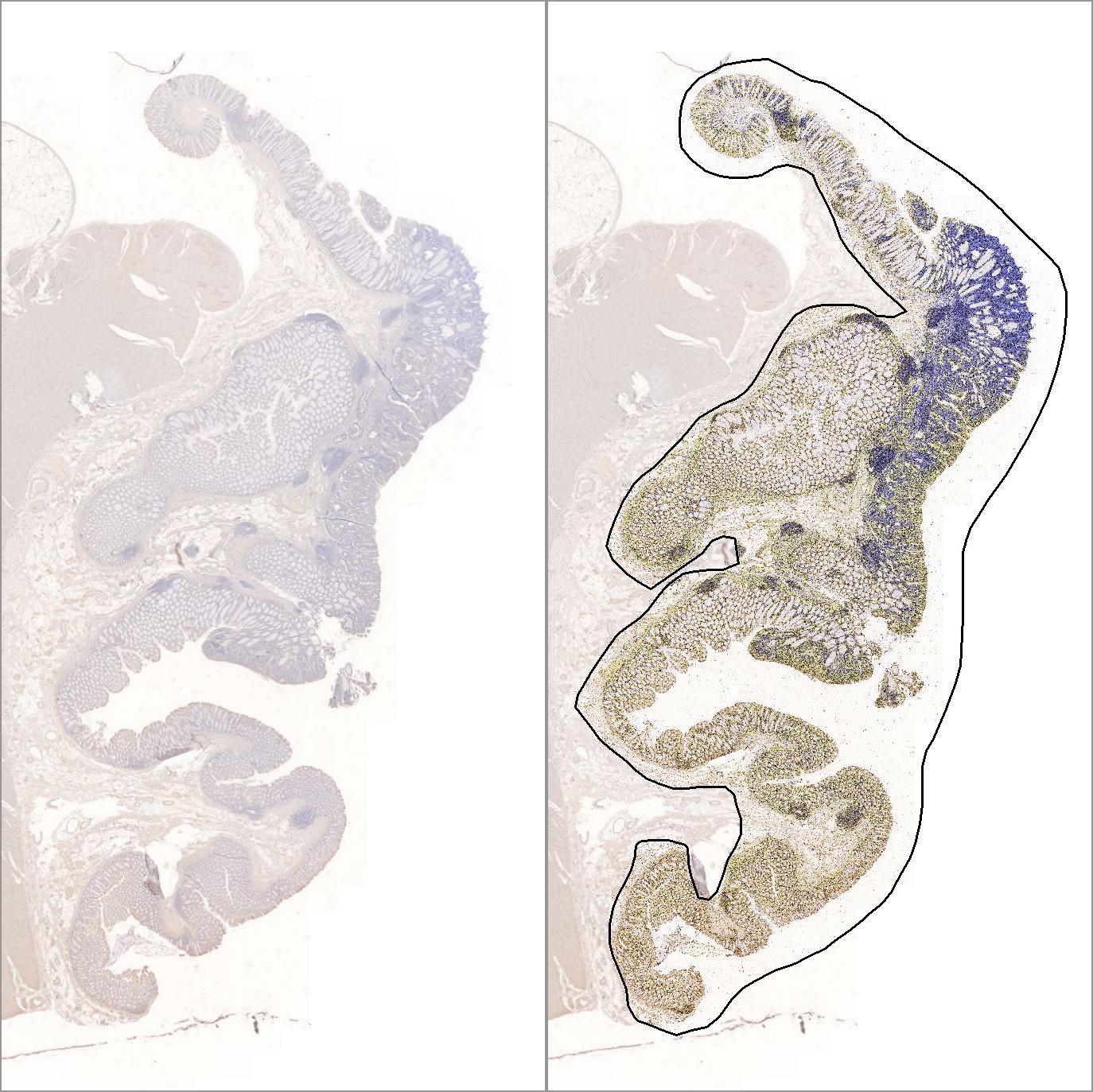


## 602830-1 CHMP7


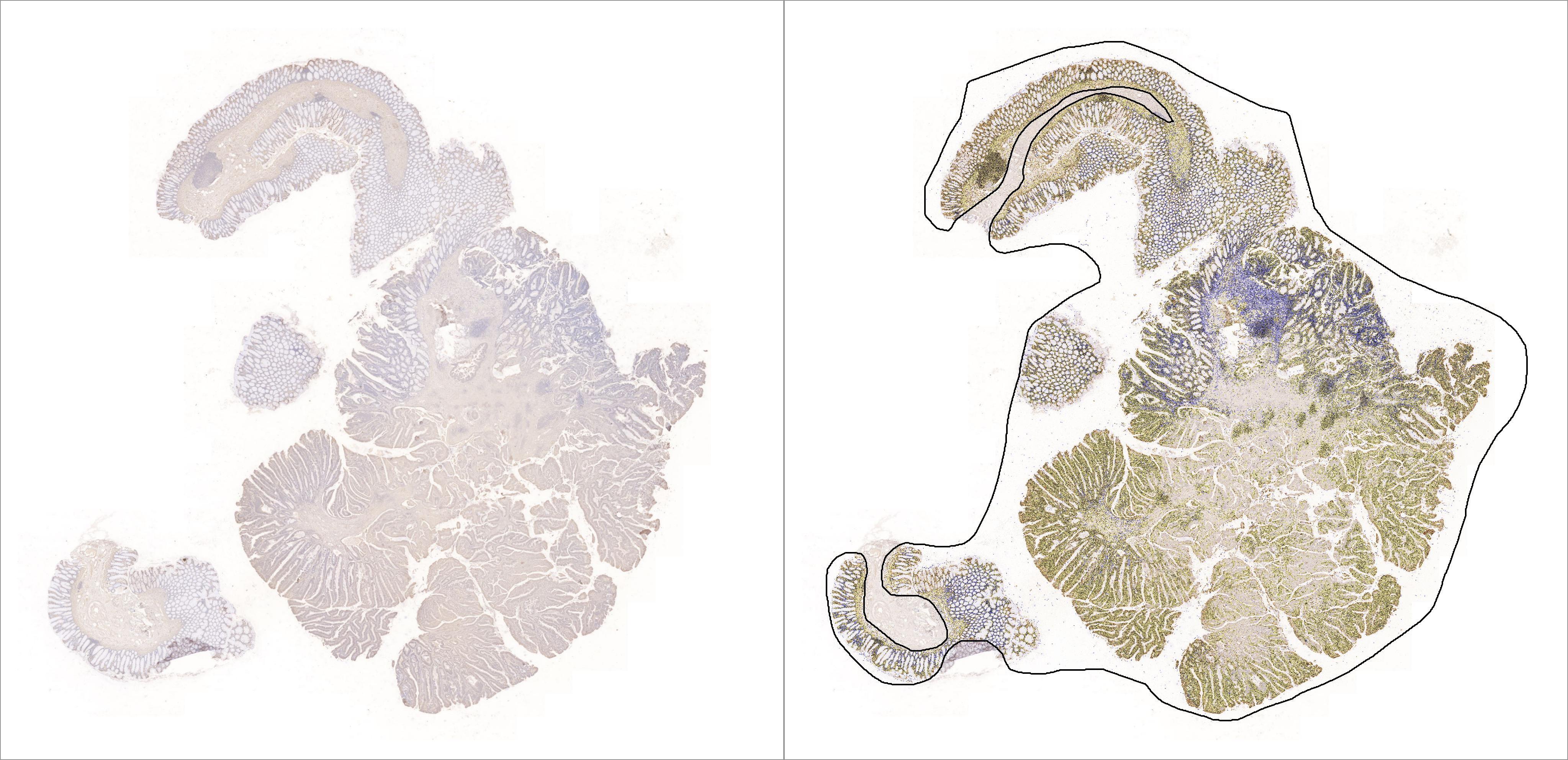


## 603557-10 CHMP7


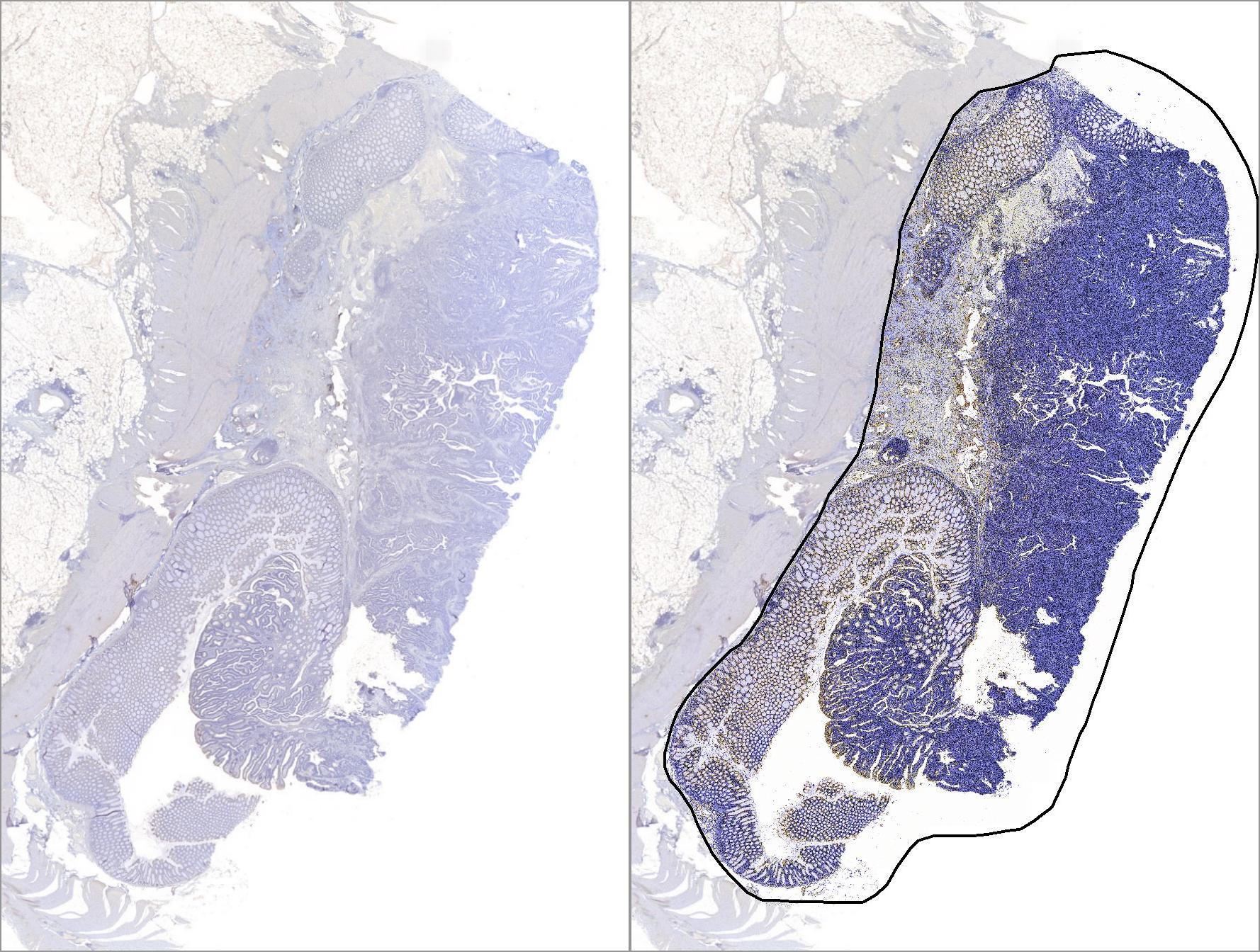


## 608785-16 CHMP7


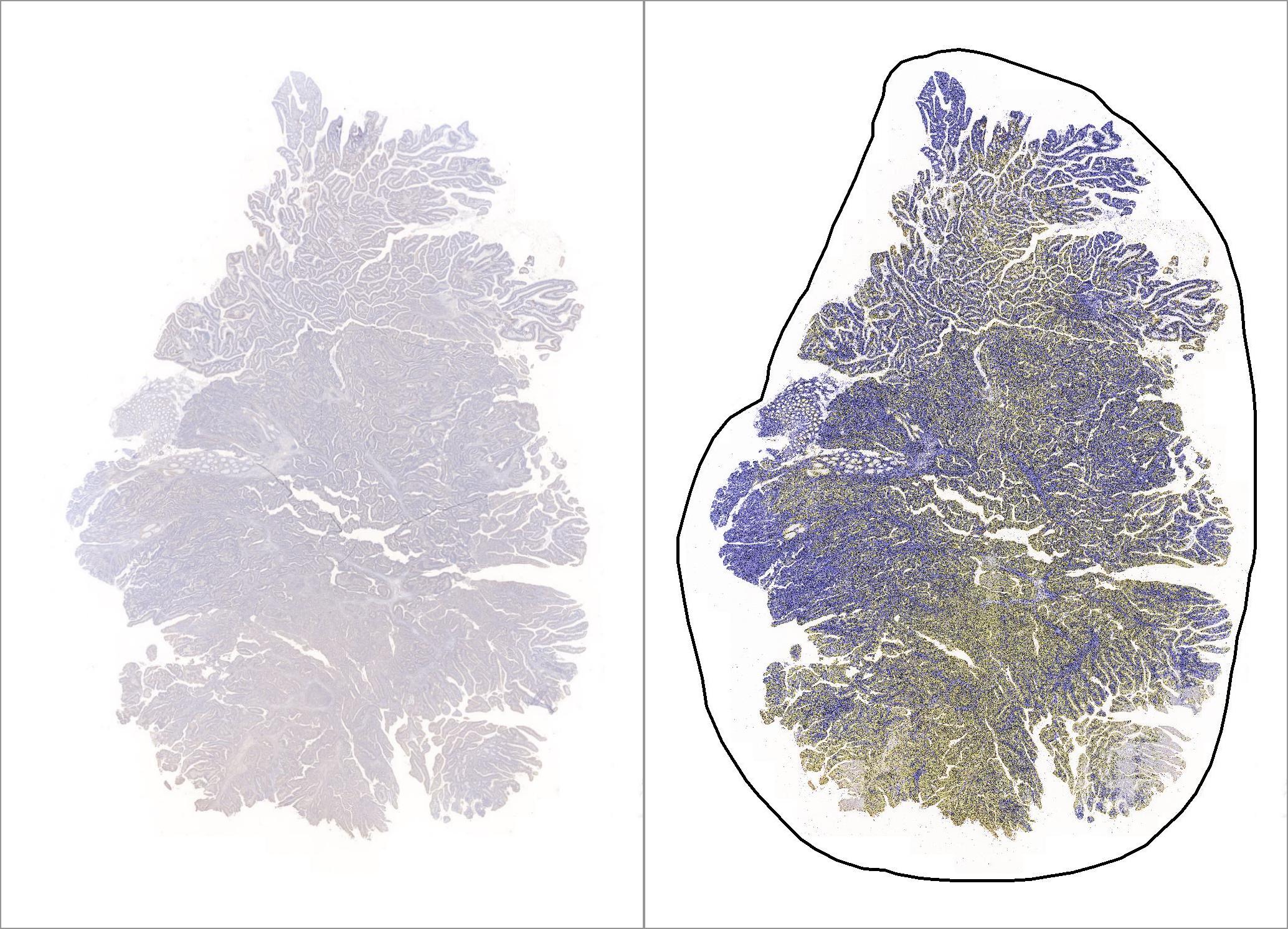


## 609741-16 CHMP7


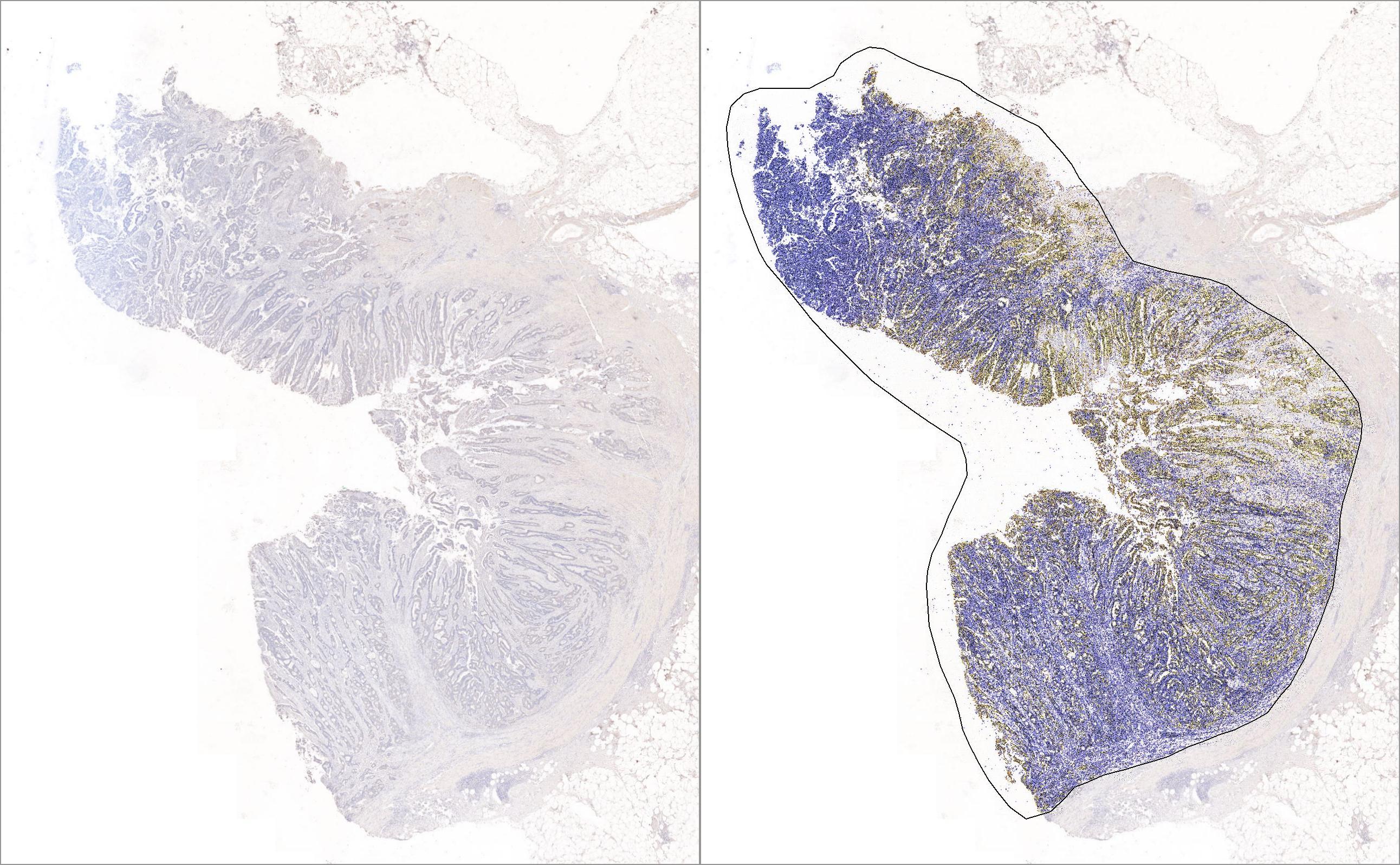


## 611632-13 CHMP7


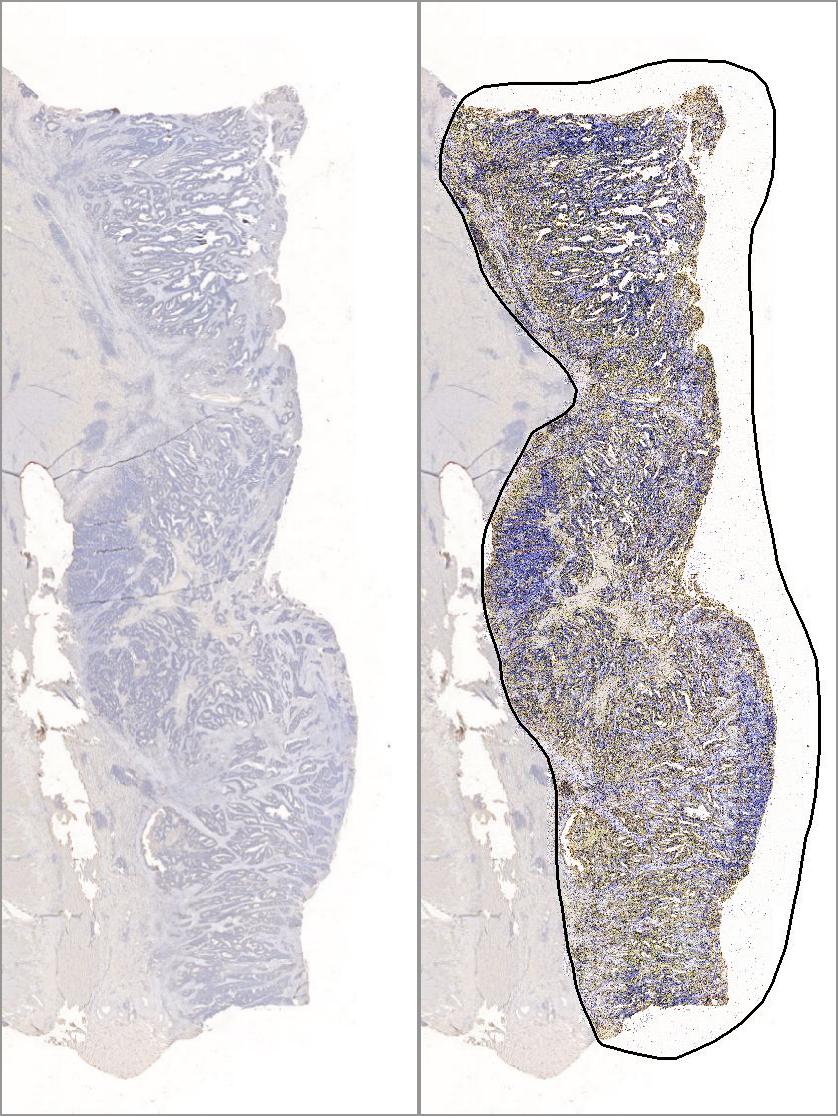


## 614884-3 CHMP7


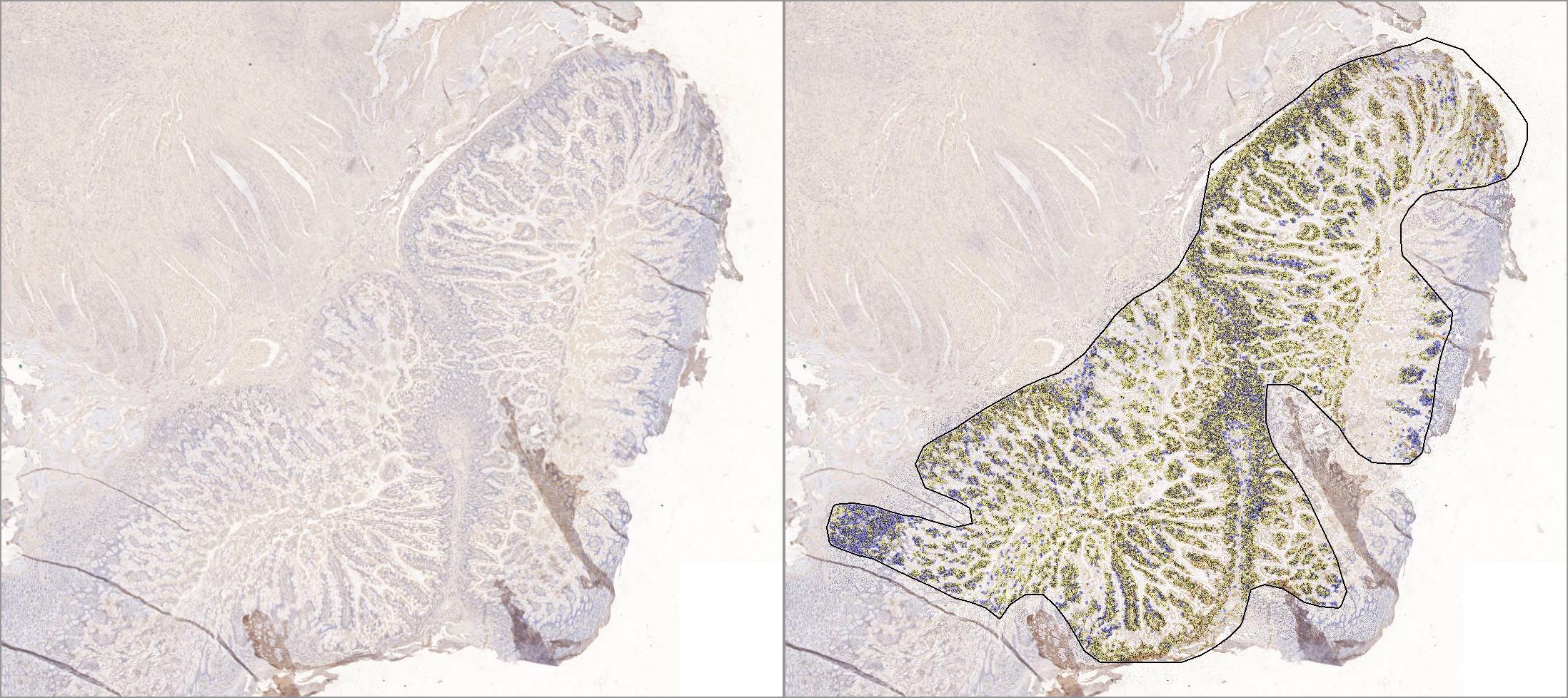


## 615409-18 CHMP7


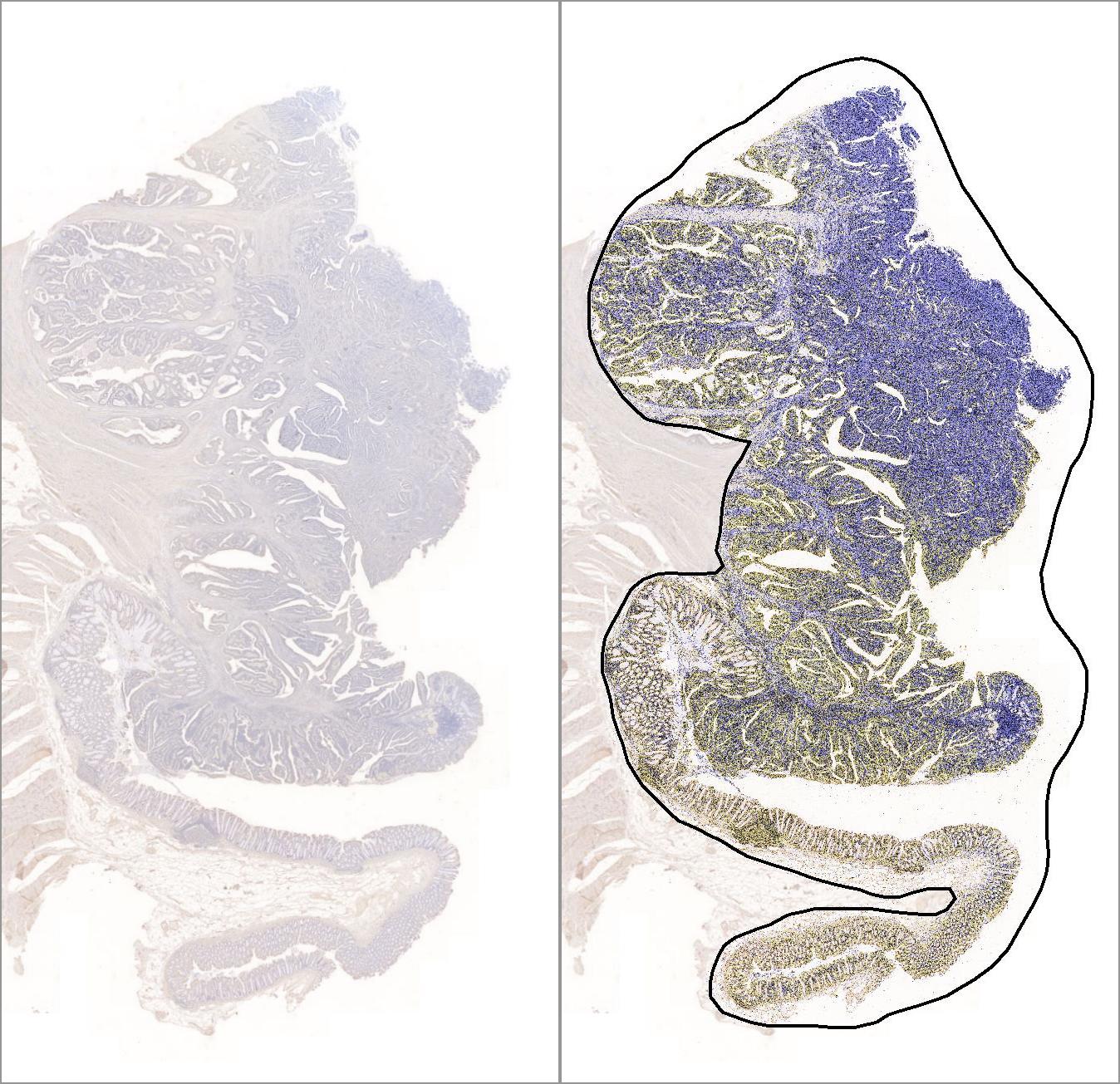


## 617188-14 CHMP7


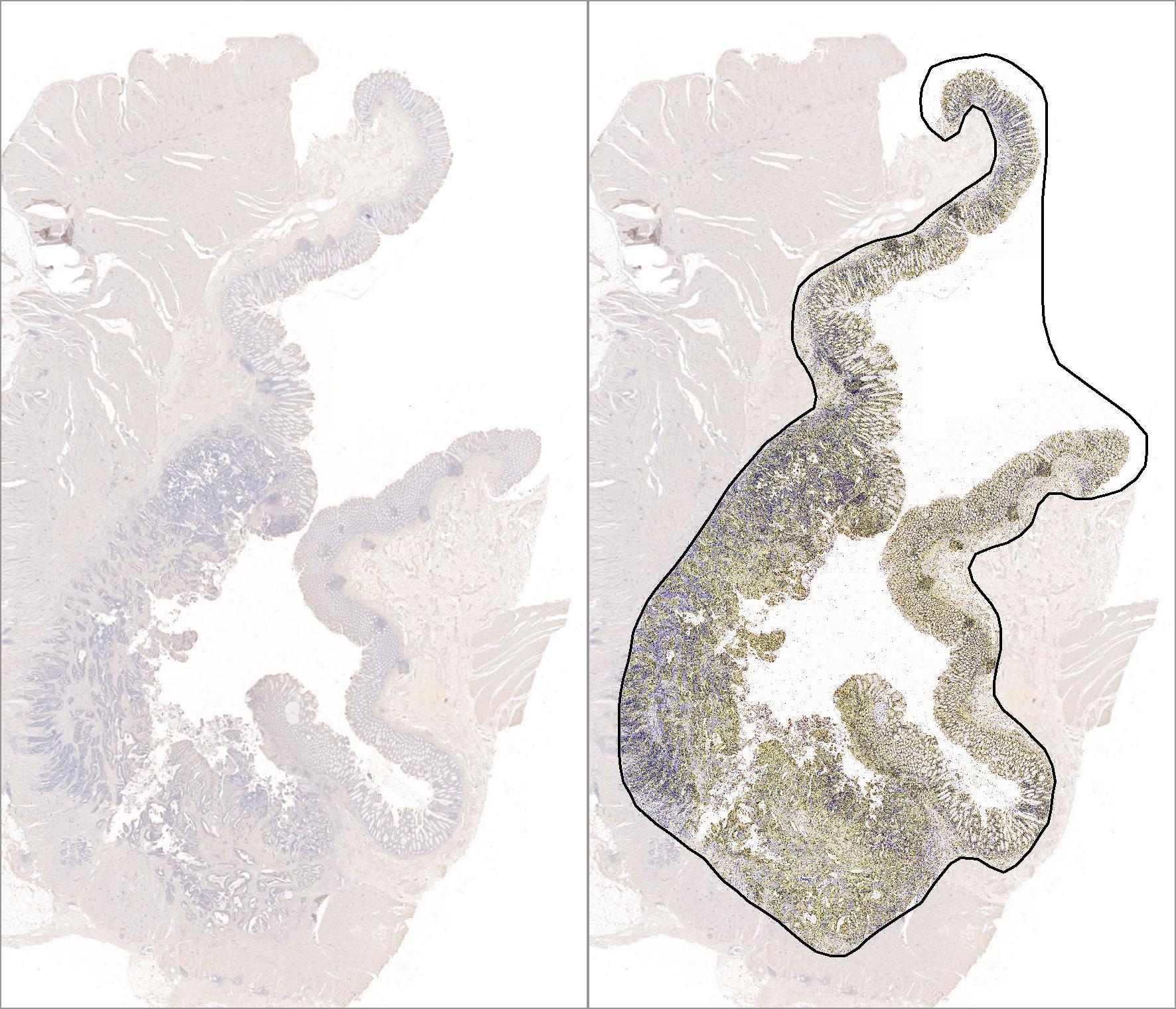


## 617602-7 CHMP7


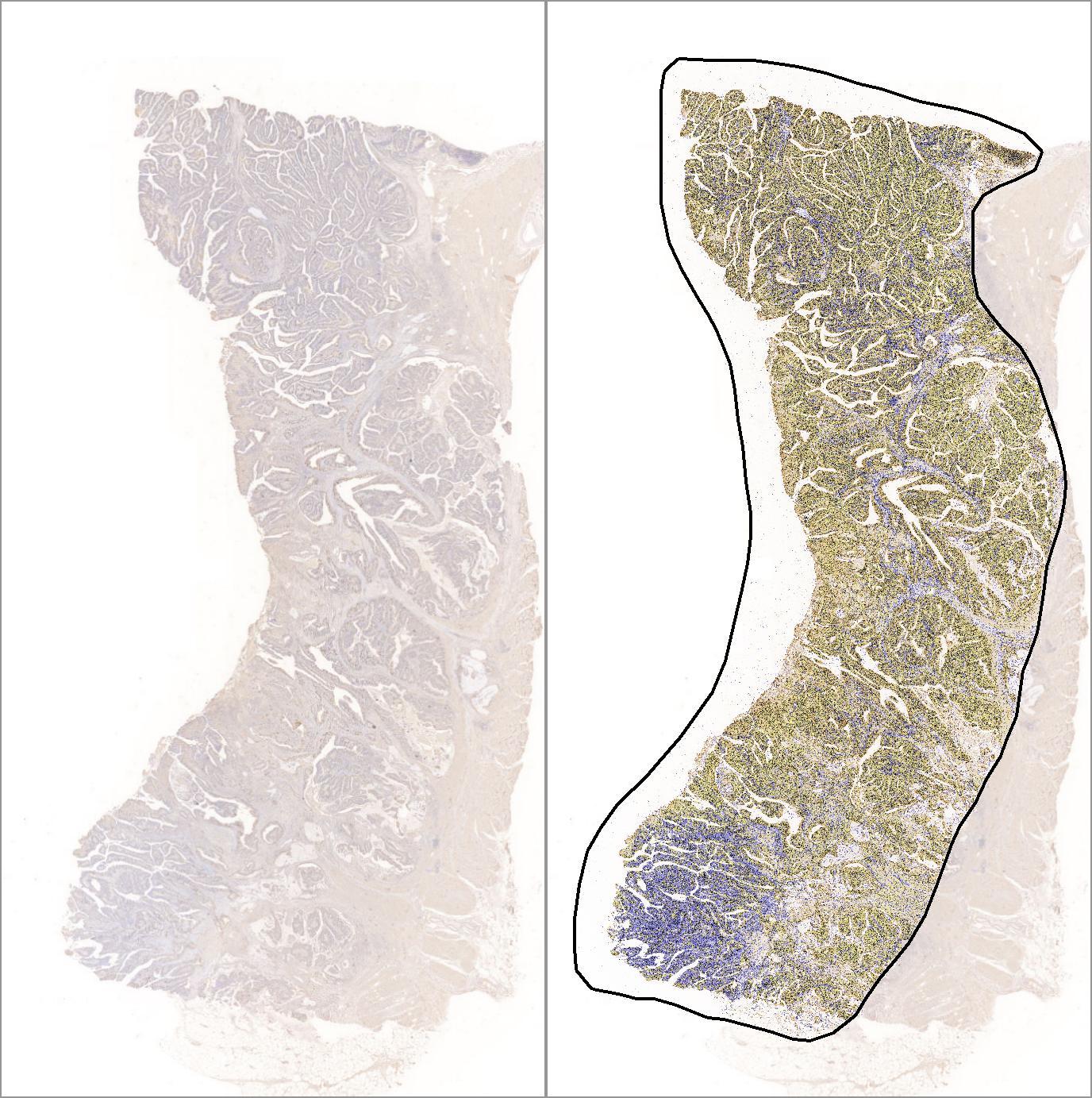


## 617806-20 CHMP7


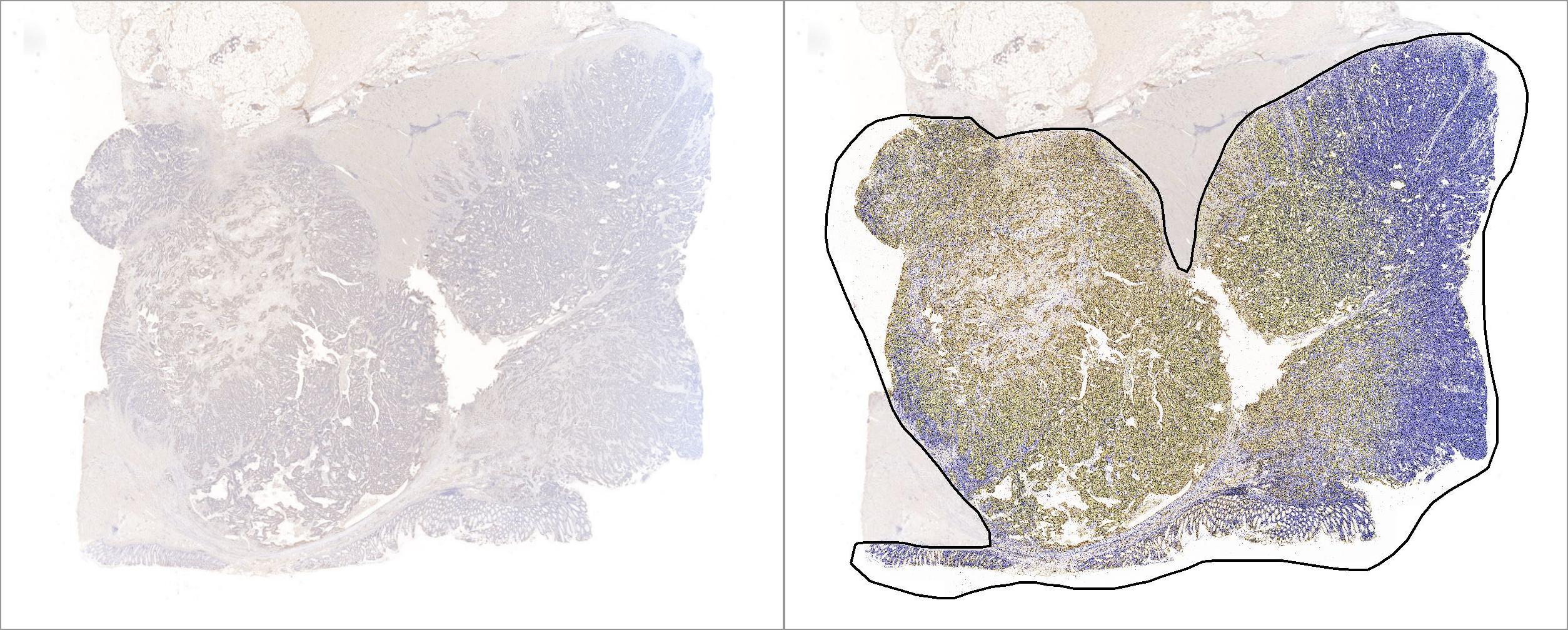


## 619686-13 CHMP7


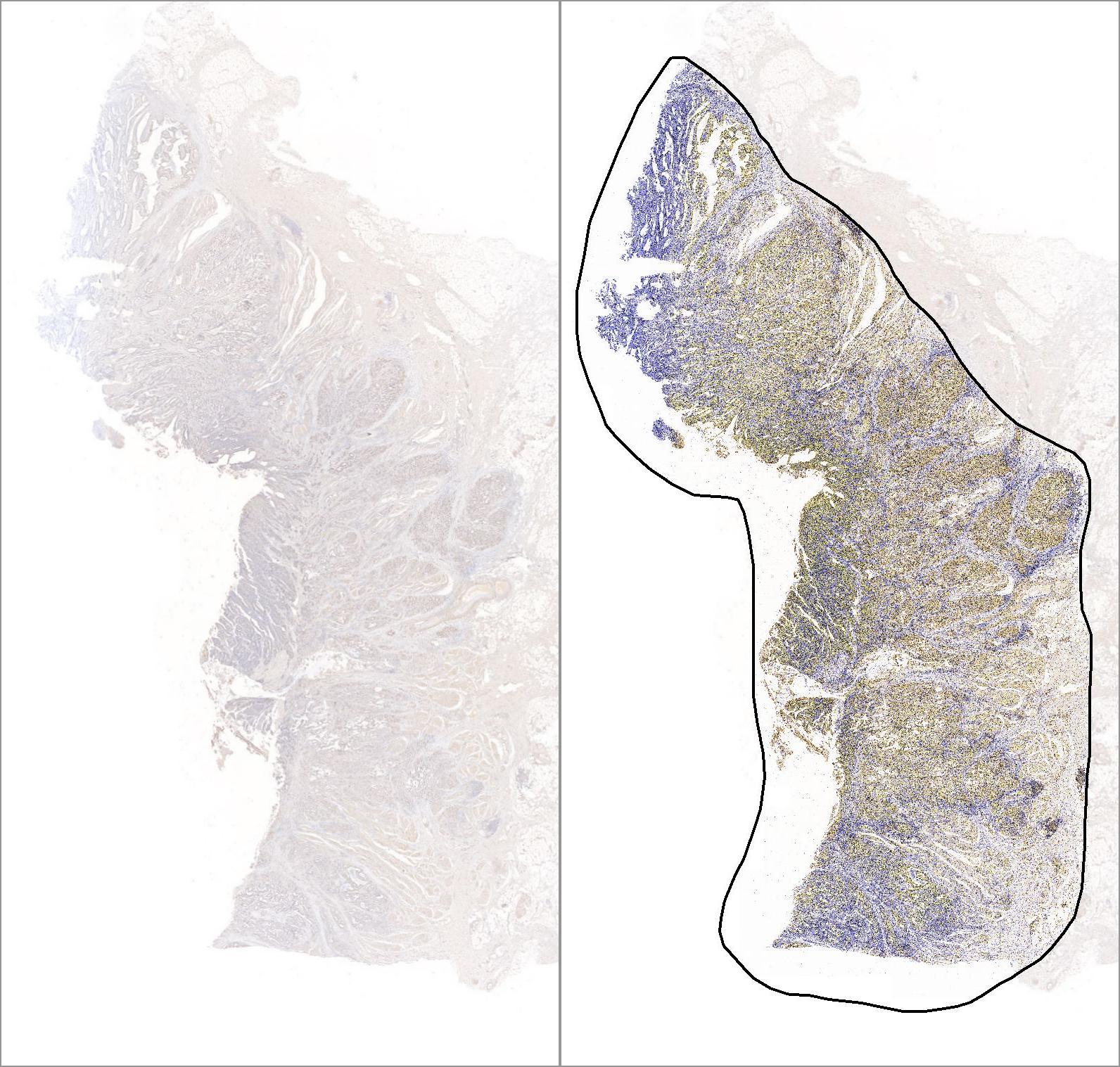


## 621360-15 CHMP7


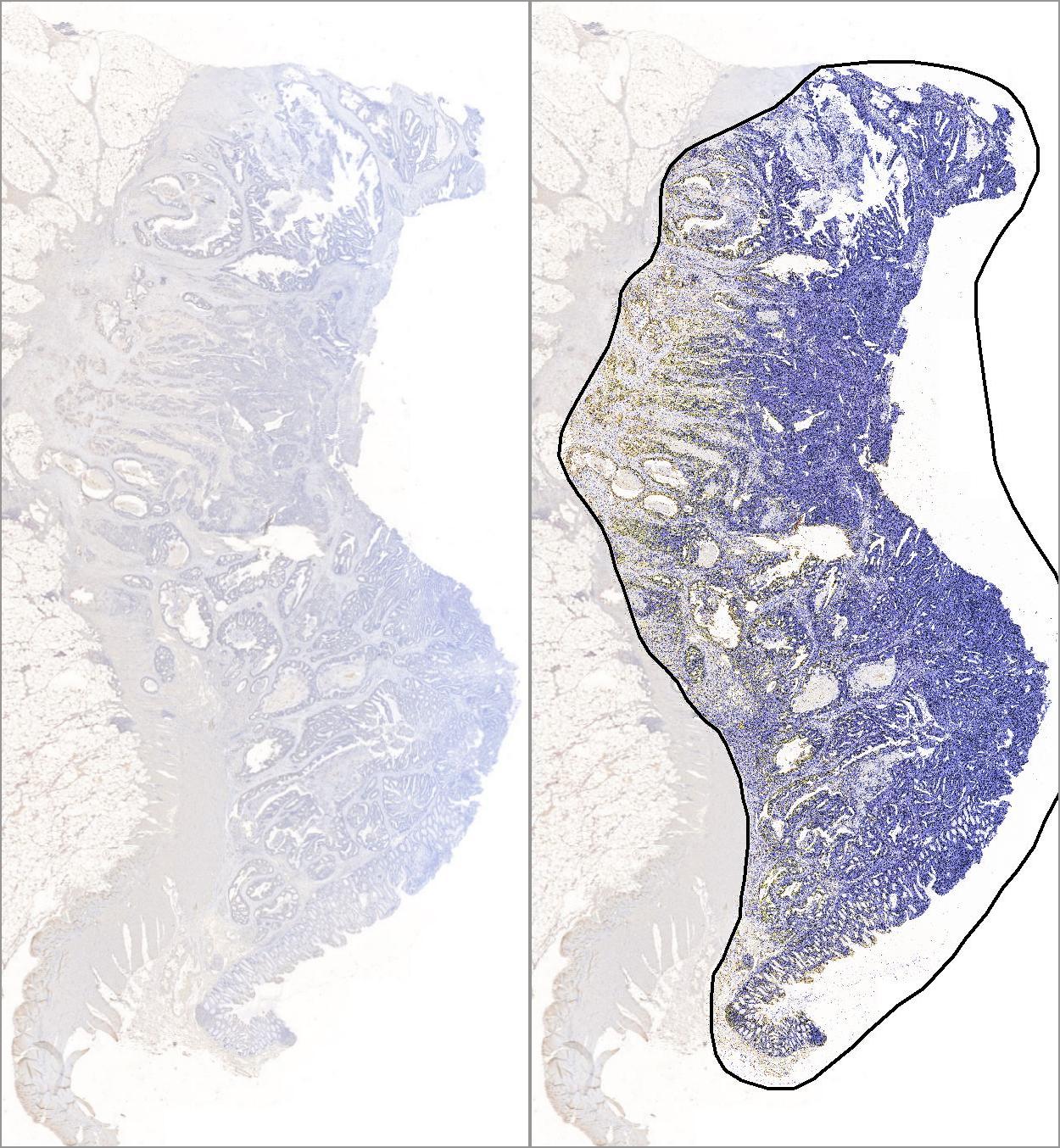


## 621603-4 CHMP7


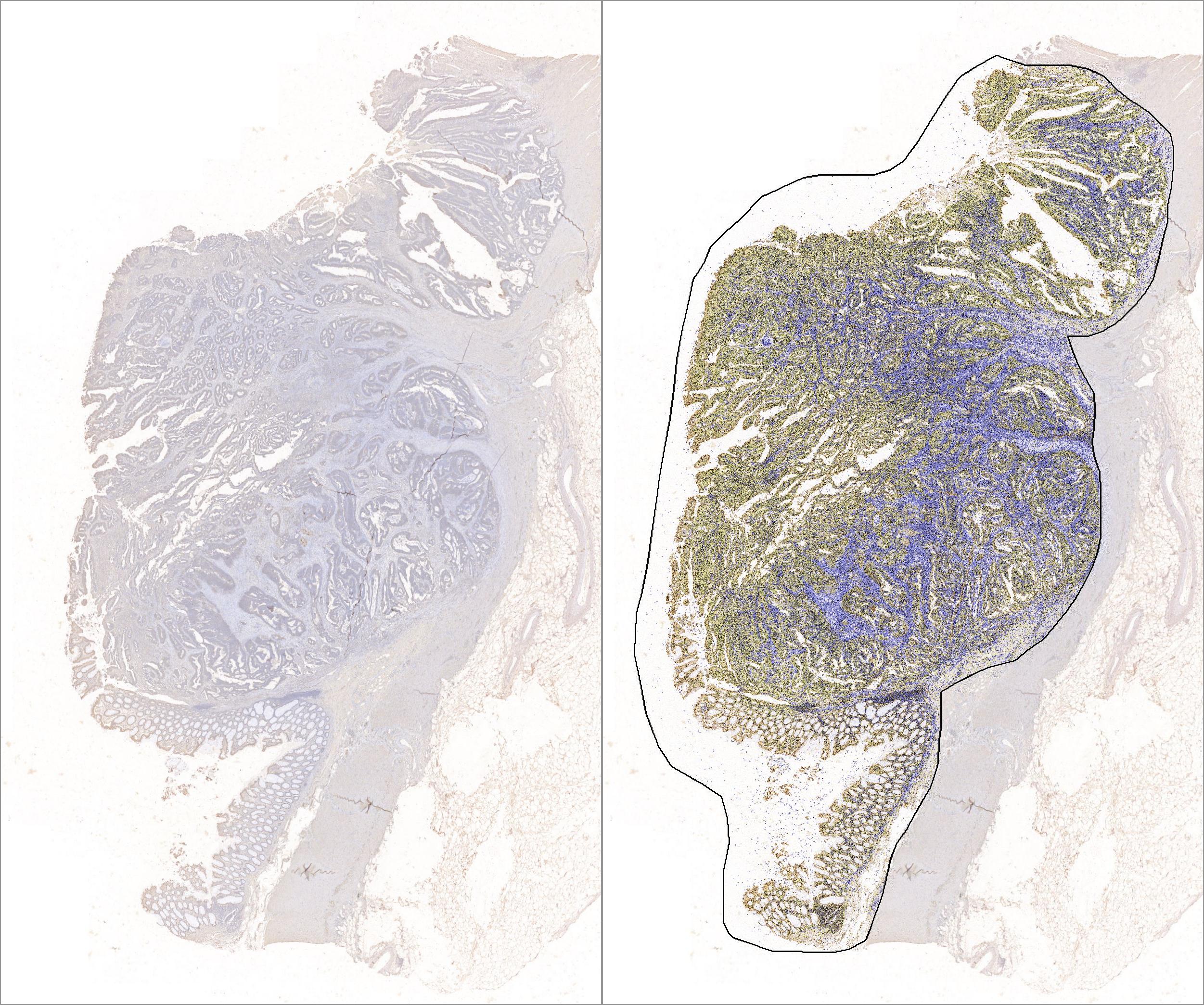


## 622744-28 CHMP7


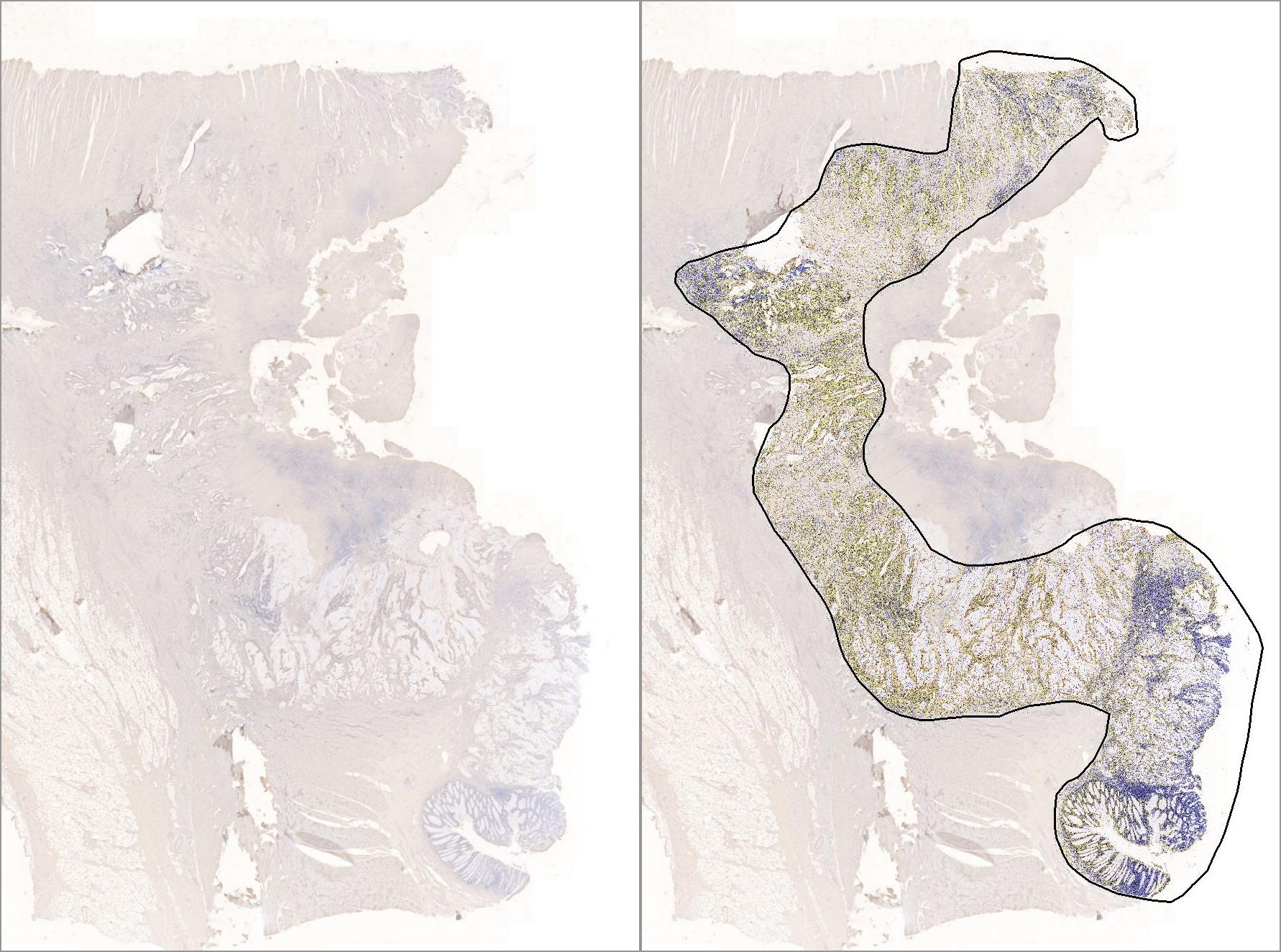


## 622999-6 CHMP7


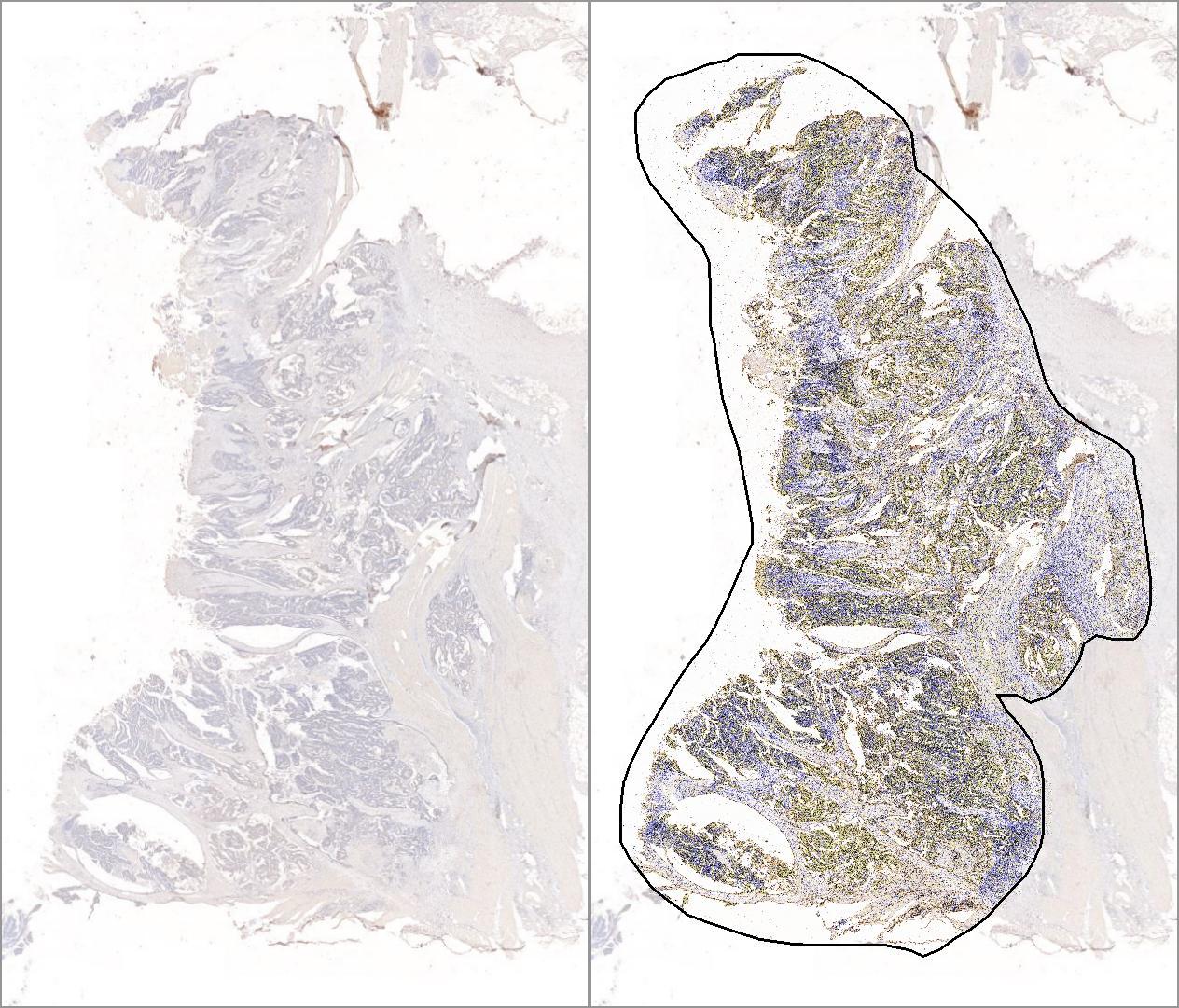


## 625303-14 CHMP7


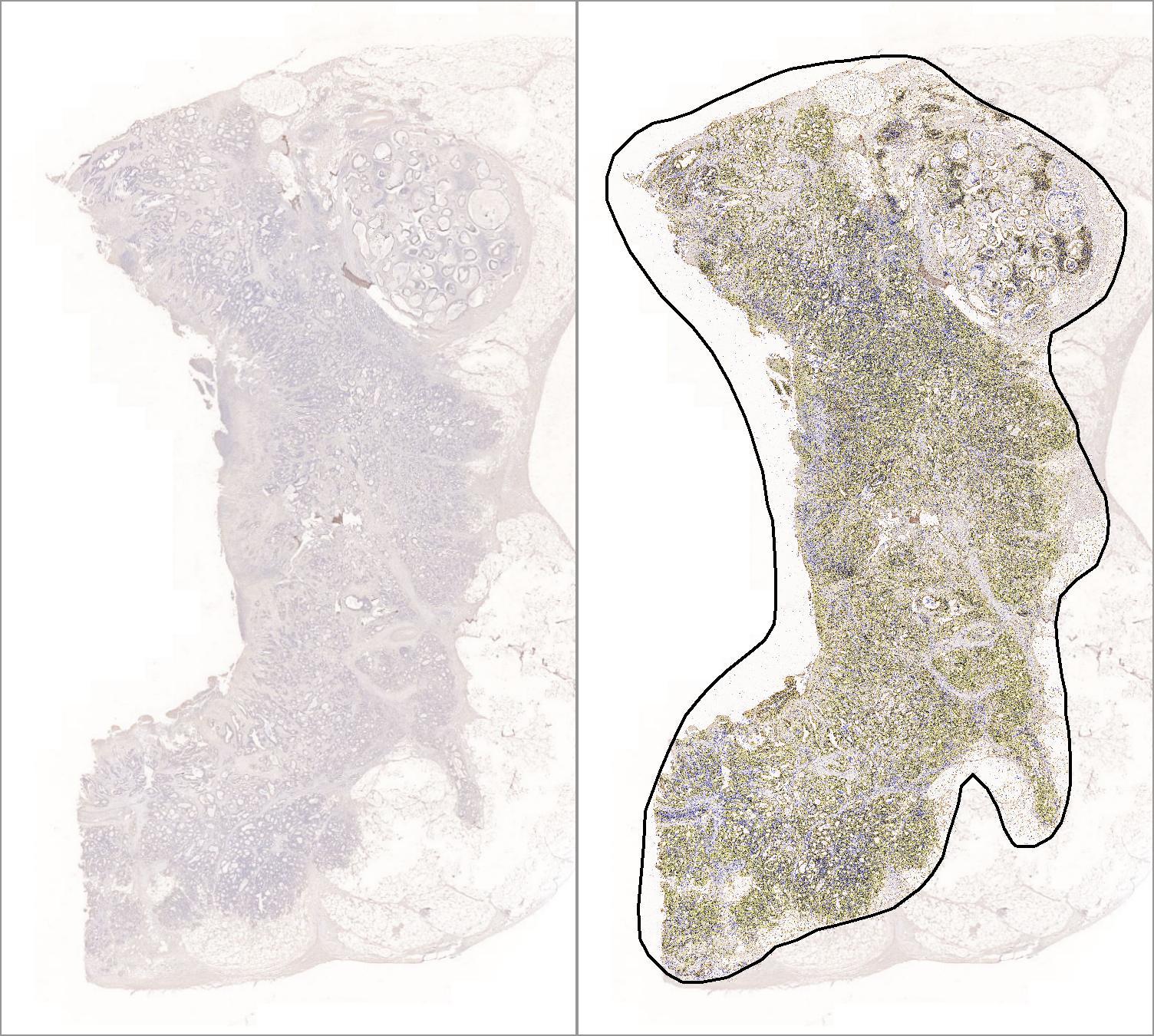


## 626312-5 CHMP7


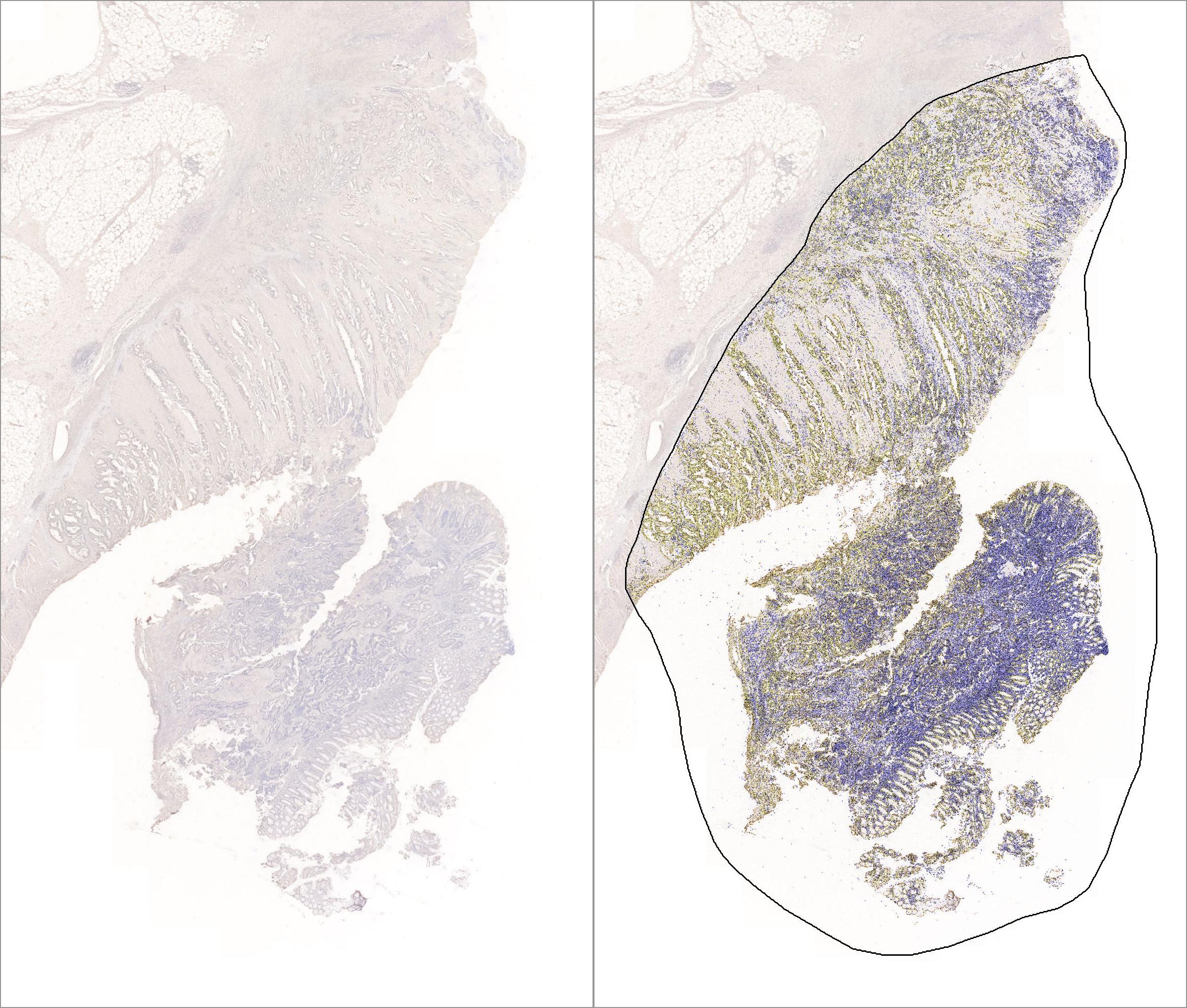


## 626983-13 CHMP7


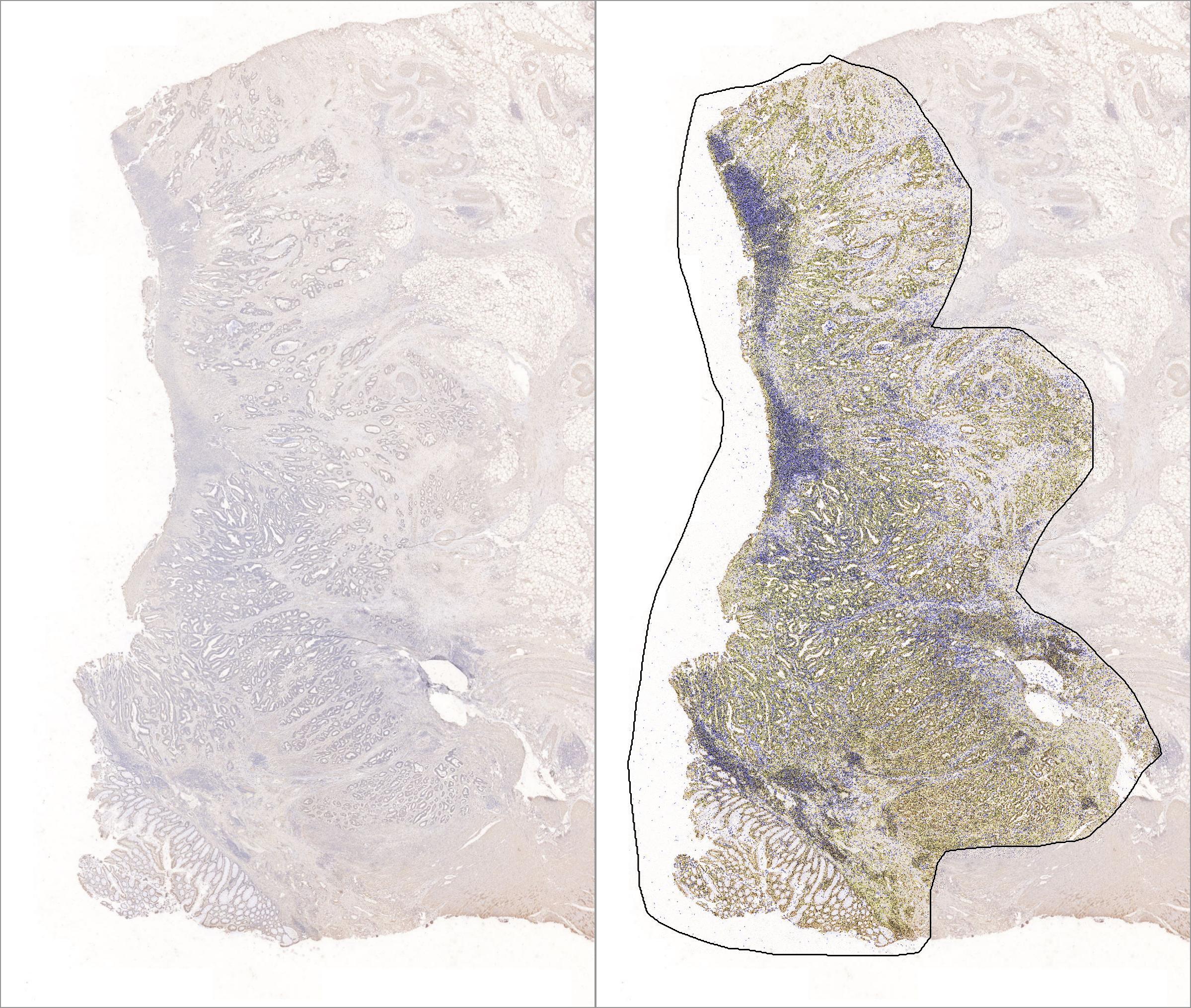


## 631719-5 CHMP7


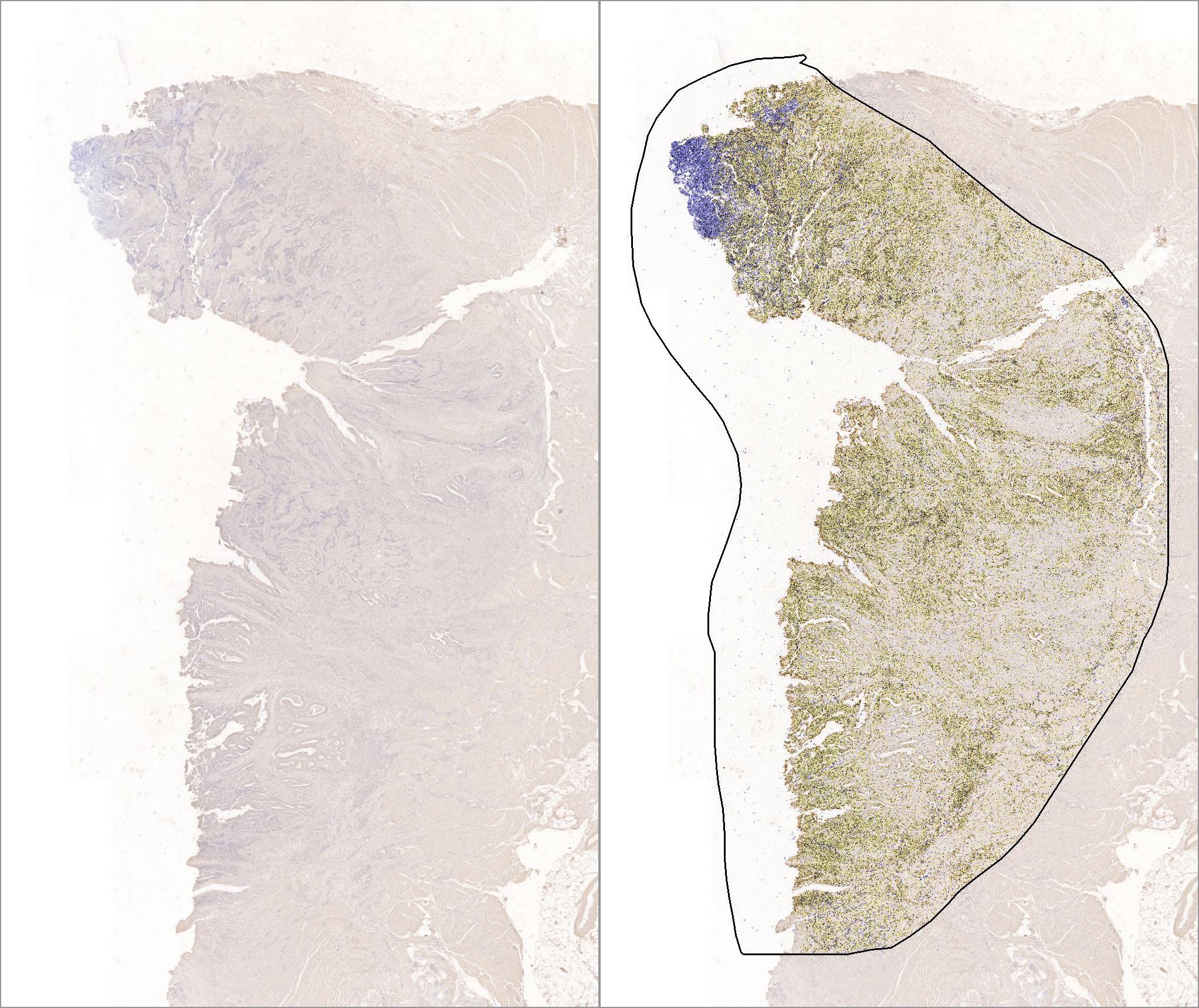


## 635487-13 CHMP7


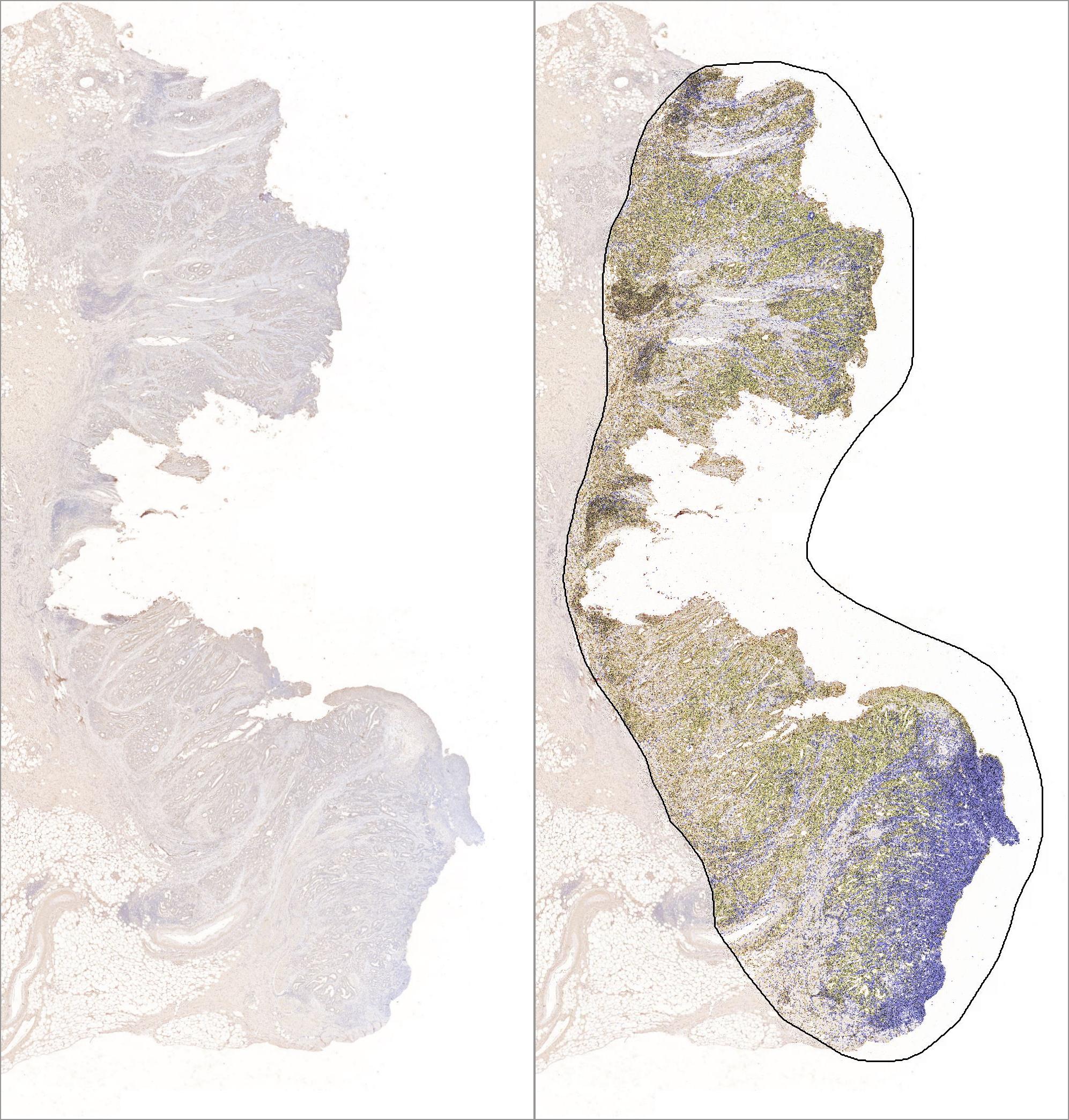


## 635804-17 CHMP7


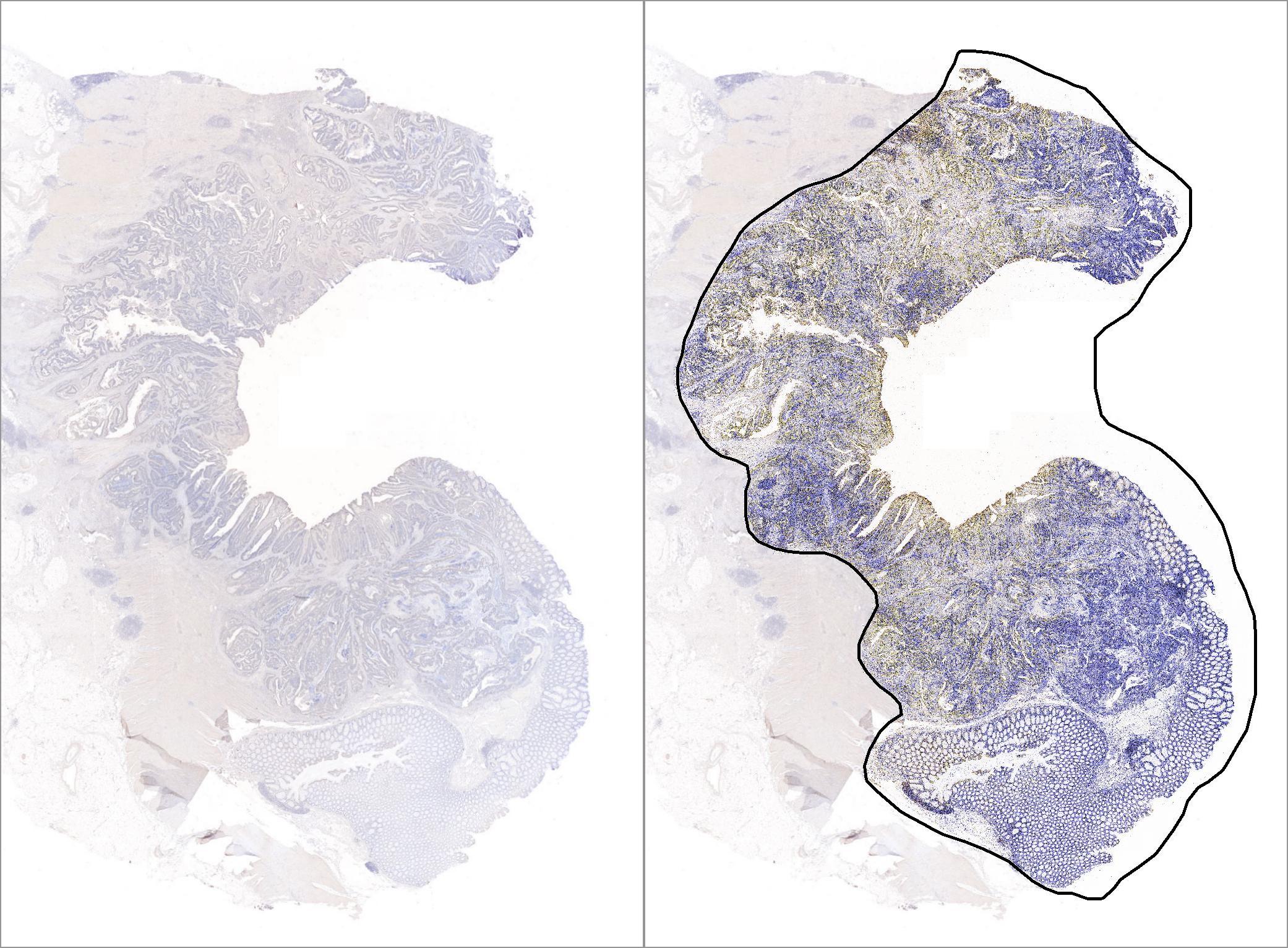


## 635835-14 CHMP7


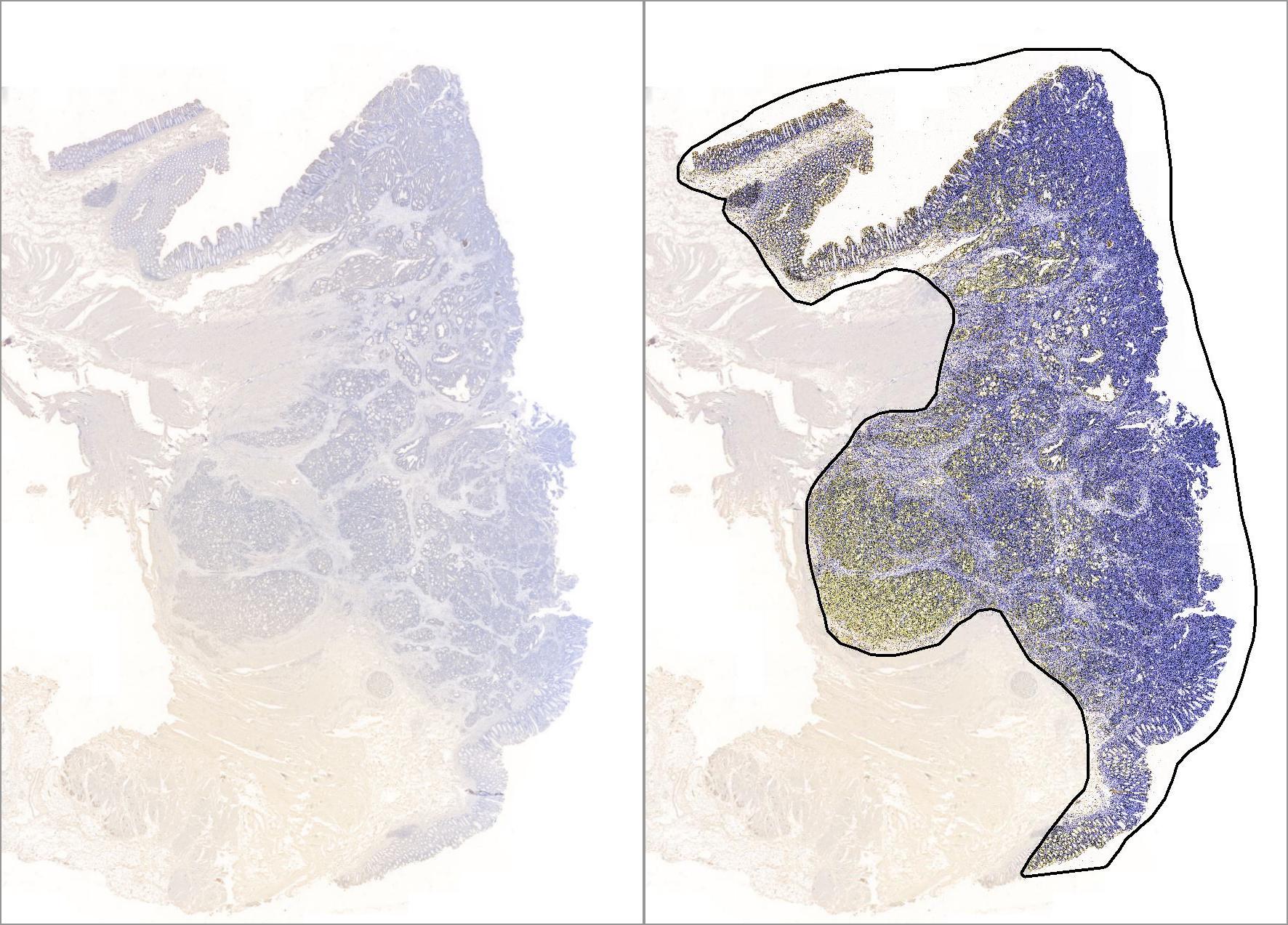


## 636271-13 CHMP7


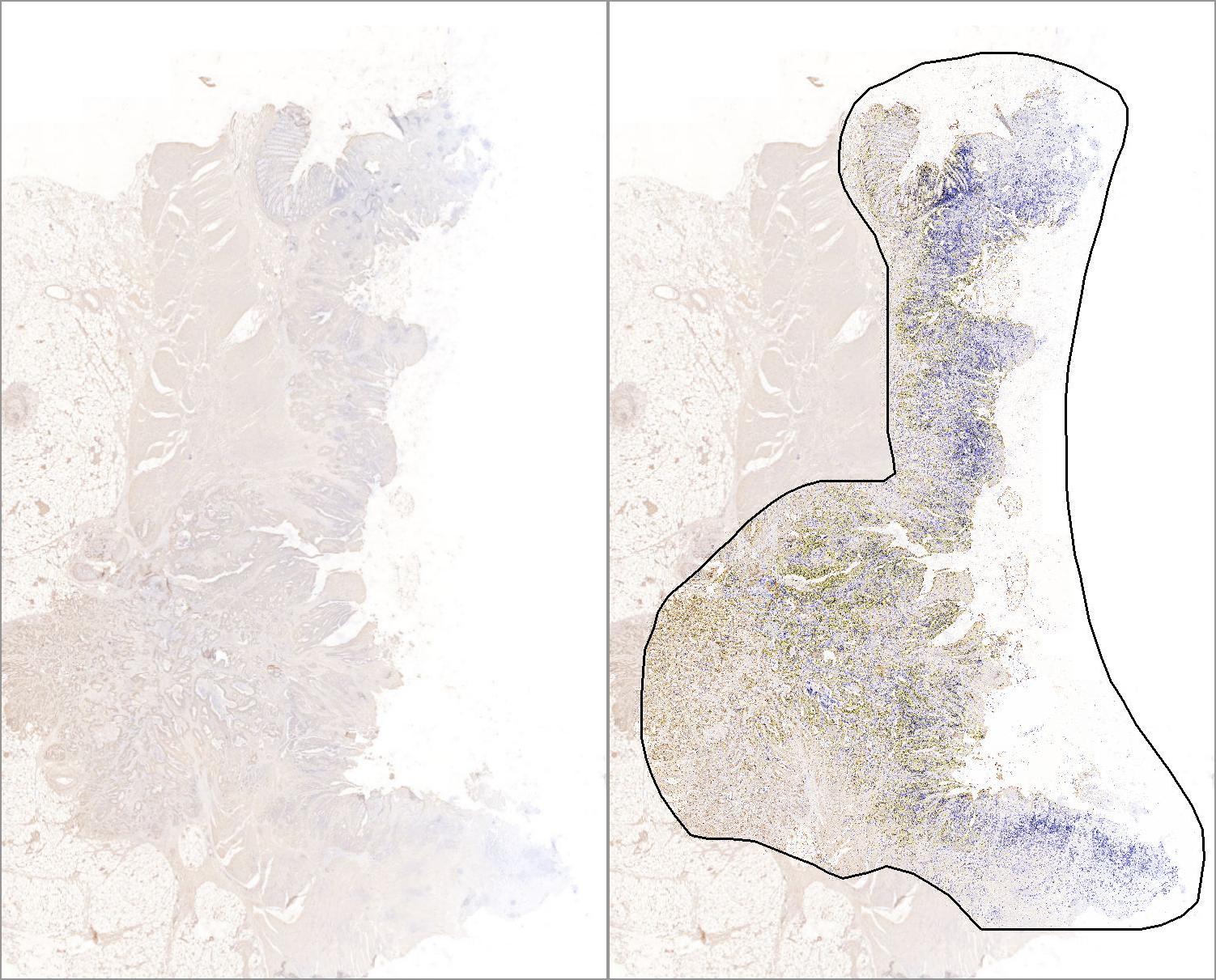


## 636295-15 CHMP7


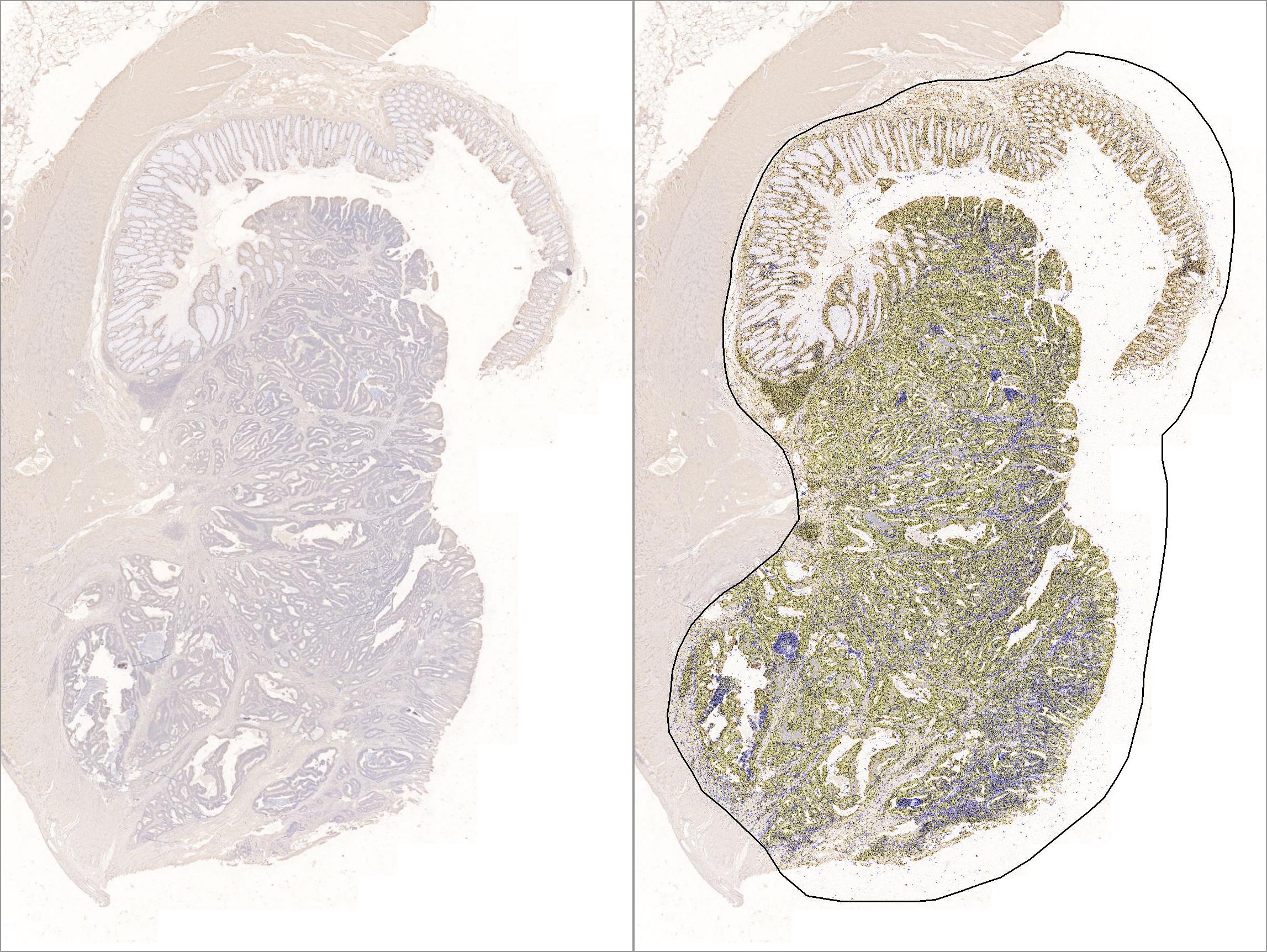


## 637404-10 CHMP7


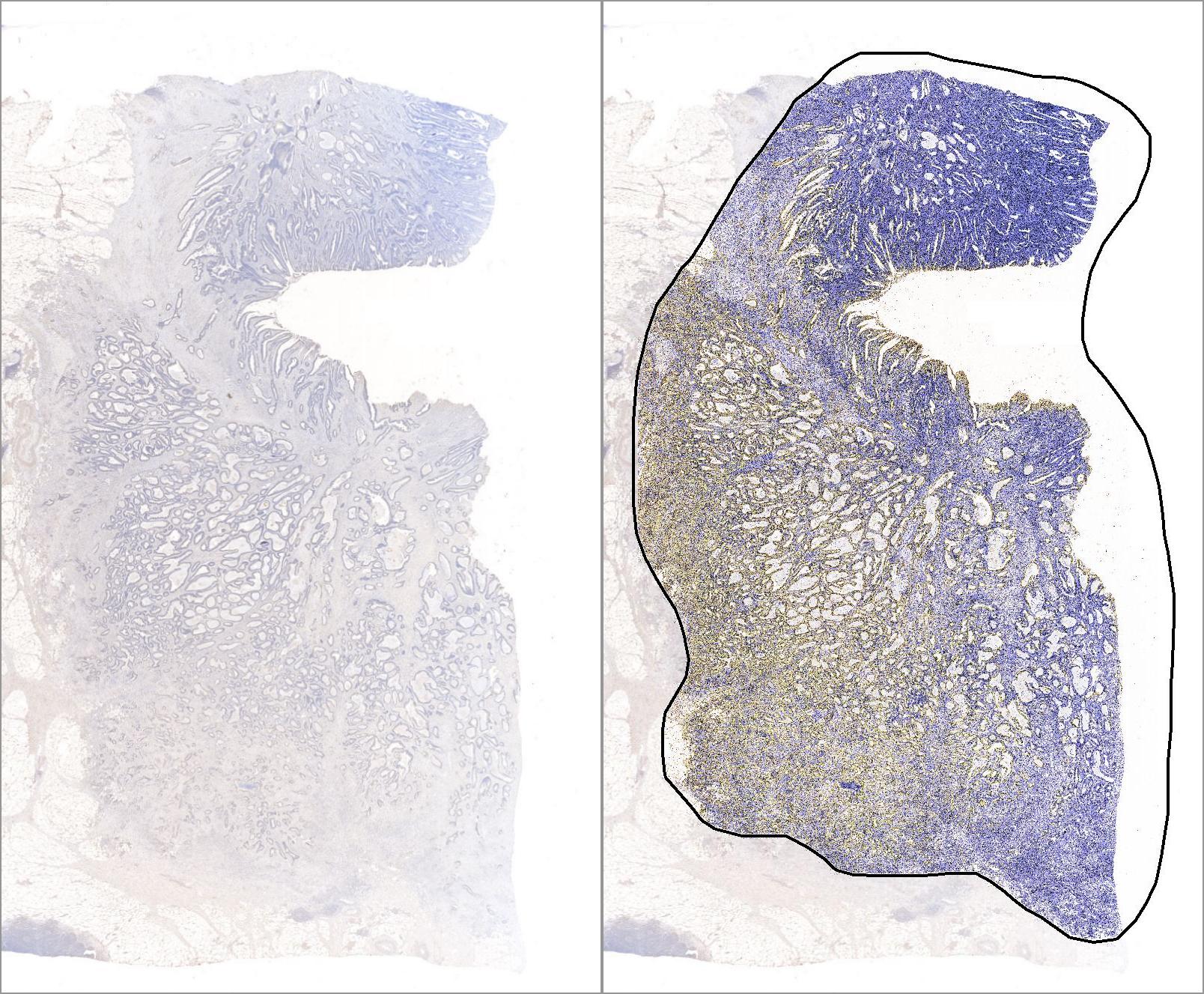


## 639357-8 CHMP7


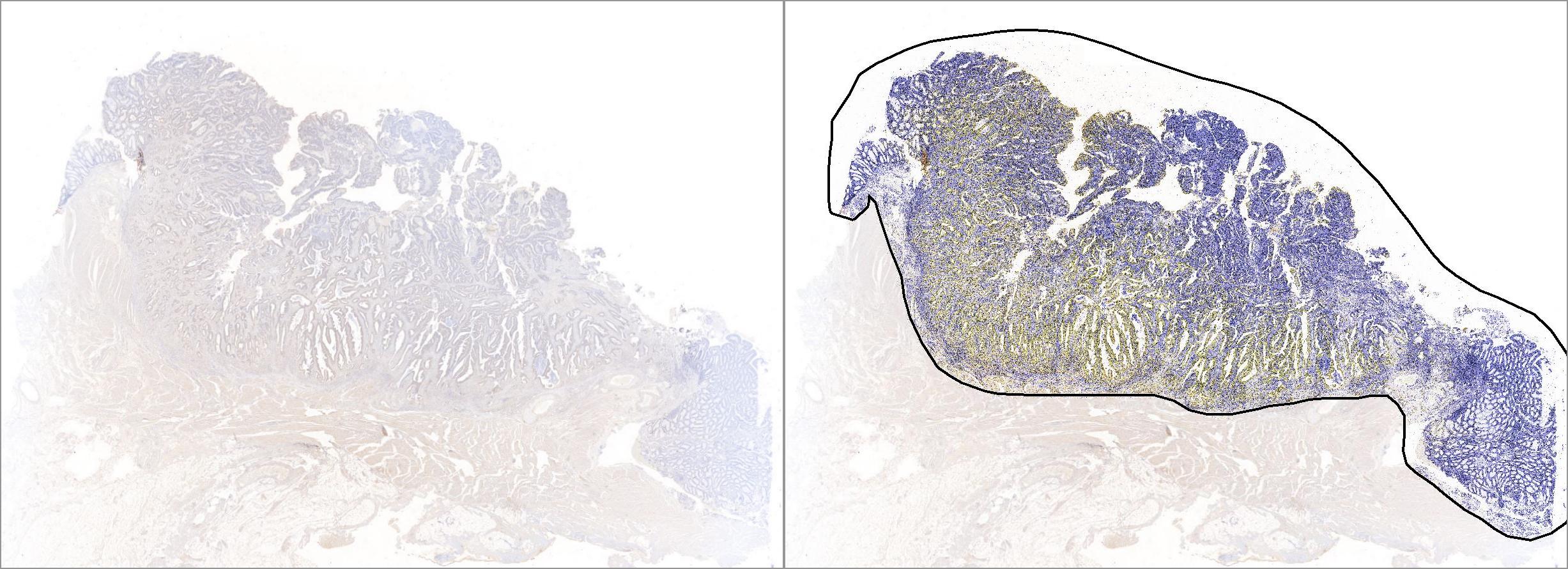


## 641462-9 CHMP7


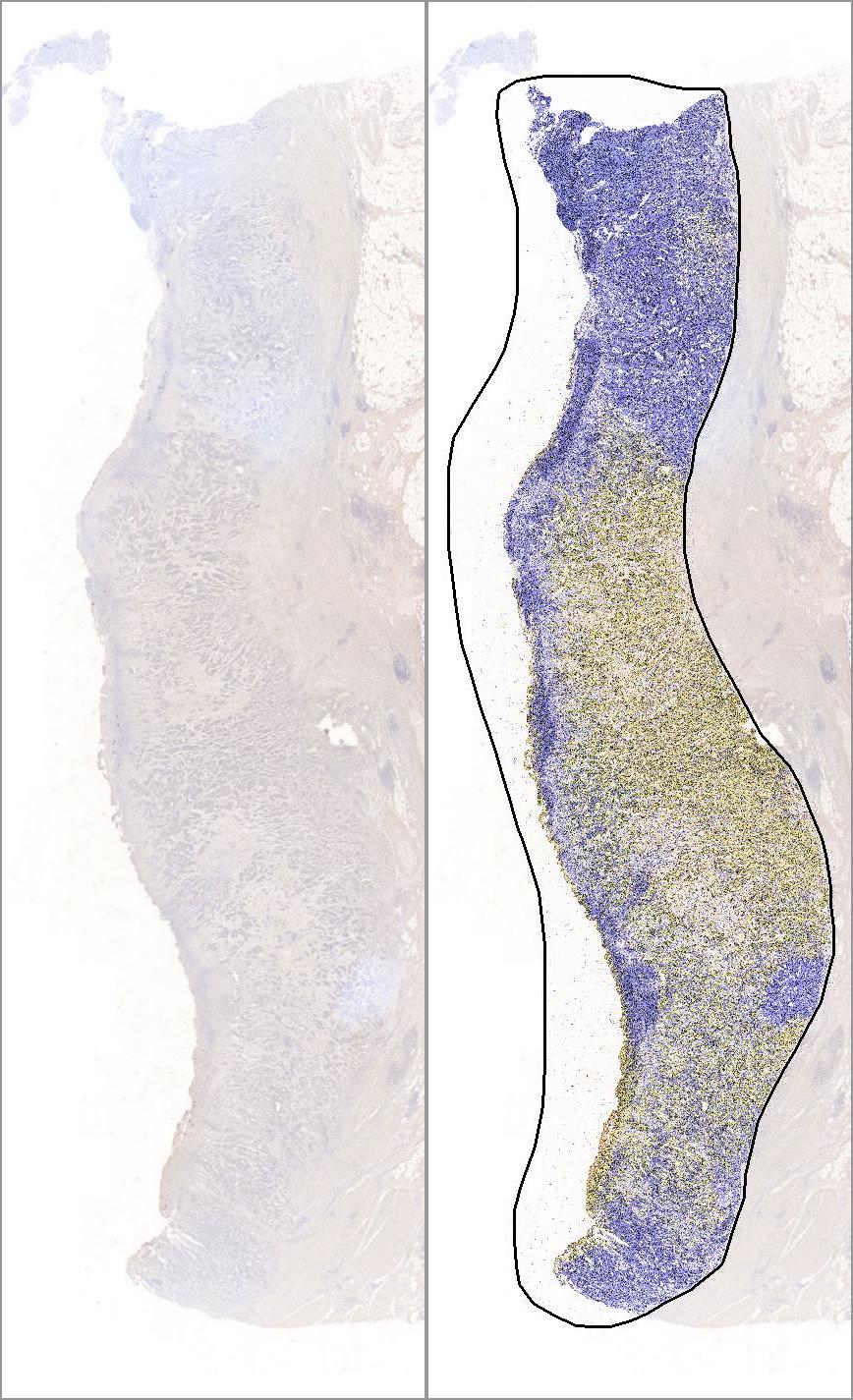


## 643143-8 CHMP7


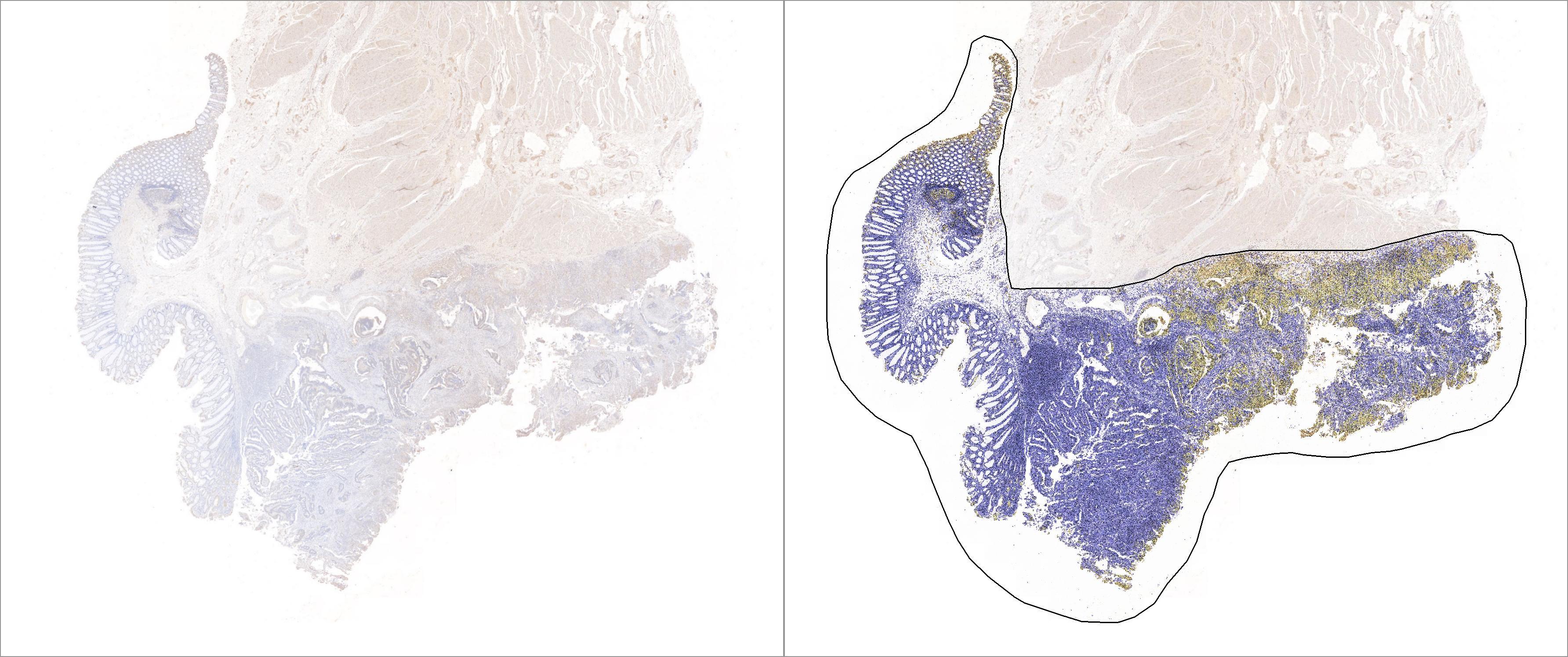


## 644966-13 CHMP7


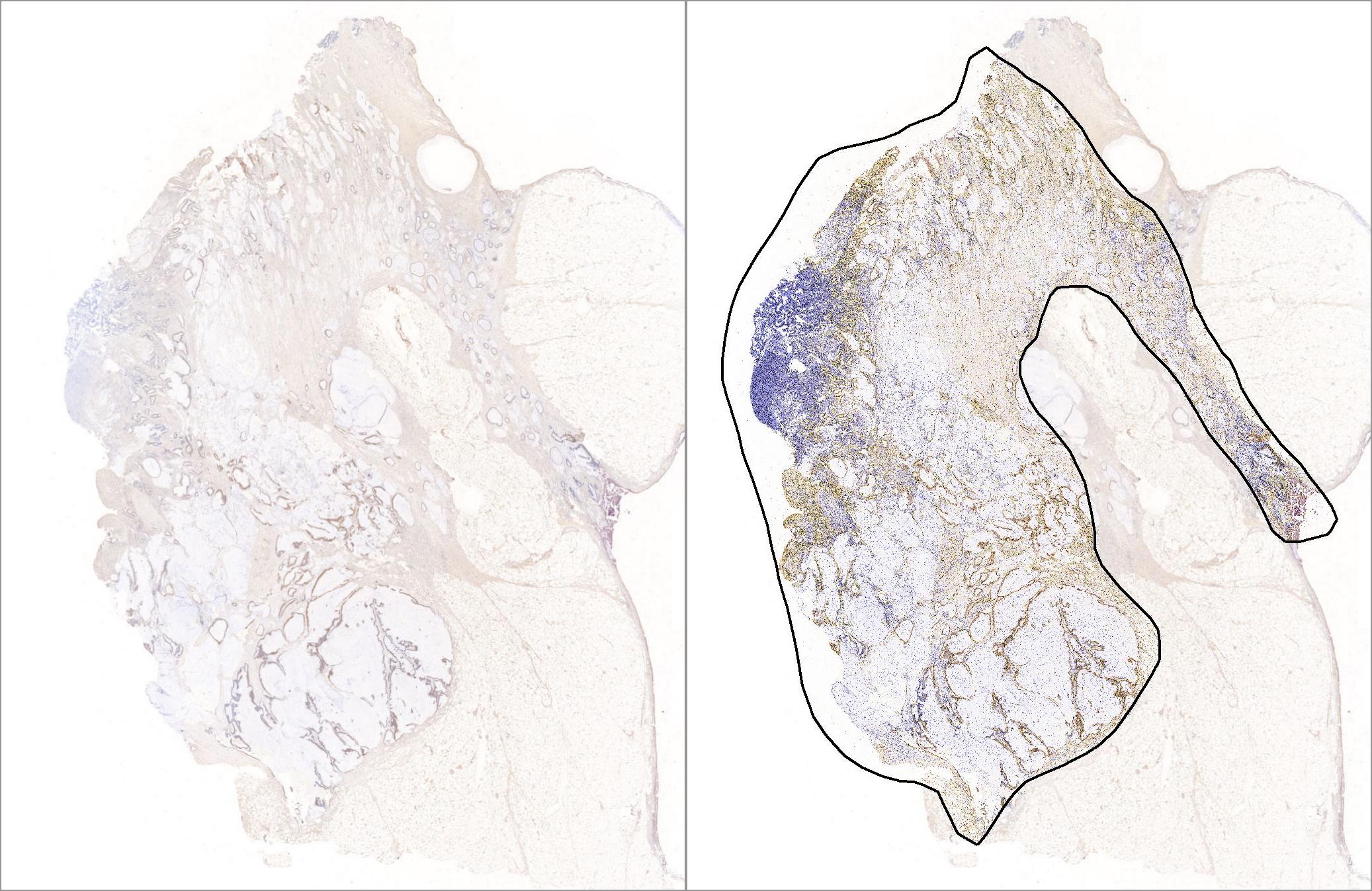


## 646389-6 CHMP7


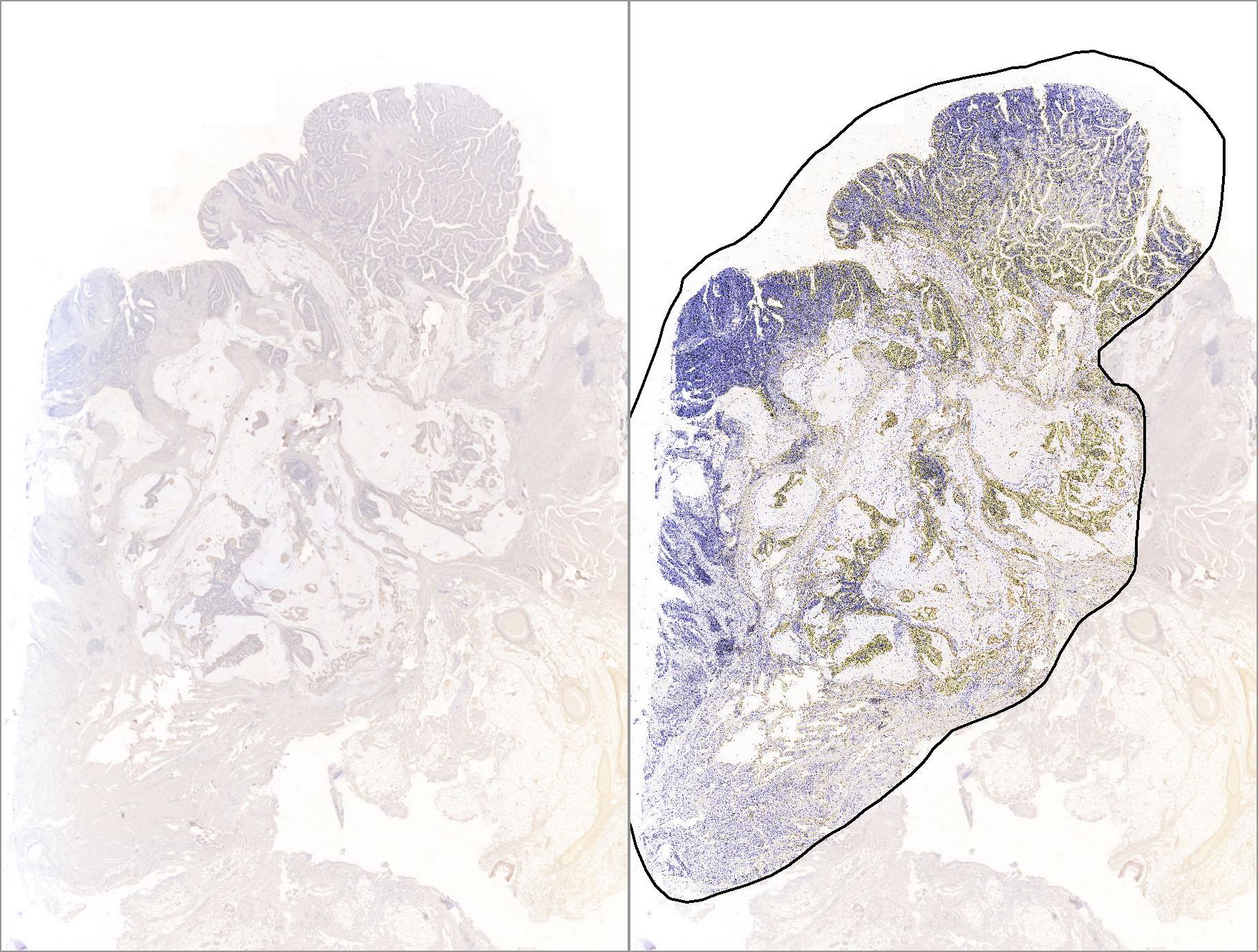


## 650590-13 CHMP7


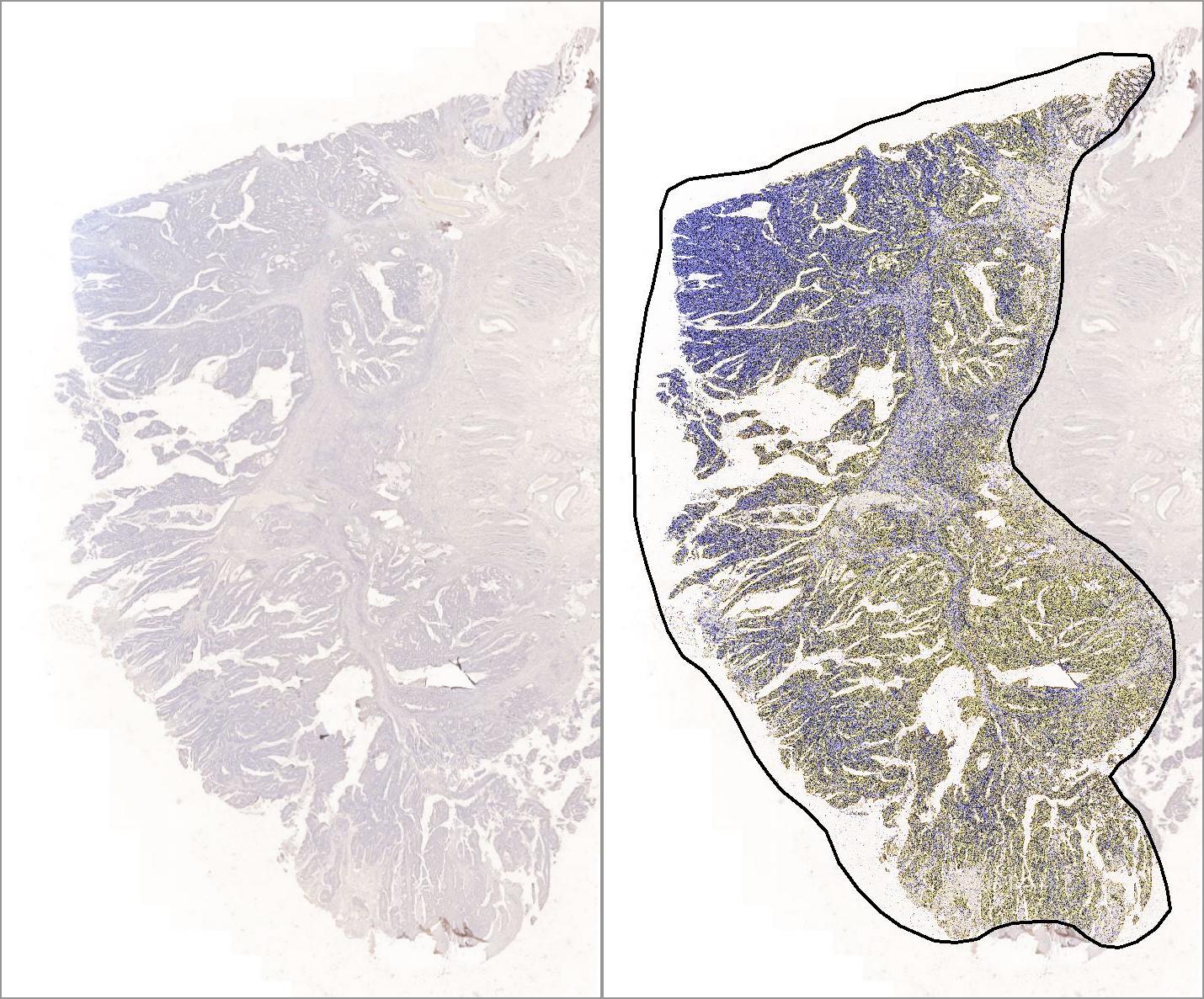


## 653827-12 CHMP7


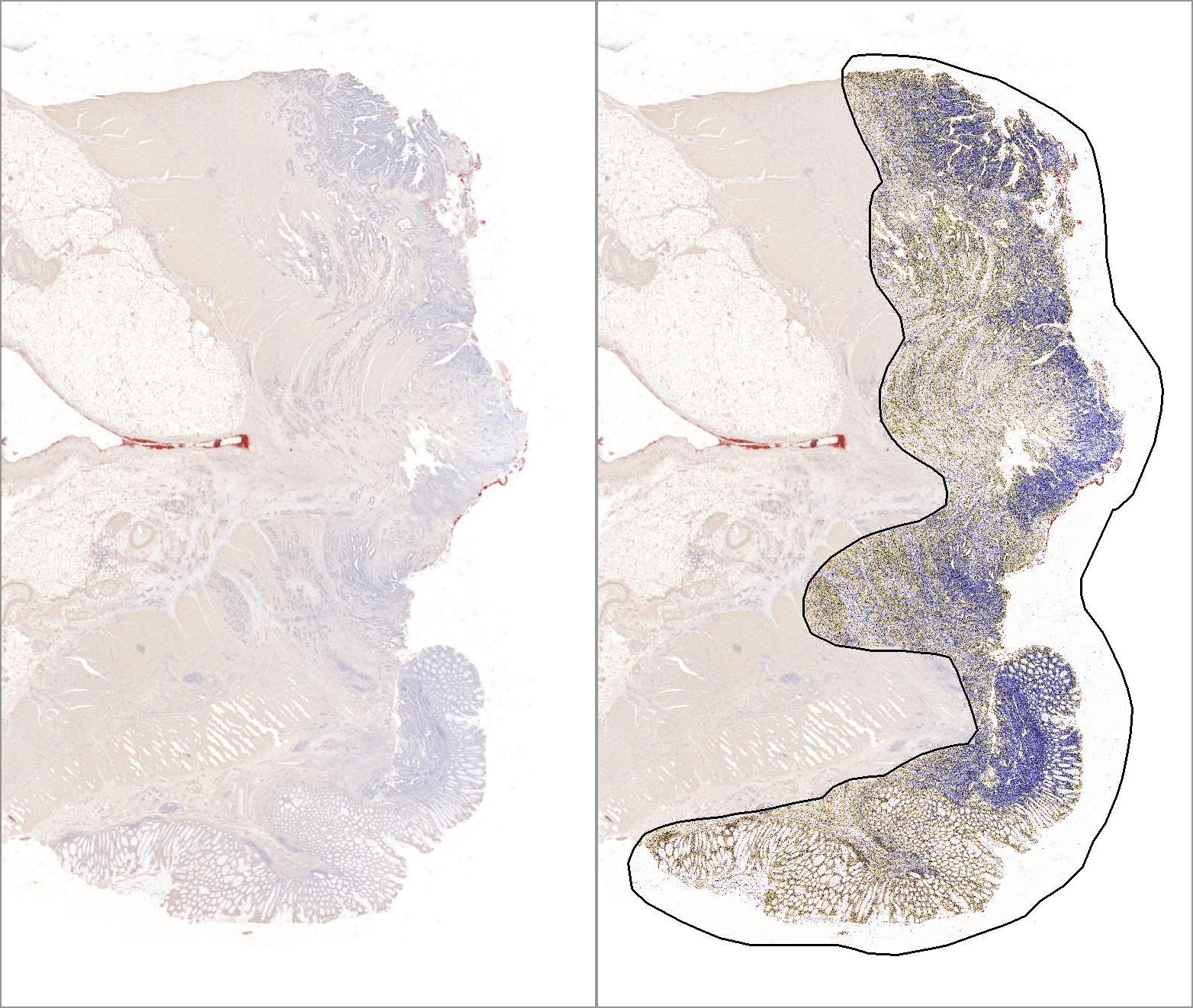


## 654746-14 CHMP7


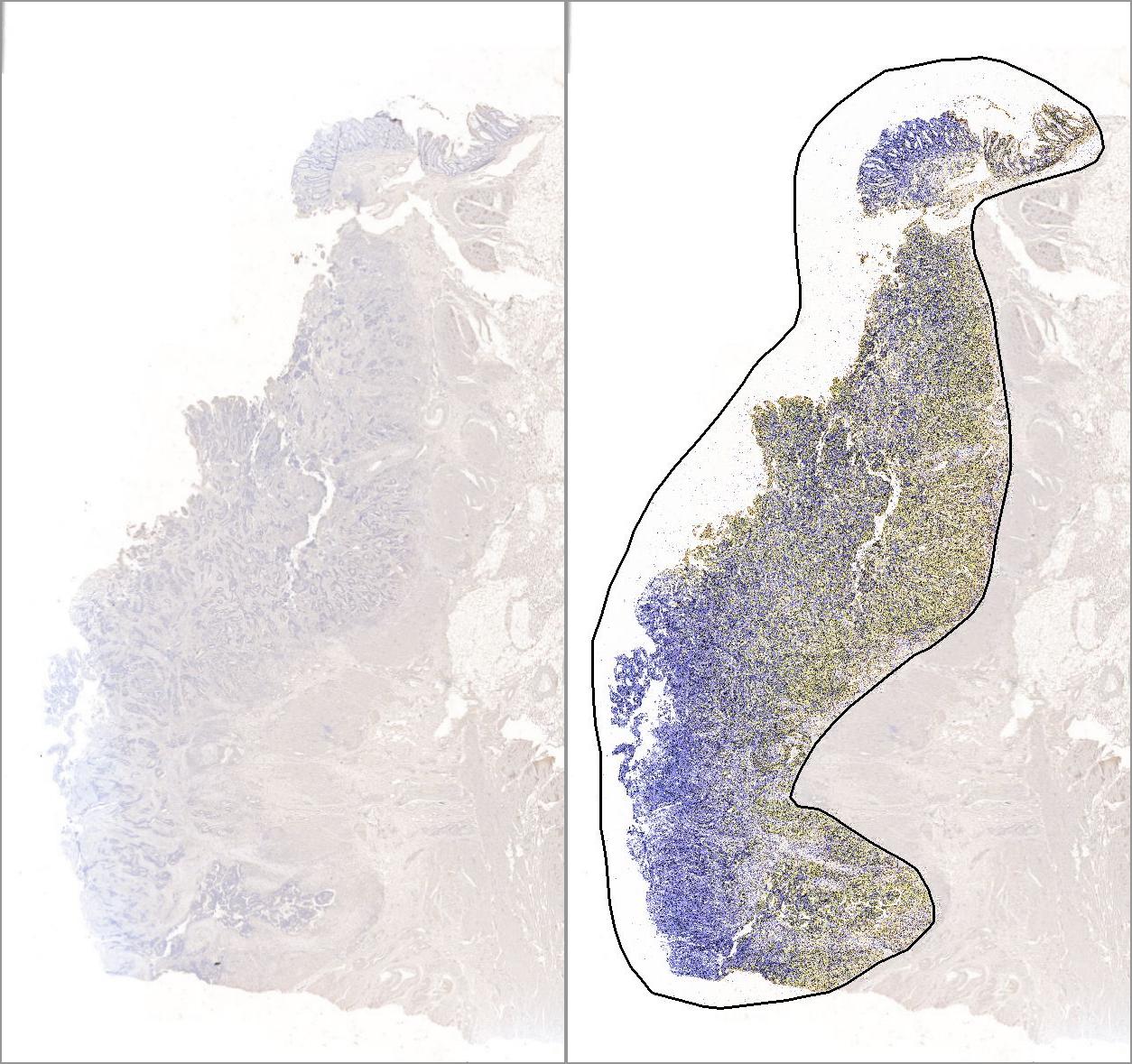


## 655459-17 CHMP7


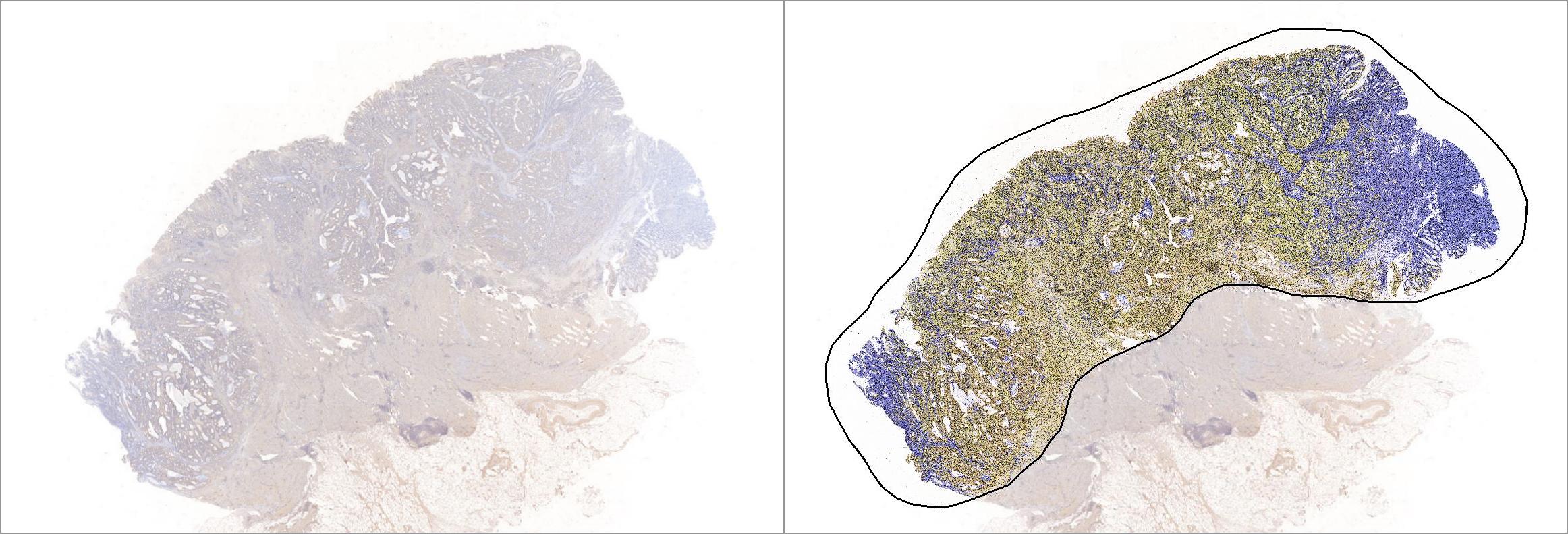


## 656433-12 CHMP7


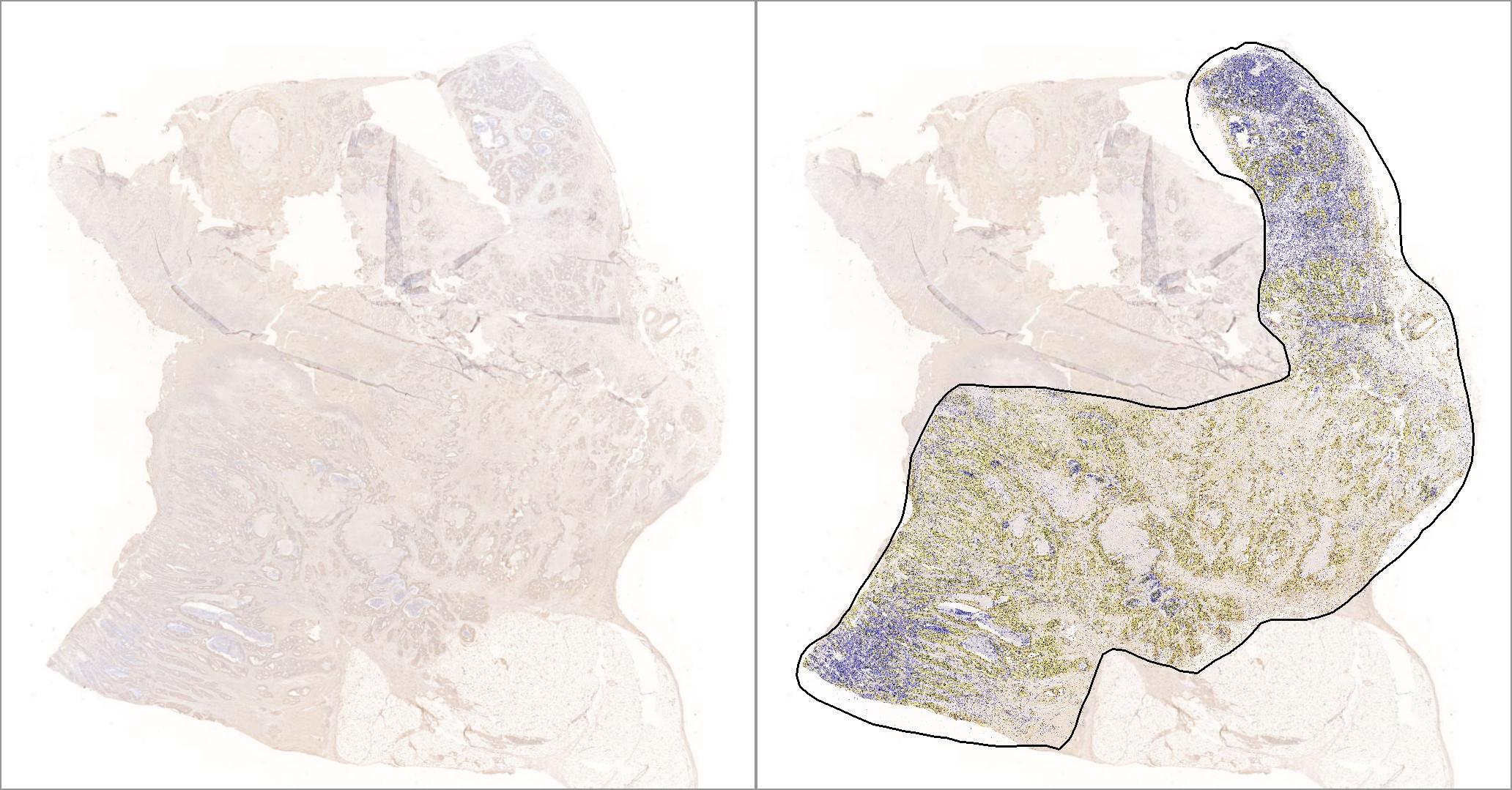


## 657275-17 CHMP7


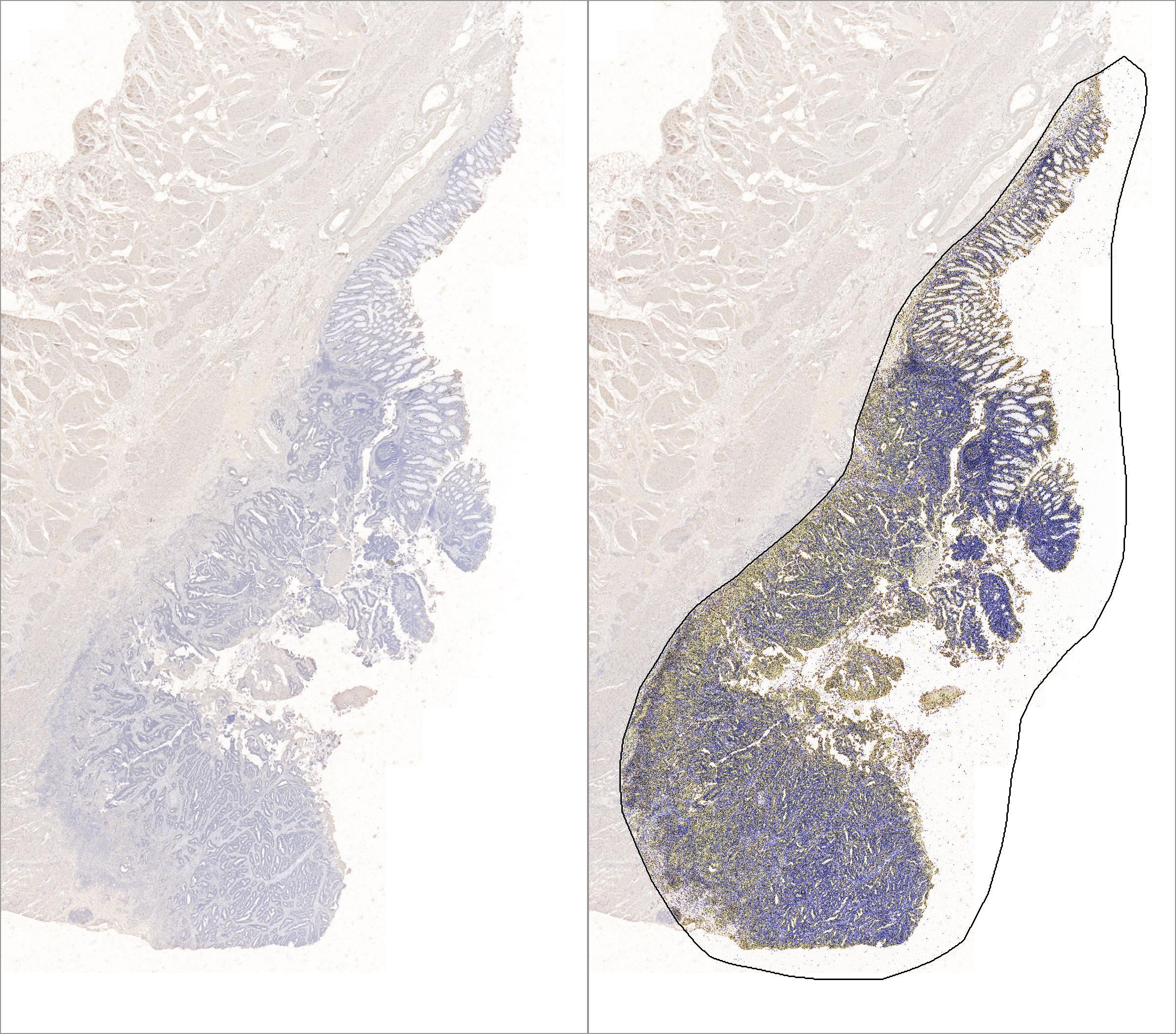


## 658264-13 CHMP7


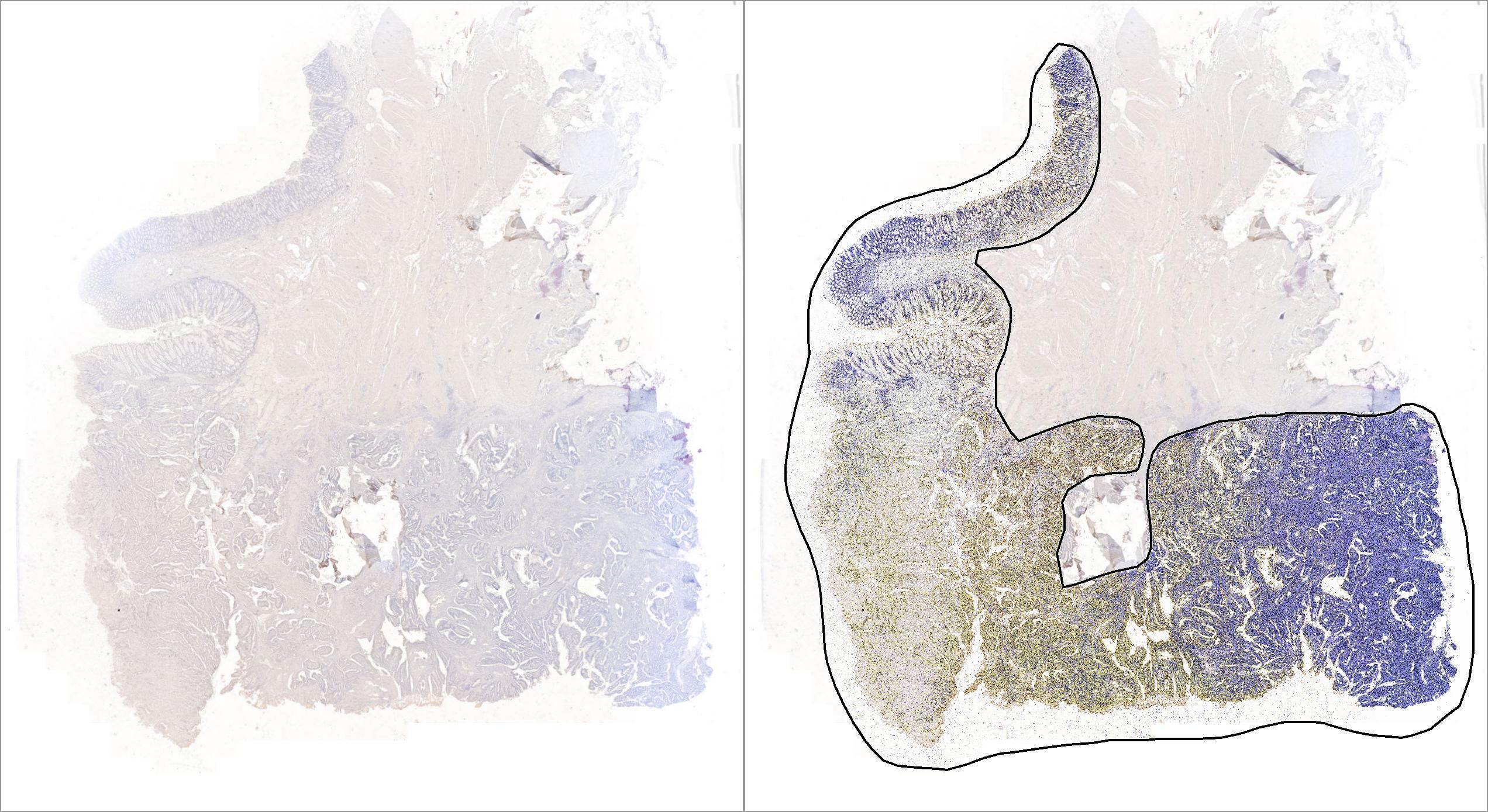


## 658988-25 CHMP7


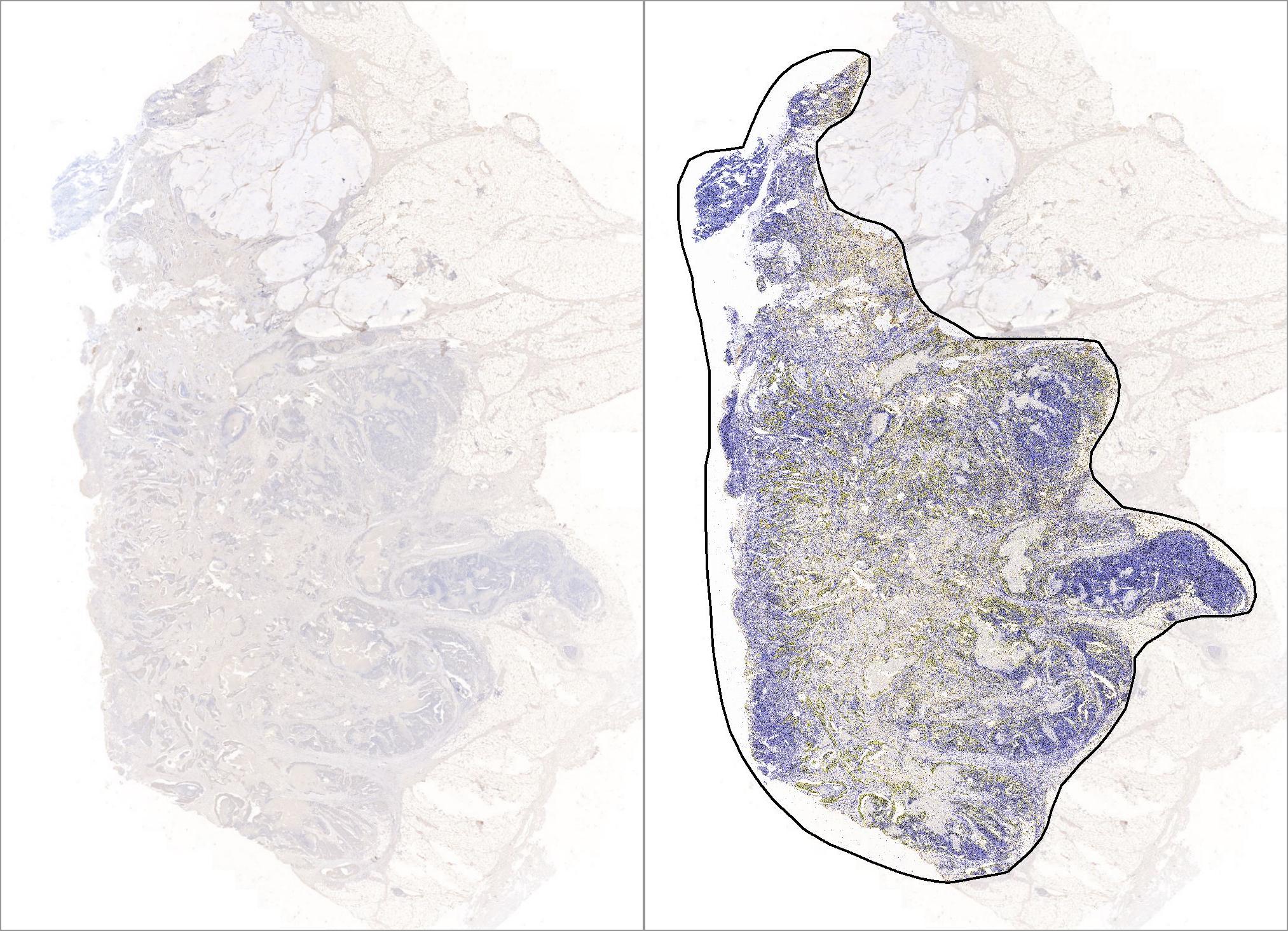


## 663169-12 CHMP7


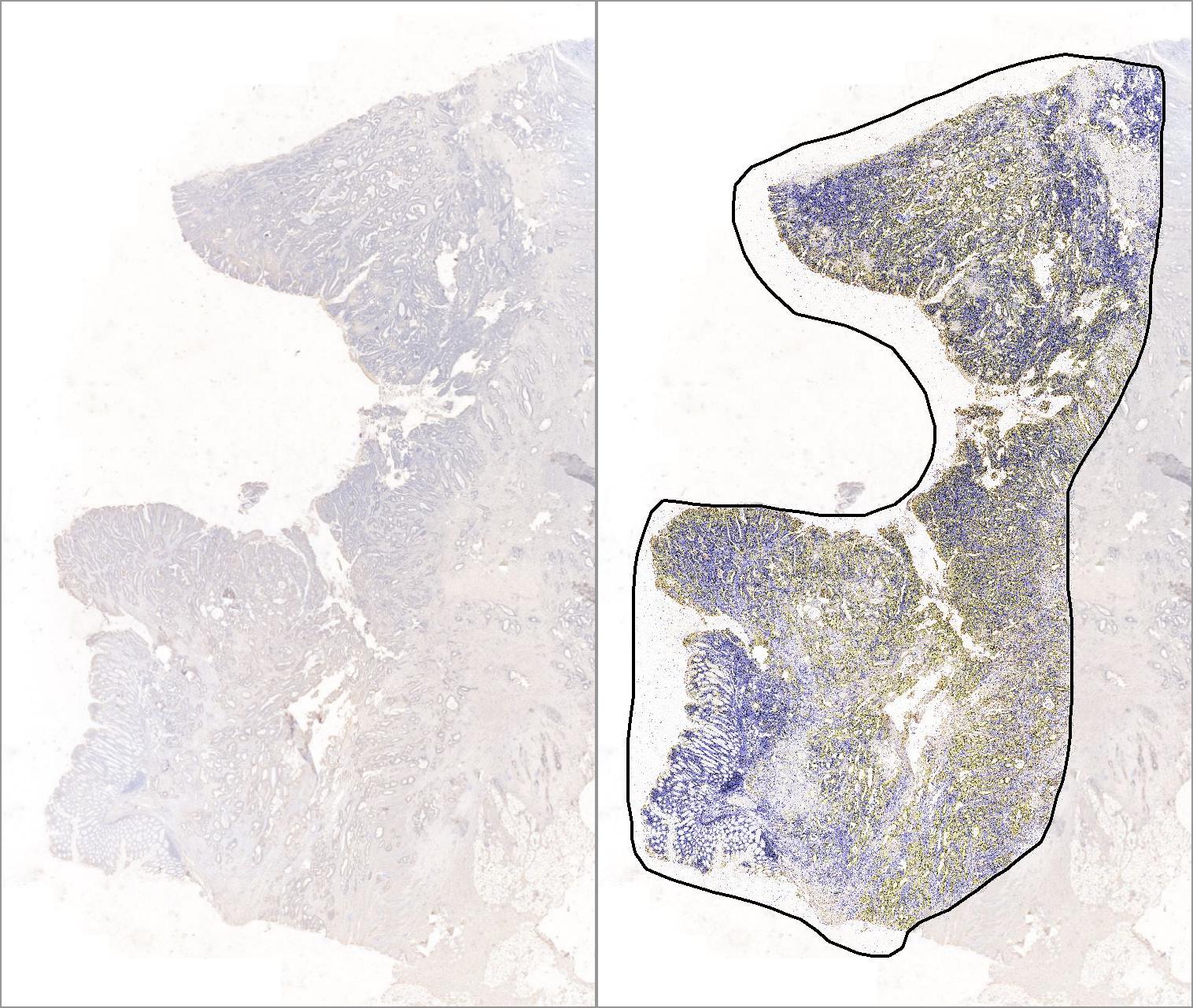


**590333-CHMP7**


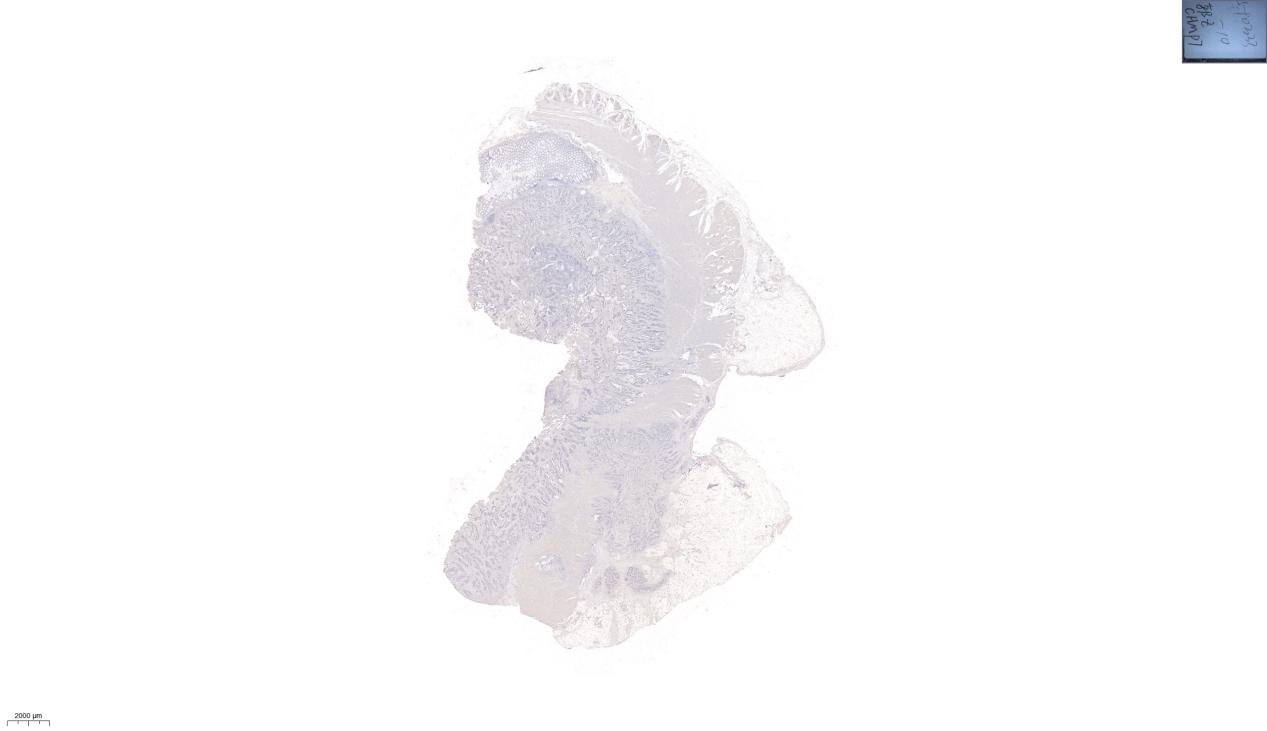


**620199-CHMP7**
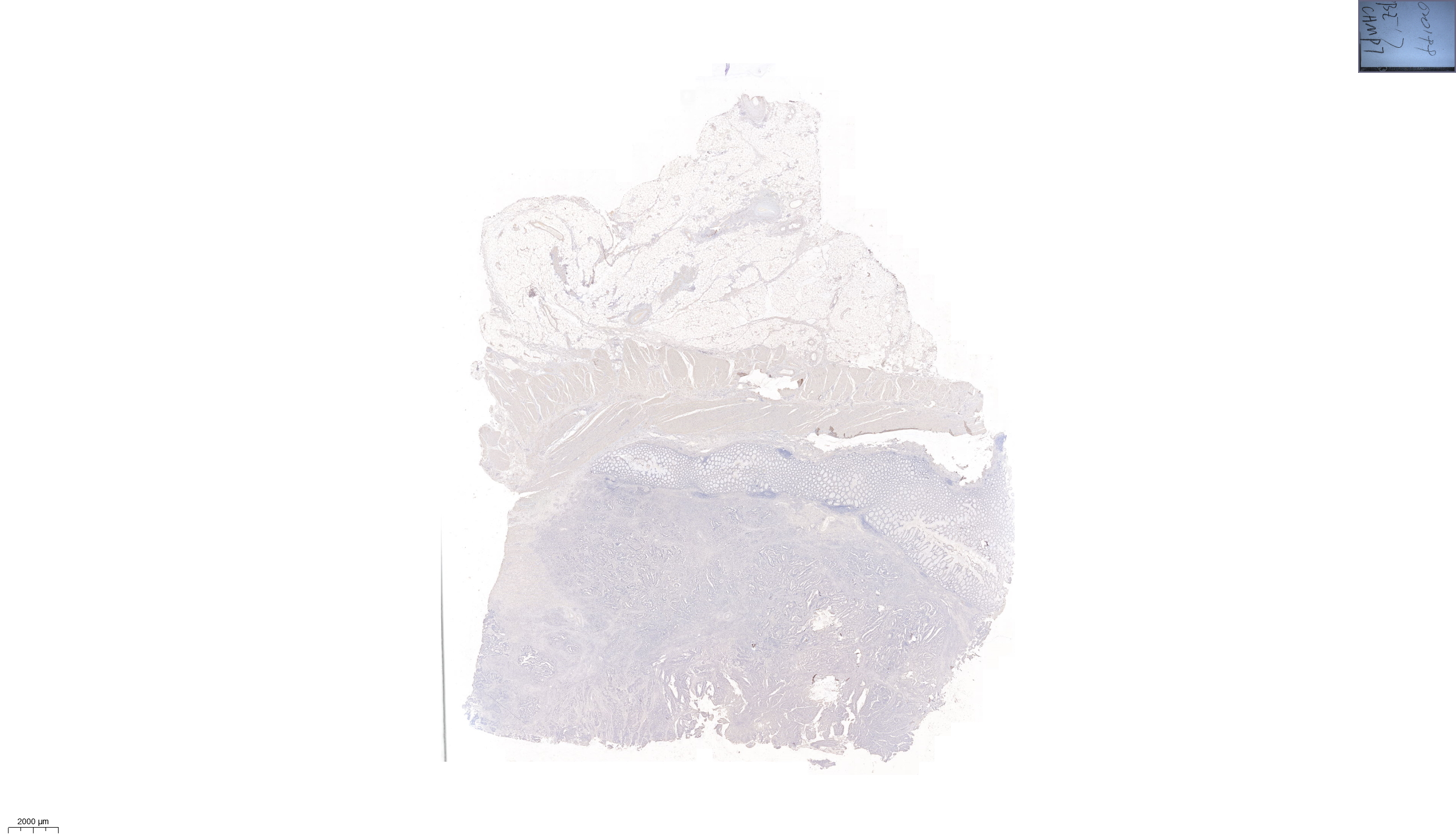


**617387-CHMP7**


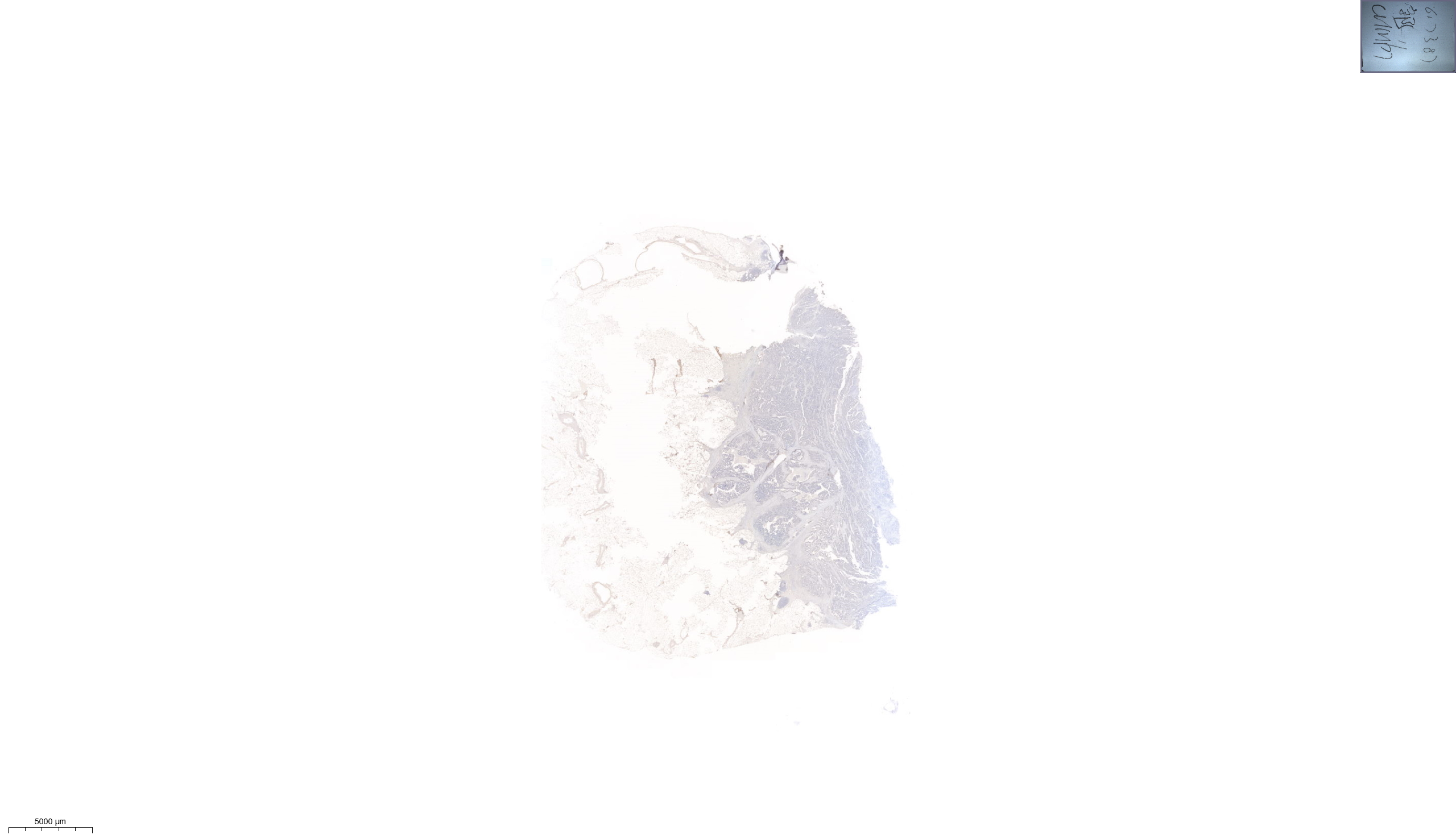


**620932-CHMP7**
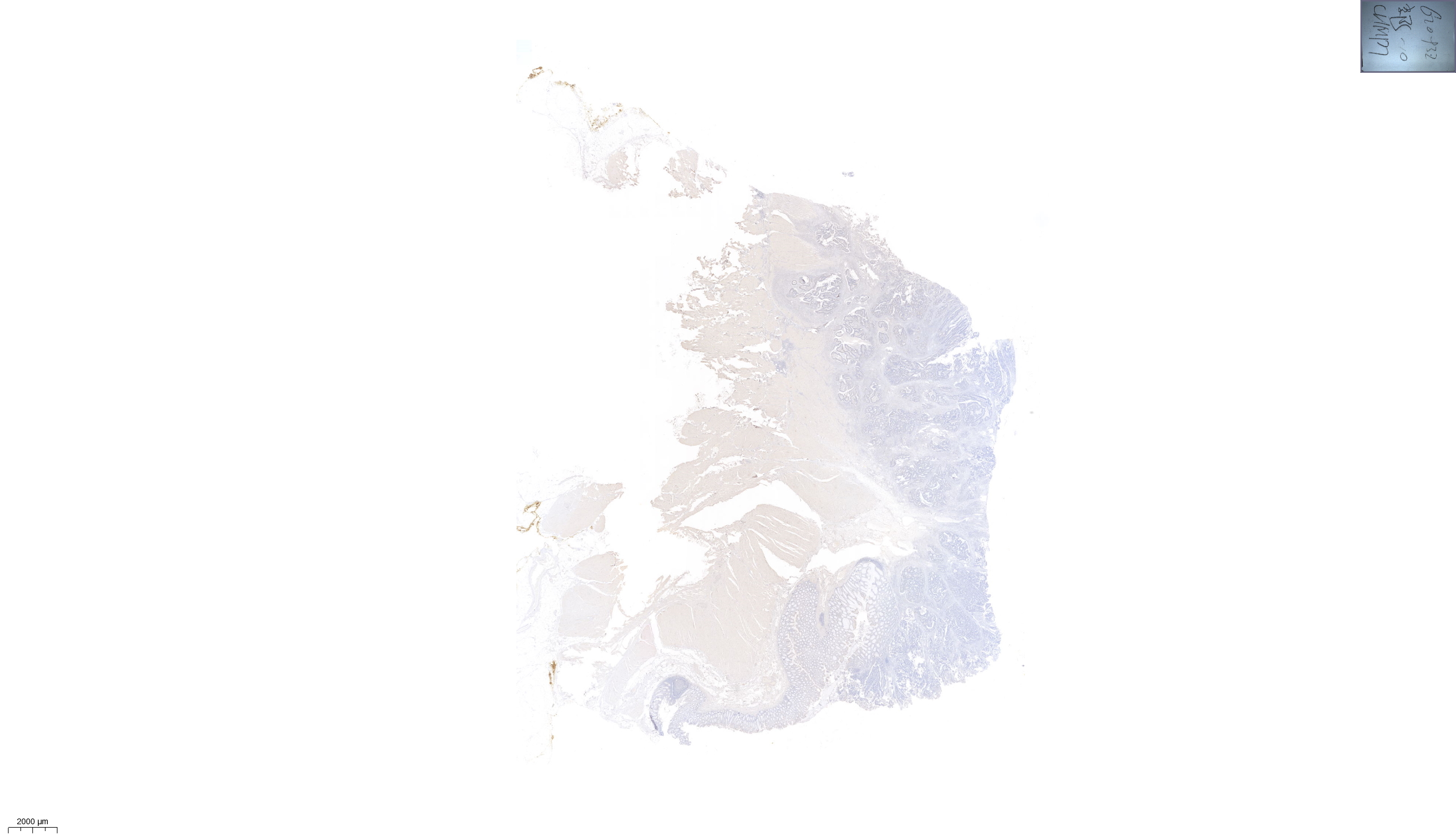


**626406-CHMP7**


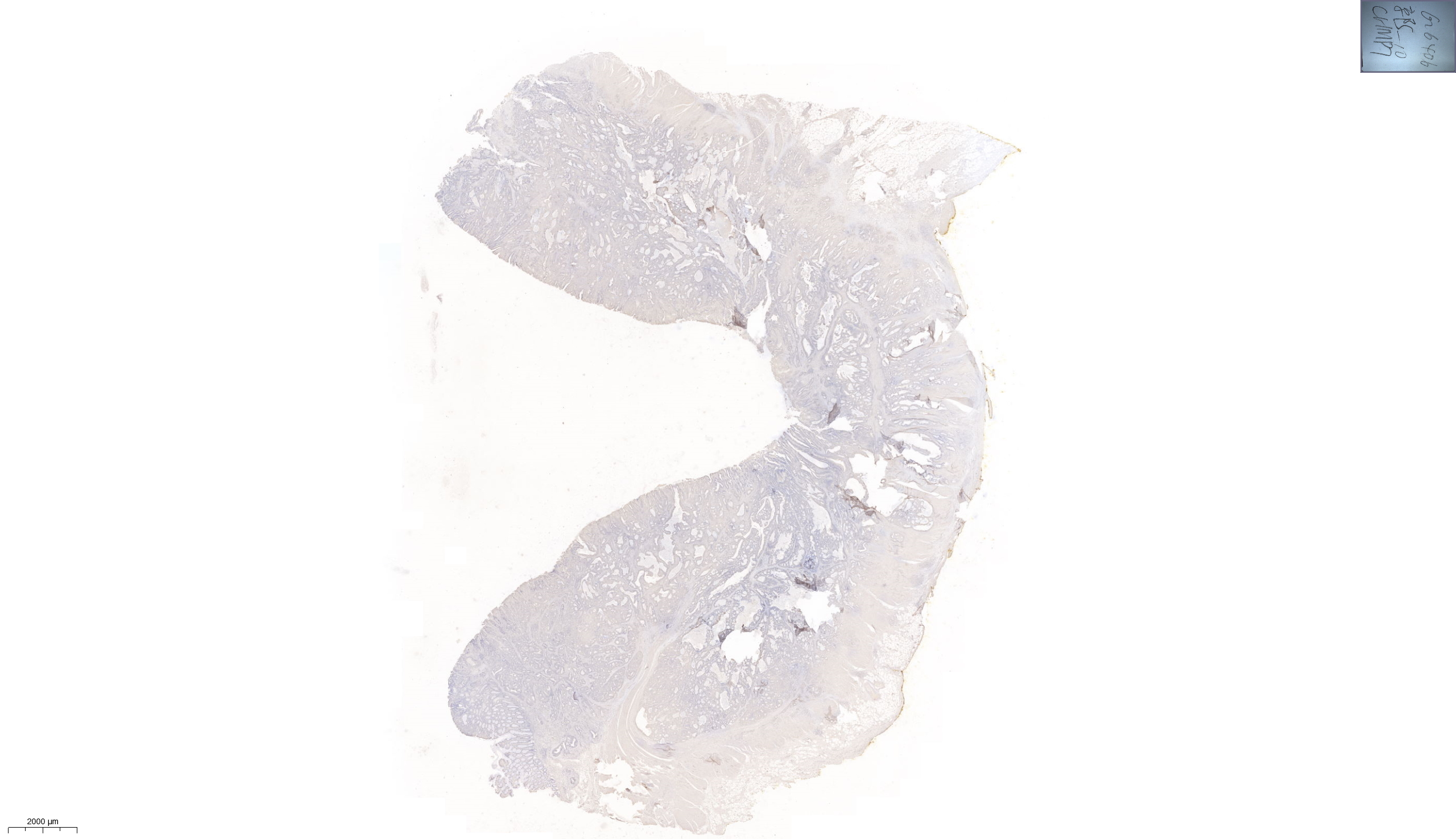


**632285-CHMP
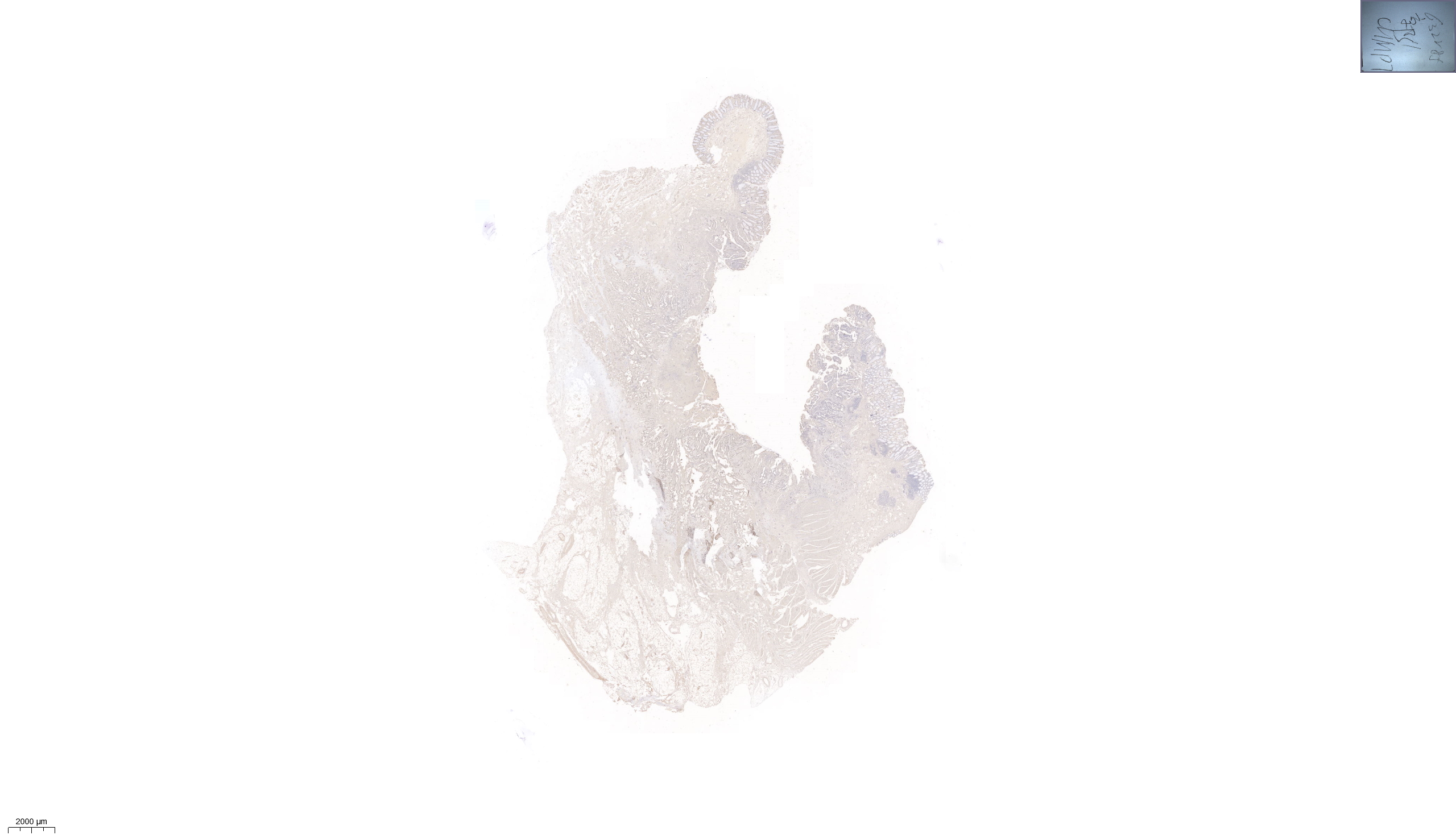
**

**646649-CHMP7**


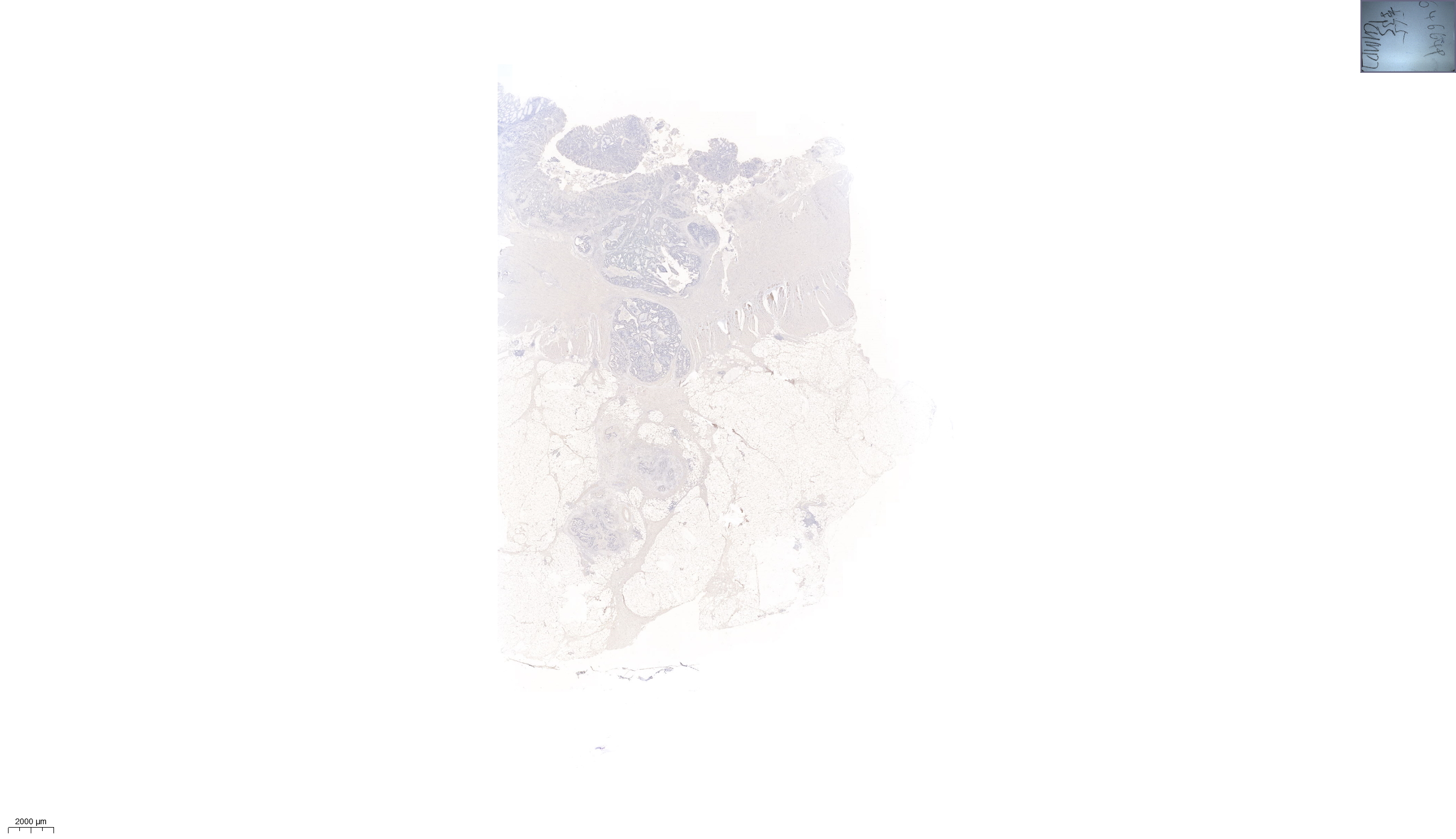


**646879-CHMP7**
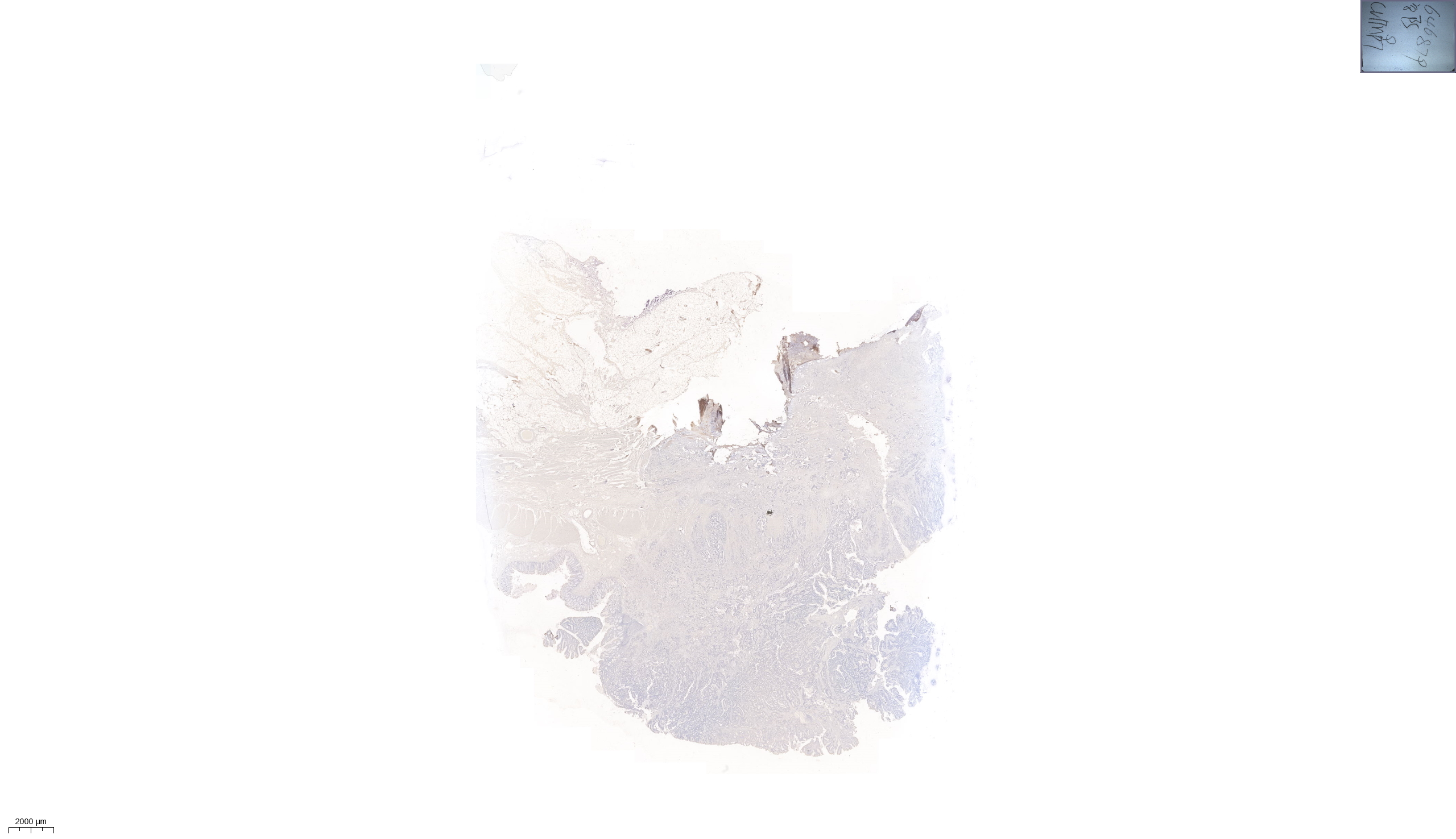


**651255-CHMP7**


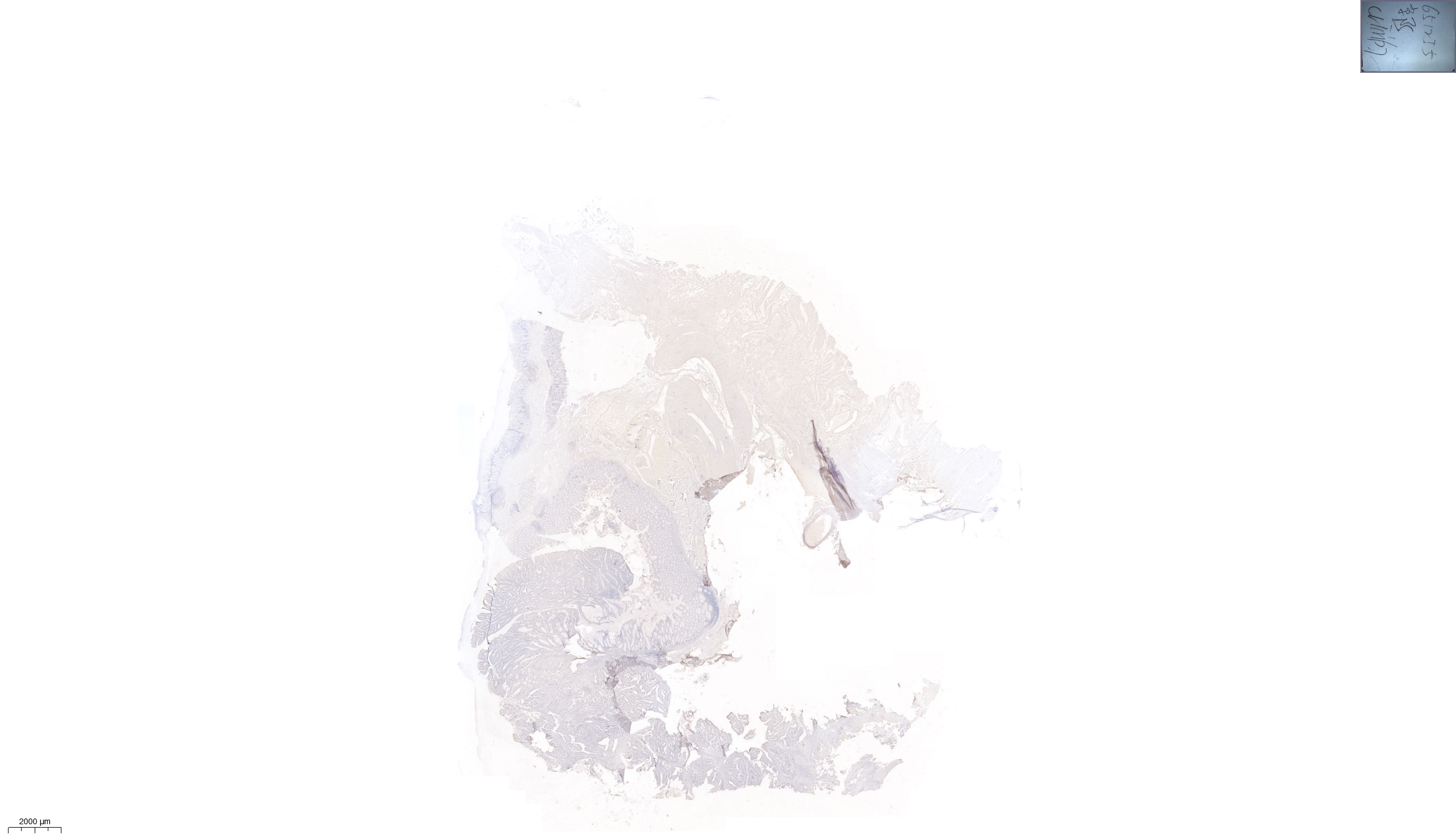


**656632-CHMP7**
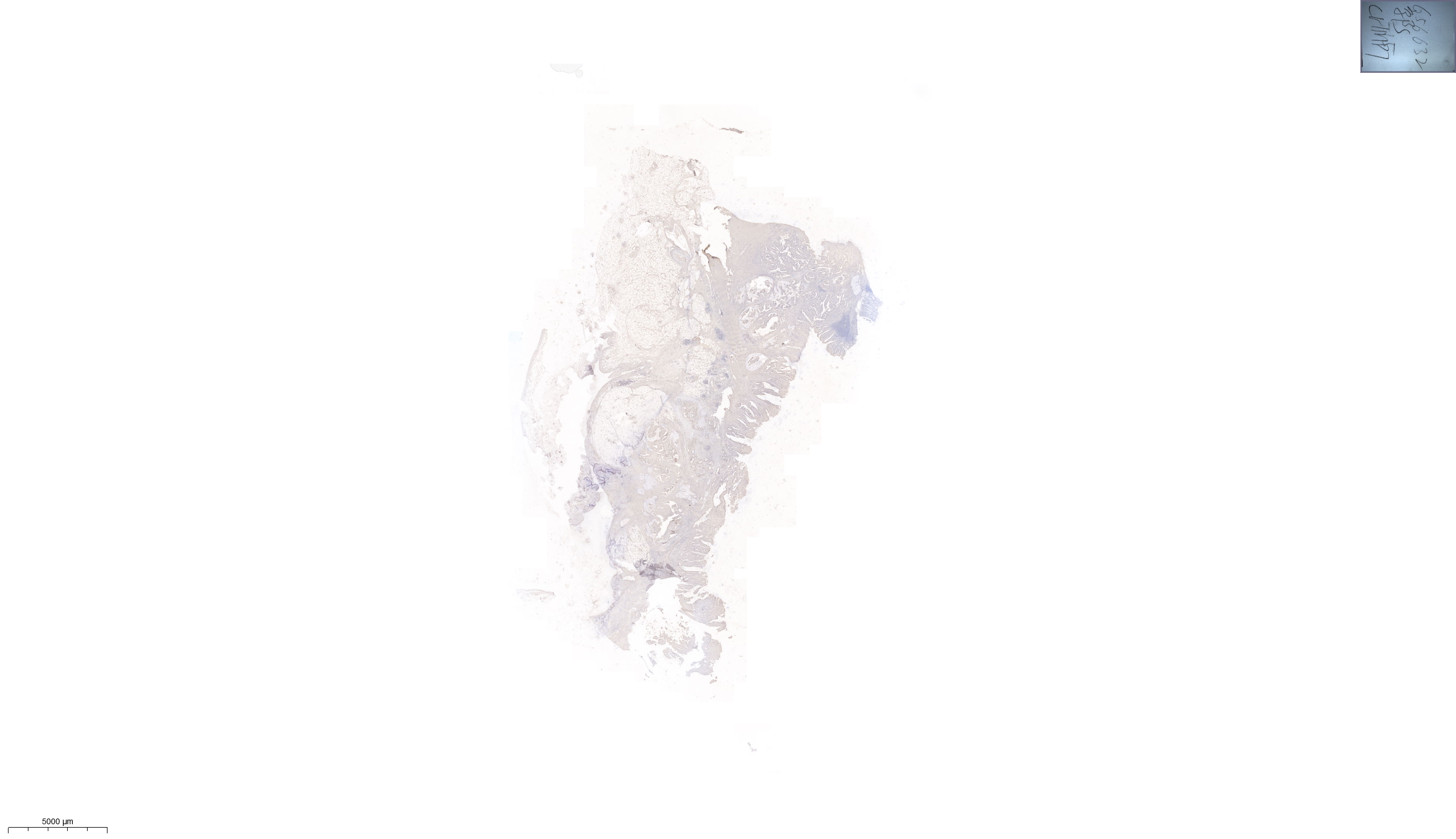

Supplement: Supplementary file 2 [file DataSheet1.ZIP › Fig2G-IHC slides.docx]
