# Supplementary material for: Identification of CHMP7 as a promising immunobiomarker for immunotherapy and chemotherapy and impact on prognosis of colorectal cancer patients
Source: Front Cell Dev Biol. 2023 Aug 30;11:1211843. doi: 10.3389/fcell.2023.1211843 (PMC10499328; doi:10.3389/fcell.2023.1211843)
Supplement: Supplementary file 3 [file DataSheet2.ZIP › Raw data introduction.docx]

Fig2

Fig2A：drawn by the website Opentarget (https://www.opentargets.org/)

Fig2B：Raw data in attachment

Fig2C-D：drawn by the website UALCAN (http://ualcan.path.uab.edu/)

Fig2E：Raw data and codes in attachment

Fig2F：Raw data and codes in attachment

Fig2G：Raw data in attachment

Fig3

Fig3A-B：drawn by the website cbioportal (https://www.cbioportal.org/)

Fig3C-E: Raw data in attachment and graphic received assistance from Xiantao Academic (https://www.xiantaozi.com/)

Fig4

Fig4A: Raw data in attachment

Fig4B: Raw data in attachment

Fig4C: drawn by the website GEPIA (http://gepia2.cancer-pku.cn/#index)

Fig4D: drawn by the website UALCAN (http://ualcan.path.uab.edu/)

Fig4E: Raw data in attachment

Fig5

Fig5A-C: drawn by the website OncoSpicing (http://www.oncosplicing.com/)

Fig5D：drawn by the website GEPIA (http://gepia2.cancer-pku.cn/#index)

Fig6

Fig6A: drawn by the website String (https://www.string-db.org/)

Fig6B: Raw data in attachment

Fig6C: drawn by the website GEPIA (http://gepia2.cancer-pku.cn/#index)

Fig6D: drawn by the website GEPIA (http://gepia2.cancer-pku.cn/#index)

Fig6E: Raw data in attachment

Fig7

Fig7A-B: Raw data in attachment

Fig7C-E: drawn by the website TISIDB (http://cis.hku.hk/TISIDB/)

Fig7F: drawn by the website TISMO (http://tismo.cistrome.org/)

Fig8

Fig8A: Raw data in attachment

Fig8B：Raw data in attachment

Fig8C：drawn by the website TIMER2 (http://timer.cistrome.org/)

Fig8D：drawn by the website SpatialDB (https://www.spatialomics.org/SpatialDB/search.php)

Fig8E：drawn by the website TISCH (http://tisch.comp-genomics.org/)

Fig8F：drawn by the website TISCH (http://tisch.comp-genomics.org/)

Fig9

Fig9A-B: drawn by the website TIMER2 (http://timer.cistrome.org/)

Fig9C: drawn by the website TIDE (http://tide.dfci.harvard.edu/query/)

Fig10

Fig10A: drawn by the website TIDE (http://tide.dfci.harvard.edu/query/)

Fig10B: drawn by the website TISMO (http://tismo.cistrome.org/)

Fig10C: drawn by the website TIDE (http://tide.dfci.harvard.edu/query/)

Fig10D: drawn by the website GSCA (http://bioinfo.life.hust.edu.cn/web/GSCALite/)

Fig10E: drawn by the website ROCplotter (http://www.rocplot.org/site/index)

Sup1: Raw data in attachment

Sup2: drawn by the website OncSplicing (http://www.oncosplicing.com/)

Sup3: drawn by the website GEPIA (http://gepia2.cancer-pku.cn/#index)

Sup4: Raw data in attachment

Sup5: drawn by the website RNAact Drug <(http://bio-bigdata.hrbmu.edu.cn/RNAactDrug/index.jsp>)

The data visualization in the manuscript obtained assistance from Xiantao Academic (<https://www.xiantaozi.com/>) and Sangerbox (http://vip.sangerbox.com/).
